# Evolution of the Metazoan Mitochondrial Replicase

**DOI:** 10.1093/gbe/evv042

**Published:** 2015-03-03

**Authors:** Marcos T. Oliveira, Jani Haukka, Laurie S. Kaguni

**Affiliations:** ^1^Institute of Biosciences and Medical Technology, University of Tampere, Finland; ^2^Departamento de Tecnologia, Faculdade de Ciências Agrárias e Veterinárias, Universidade Estadual Paulista “Júlio de Mesquita Filho,” Jaboticabal, SP, Brazil; ^3^Department of Biochemistry and Molecular Biology and Center for Mitochondrial Science and Medicine, Michigan State University

**Keywords:** mitochondria, mitochondrial DNA replication, structural evolution, mitochondrial replicase, pol γ

## Abstract

The large number of complete mitochondrial DNA (mtDNA) sequences available for metazoan species makes it a good system for studying genome diversity, although little is known about the mechanisms that promote and/or are correlated with the evolution of this organellar genome. By investigating the molecular evolutionary history of the catalytic and accessory subunits of the mtDNA polymerase, pol γ, we sought to develop mechanistic insight into its function that might impact genome structure by exploring the relationships between DNA replication and animal mitochondrial genome diversity. We identified three evolutionary patterns among metazoan pol γs. First, a trend toward stabilization of both sequence and structure occurred in vertebrates, with both subunits evolving distinctly from those of other animal groups, and acquiring at least four novel structural elements, the most important of which is the HLH-3β (helix-loop-helix, 3 β-sheets) domain that allows the accessory subunit to homodimerize. Second, both subunits of arthropods and tunicates have become shorter and evolved approximately twice as rapidly as their vertebrate homologs. And third, nematodes have lost the gene for the accessory subunit, which was accompanied by the loss of its interacting domain in the catalytic subunit of pol γ, and they show the highest rate of molecular evolution among all animal taxa. These findings correlate well with the mtDNA genomic features of each group described above, and with their modes of DNA replication, although a substantive amount of biochemical work is needed to draw conclusive links regarding the latter. Describing the parallels between evolution of pol γ and metazoan mtDNA architecture may also help in understanding the processes that lead to mitochondrial dysfunction and to human disease-related phenotypes.

## Introduction

Mitochondrial DNA (mtDNA) replication is accomplished by the sole DNA polymerase found in animal mitochondria, DNA polymerase γ (pol γ) (reviewed in Kaguni 2004), which functions as part of a larger replication machinery called the mtDNA replisome. The identity of all of the components of the mtDNA replisome is still unknown, but biochemical studies (Korhonen et al. 2003, 2004; Oliveira and Kaguni 2010, 2011) have shown that a group of mitochondrial proteins can interact functionally to promote DNA synthesis in vitro, forming the minimal mtDNA replisome: The mtDNA helicase, also known as Twinkle in humans, unwinds the duplex DNA at the replication fork; pol γ catalyzes nascent DNA synthesis on both the leading and lagging DNA strands; and the mitochondrial single-stranded DNA-binding protein (mtSSB) coordinates their functions while binding and stabilizing the single-stranded DNA template (Korhonen et al. 2004; Oliveira and Kaguni 2011). This scenario, as in most DNA replication events, requires the presence of short RNA molecules for priming of DNA synthesis by the DNA polymerase, which in animal mitochondria might be achieved by the action of the mitochondrial RNA polymerase (Wanrooij et al. 2008; Fuste et al. 2010; Reyes et al. 2013) and/or the newly identified primase PrimPol (Garcia-Gomez et al. 2013).

Animal pol γ is a hetero-oligomeric enzyme, in most known cases: The catalytic core, pol γ-α (also known as POLG or PolGA), contains both the 5′–3′ DNA polymerase and 3′–5′ exonuclease activities of the holoenzyme, whereas the accessory subunit, pol γ-β (or POLG2, PolGB), serves as a processivity factor, enhancing the interactions between the holoenzyme and the DNA substrate (Lewis et al. 1996; Wang et al. 1997; Carrodeguas et al. 1999; Lim et al. 1999). The catalytic core is a member of the family A DNA polymerase group, to which bacterial DNA polymerase I and the catalytic core of bacteriophage T7 DNA polymerase (gp5) also belong (reviewed in Kaguni 2004); pol γ-α shares substantial structural and functional properties with both Pol I and T7 Pol. Notably, Pol I is not the replicative DNA polymerase in bacteria. It is a single-subunit enzyme that lacks the high fidelity and processivity of the holoenzyme form of T7 Pol, which acquires these properties as a result of the association of the catalytic core with the bacterial protein thioredoxin that serves as its accessory subunit. Although the presence of pol γ as the mitochondrial replicase appears to be conserved among the metazoans (and other eukaryotes), important variations in its structure have been described that may impact the mode in which mtDNA is replicated. It has long been known that pol γ of *Drosophila melanogaster*, one of the best studied insect model organisms, is a heterodimer comprising one catalytic subunit and a single accessory subunit (Wernette and Kaguni 1986; Olson et al. 1995; Wang and Kaguni 1999). On the other hand, the human and mouse holoenzymes have a heterotrimeric conformation, consisting of one pol γ-α and a dimeric pol γ-β (Carrodeguas et al. 2001; Yakubovskaya et al. 2006; Lee et al. 2009). Interestingly, the mtDNA of the nematode *Caenorhabditis elegans* appears to be replicated by a machinery containing only a single subunit pol γ (only pol γ-α) (Bratic et al. 2009; Addo et al. 2010), which resembles the catalytic core in other eukaryotes, such as that of the yeast *Saccharomyces cerevisiae* (Foury 1989). The accessory subunit of pol γ, which indeed appears to be present only in Metazoa, has a remarkable evolutionary origin, because amino acid sequence alignments, phylogenetic inferences, and general protein structure demonstrate its homology to class II aminoacyl-tRNA synthetases (Fan et al. 1999, 2006; Carrodeguas et al. 2001; Wolf and Koonin 2001).

We sought to explore the sequence and structural diversity of pol γ in the animal kingdom, taking advantage of the current increase in nuclear genomes and transcriptomes for which complete sequences are available in public databases. We retrieved as many animal pol γ-α and -β gene sequences as are available and performed in silico analyses to infer their molecular evolutionary history, taking into account the substantial biochemical and structural data reported by our group and others. Here we report the oligomeric plasticity of animal pol γ, the finding of new structural elements, and the distinct rates of molecular evolution for different taxa, which may reflect differences in the fundamental mechanisms of mtDNA replication. We discuss our findings in the context of mitochondrial genome diversity, structure, replication, and evolution.

## Materials and Methods

### Searches for Animal pol γ-α and -β Homologs and Multiple Sequence Alignments

TBLASTN searches (Altschul et al. 1990) in the NCBI (National Center for Biotechnology Information) nonredundant sequence database were performed using the translated mRNA reference sequences from *Homo sapiens* (pol γ-α, NM_001126131.1; pol γ-β, NM_007215.3) and *D. melanogaster* (pol γ-α, NM_057473.3; pol γ-β, FJ635829.1) as queries. To retrieve sequences from Porifera, Placozoa, Cnidaria, Mollusca, and Hemichordata species, complementary HMMR3 BLAST (Basic Local Alignment Search Tool) searches (Eddy 2011) were performed (http://toolkit.lmb.uni-muenchen.de/hmmer3, last accessed August 2014), followed by BLAST searches against the Ensembl Metazoa database (http://metazoa.ensembl.org, last accessed August 2014), allowing the inclusion of missing exons. Most sequences from Nematoda species were retrieved from genomic scaffolds with no gene models deposited in the 959 Nematode Genomes databank (http://www.nematodes.org/nematodegenomes, last accessed August 2014), followed by manual processing of the exons. A Python script utilizing functions of the Biopython library (Cock et al. 2009) was developed to extract the coding sequences and other specific information from the original files retrieved from the diverse databanks. The script can be provided by the authors upon request. Highly divergent sequences were tested for true orthology by using them as queries for BLAST searches against the human genome database and by predicting possible mitochondrial localization using the TargetP service (Emanuelsson et al. 2007). The complete set of sequences retrieved is shown in supplementary table S1, Supplementary Material online.

Because of the overrepresentation by mammalian and *Drosophila* sequences, this data set was reduced before the alignments were performed to diminish bias in interpretation of the results. The multiple amino acid sequence alignments were performed with the software MAFFT (Katoh and Toh 2008), using the G-INSi and E-INSi algorithms for the pol γ-α and -β sequences, respectively, and are shown in supplementary figures S1 and S2, Supplementary Material online.

### Phylogenetic Inferences

To create the input files for the phylogenetic inferences, the software PAL2NAL (Suyama et al. 2006) was used to convert the amino acid sequence alignments into codon-based nucleotide alignments, which were then converted to NEXUS format. The phylogenetic trees were inferred using the Bayesian algorithm built in the software MrBayes, version 3.2.2 (Ronquist et al. 2012). The consensus tree, run with 200,000 cycles for pol γ-α and 1 million cycles for pol γ-β, was set to the 50-majority rule, gamma variation was expected among the sites and Generalized Time Reversible model was used; other parameters were kept as default.

### Modeling of Protein Structure

The structure of the disordered regions in the human pol γ-α crystallography data (PDB accession number 3IKM, chain A) was predicted with the software I-TASSER (Bazzoli et al. 2011), using default parameters. I-TASSER was also used with default parameters to model the whole structure of pol γ-α from *C. elegans* (using the human pol γ crystal structure 3IKM:A), and of pol γ-β from *Strongylocentrotus purpuratus*, *Ciona intestinalis,* and *Trichoplax adhaerens* (using the human pol γ-β crystal structure 2G4C:A). *Drosophila melanogaster* pol γ-β structure was modeled with the software MODELLER, version 9.12 (Sali and Blundell 1993), using the 2G4C:A file as template and the multiple sequence alignment (MSA) shown in supplementary figure S2, Supplementary Material online, as a parameter. The selected models were evaluated by the *Z*-scores, the discrete optimized protein energy values, and the residue error plot (SwissProt website, http://swissmodel.expasy.org/workspace/index.php?func=tools_structureassessment1, last accessed August 2014). Structures and models were analyzed and figures were produced using Pymol (www.pymol.org, last accessed August 2014).

## Results

Our searches of public genomic sequence databases found nonredundant sequences for pol γ-α and -β of 62 and 52 animal species, respectively (supplementary table S1, Supplementary Material online). The resulting data set is overrepresented by sequences from insect and vertebrate (especially mammalian) species due to the bias in the databases. Animal groups in the Protostomia clade, other than Arthropoda and Nematoda, were most often absent, except for one pol γ-α sequence from Mollusca. Nonetheless, we obtained sequences from key species of basal groups of Metazoa, such as Porifera, Placozoa and Cnidaria, and basal and sister groups of Chordata, such as Tunicata, Cephalochordata, Hemichordata and Echinodermata, which are crucial for the analyses described below.

**Fig. 1.—:**
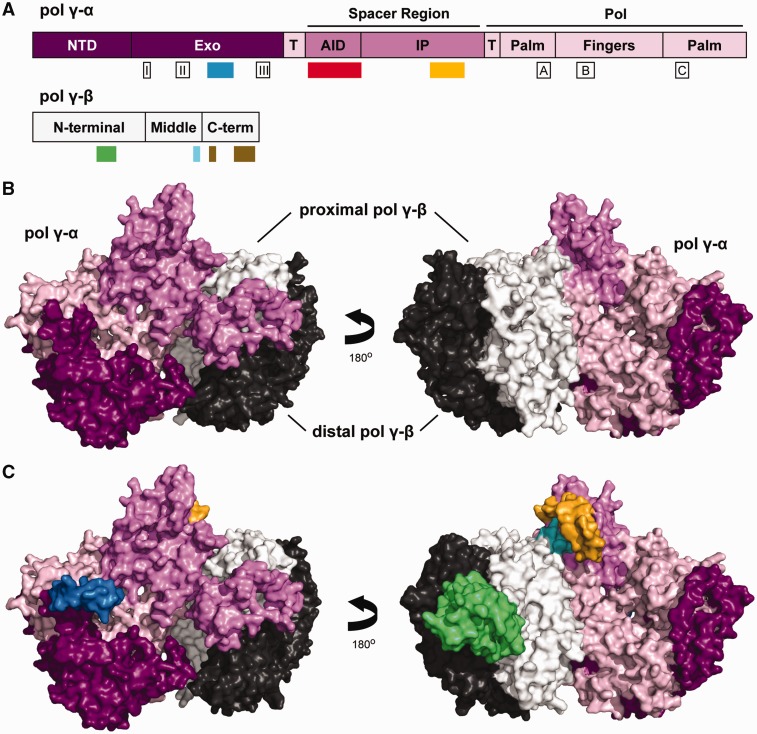
Schematics of animal (vertebrate) pol γ-α and -β sequence and structure. (*A*) Representation of the amino acid sequence of the pol γ-α and -β polypeptides, showing the protein domains, subdomains, conserved motifs, and new motifs proposed in this work. For pol γ-α: NTD, the N-terminal domain for which no functional data are available; Exo, the exonuclease domain responsible for the 3′–5′ exonuclease activity that edits misincorporated nucleotides and increases the fidelity of DNA synthesis several-hundred fold; AID, the accessory-interacting determinant subdomain that provides the primary contacts between the catalytic core and the accessory subunit; IP, the intrinsic processivity subdomain that contributes to the ability of the catalytic core to polymerize multiple nucleotides in a single enzyme binding cycle; Pol, the DNA polymerase domain responsible for the 5′–3′ DNA polymerase activity; T, the bipartite thumb subdomain (according to Lee et al. 2009) that contributes to template–primer DNA binding; boxes I–III, the conserved motifs Exo I–III that form the 3′–5′ exonuclease active site; boxes A–C, the conserved motifs Pol A–C that comprise the 5′–3′ polymerase active site (for more detailed descriptions of each of these features, see Kaguni [2004] and Euro et al. [2011]; blue box, the new vertebrate Exo motif; red box, the region absent in the nematode species; and orange box, the new vertebrate IP motif [see the text for details]. For pol γ-β, the domain designations follow that described by [Fan and Kaguni 2001; Fan et al. 2006]. Green box, the vertebrate dimerization [HLH-3β] interface; cyan box, the vertebrate M loop [see text for details]; and brown boxes, the conserved hydrophobic regions for pol γ-α interaction [according to Lee et al. 2009]). All structural elements are represented to scale, except motifs Exo I–III and Pol A–C. (*B*) Surface representation of the crystal structure of the human pol γ apo-holoenzyme (3IKM; Lee et al. 2009), highlighting the interactions among the subunits and pol γ-α functional domains. Structures are colored as shown in (*A*); dark gray, distal pol γ-β. (*C*) Surface representation of the crystal structure of the human pol γ apo-holoenzyme (3IKM; Lee et al. 2009), highlighting the motifs and domains identified in this study. The structures are colored as shown in (*A*) and (*B*).

We excluded several sequences of mammalian and *Drosophila* species from our pol γ-α and -β data sets to balance the taxa representation, and also a few of the BLAST results because they contained only partial gene sequences (see supplementary table S1, Supplementary Material online). Supplementary figure S1, Supplementary Material online, shows the alignment of 43 pol γ-α amino acid sequences plus the outgroup (the sequence from *S. cerevisiae* pol γ-α, accession number NM_001183750.1). The alignment of 35 pol γ-β amino acid sequences plus the outgroup (the glycyl-tRNA synthetase sequence from the bacterium *Thermus thermophilus*, accession number AJ222643.1) is shown in supplementary figure S2, Supplementary Material online. The number of pol γ-β sequences retrieved was lower than that for pol γ-α mainly because of the absence of the pol γ-β gene in the genome of nematode species, as discussed below. In addition, we were unable to find complete pol γ-β gene sequences for the poriferan *Amphimedon queenslandica*, the mollusk *Crassostrea gigas*, and the crustacean *Daphnia pulex*. Considering that the pol γ-α sequences from these species do have a potential to form an accessory-interacting determinant (AID) structure (see below), our failure to find the corresponding pol γ-β might be because of the current low coverage of their genome/transcriptome sequences. A summary of our most interesting findings is presented in figure 1, along with schematics of the catalytic and accessory subunit polypeptides. We identified specific features for all sequences retrieved to indicate that our data set most likely consists of true orthologs of the catalytic and accessory subunits of the mitochondrial replicase, and not random genes coding for other family A DNA polymerases or aminoacyl-tRNA synthetases, respectively: 1) Conserved active site motifs in the exonuclease (Exo I–III) and polymerase (Pol A–C) domains of pol γ-α (reviewed in Kaguni 2004) (supplementary fig. S1, Supplementary Material online) that are shared among family A DNA polymerases; 2) conserved pol γ-α-specific sequences in the spacer region and polymerase domains of pol γ-α (Lewis et al. 1996; Lecrenier et al. 1997; Kaguni 2004); 3) conserved sequence for the AID subdomain of pol γ-α (except in nematodes), which is a pol γ-α-specific feature of metazoans (reviewed in Kaguni 2004) (see below); 4) conserved hydrophobic residues in the C-terminal region of pol γ-β that are most relevant for the interactions with the pol γ-α AID, and are found almost exclusively in pol γ-βs (Fan et al. 1999; Carrodeguas et al. 2001; Kaguni 2004; Lee et al. 2009); and 5) absence in pol γ-β of the residues required for dimerization of class II aminoacyl-tRNA synthetase (Logan et al. 1995; Arnez et al. 1999).

### Distinct Rates of Evolutionary Changes in pol γ-α and -β Sequences

**Fig. 2.—:**
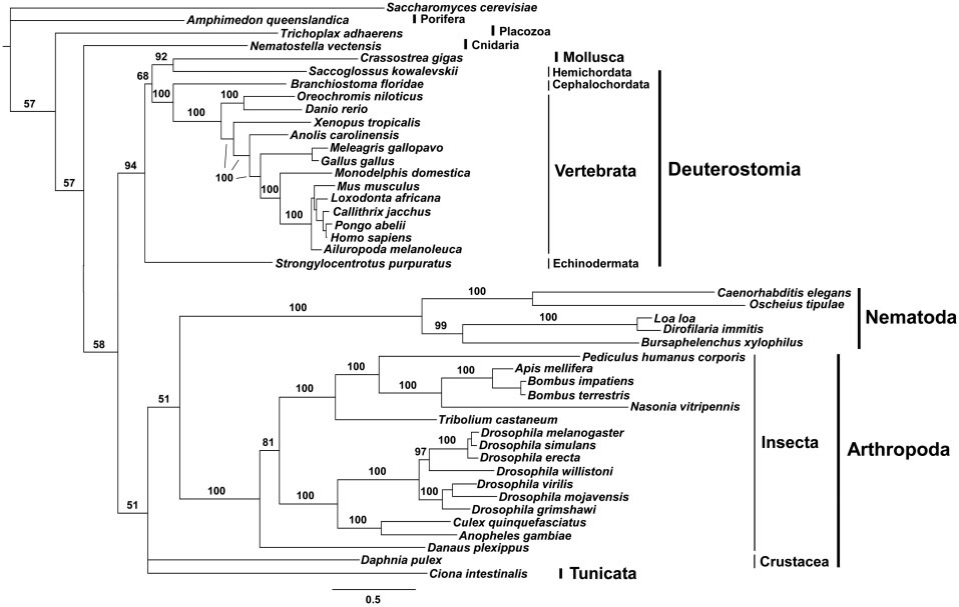
Bayesian phylogenetic inference for animal pol γ-α nucleotide sequences. The outgroup sequence used was the pol γ-α (*mip-1*) from the yeast *S. cerevisiae* (accession number NM_001183750.1). The 50% majority-rule consensus tree was inferred using MrBayes 3.2, as described under Materials and Methods. Bayesian posterior probability values are indicated for almost all nodes. The scale bar indicates substitutions per site.

Phylogenetic inferences with the pol γ-α nucleotide sequences using Bayesian analysis (fig. 2) reproduced moderately well the currently accepted relationships among animal taxa (Philippe et al. 2009), with few exceptions, and provided important findings regarding the evolution of the gene. First, it is clear that the sequences from nematodes have evolved at a higher rate than those from insect species, which in turn have accumulated more substitutions than the sequences from vertebrates. Second, Tunicata, which is represented only by the sequence from *Ci. intestinalis* (Ascidiacea: Cionidae), was grouped within the pol γ-α sequences from Arthropoda. Finally, the branches for the pol γ-α sequences from Deuterostomia (vertebrates and sister groups), excluding *Ci. intestinalis*, and from the mollusk *Cr. gigas* were as short as those for the sequences from basal animal groups, such as Porifera (*A. queenslandica*), Placozoa (*T. adhaerens*), and Cnidaria (*Nematostella vectensis*). In combination, this may indicate that most deuterostome pol γ-αs retained more ancestral characters, whereas pol γ-α sequences from nematodes, arthropods (especially insects), and tunicates have diverged considerably from the original enzyme.

**Fig. 3.—:**
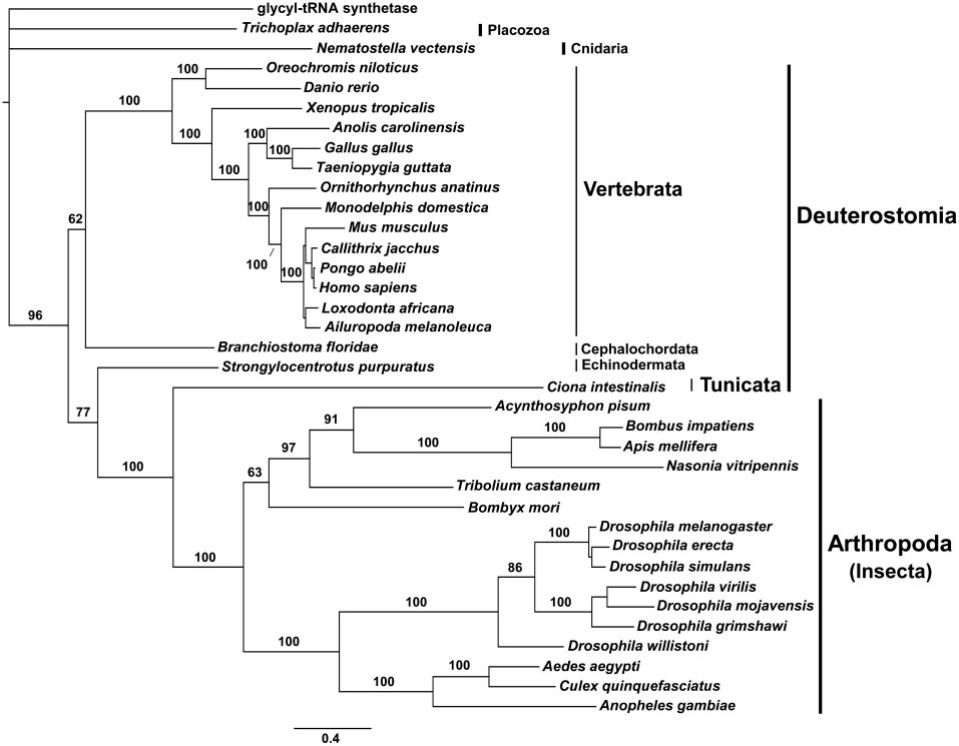
Bayesian phylogenetic inference for animal pol γ-β nucleotide sequences. The outgroup sequence used was the glycyl-tRNA synthetase from the bacterial species *Thermus thermophilus* (accession number AJ222643.1). The 50% majority-rule consensus tree was inferred using MrBayes 3.2, as described under Materials and Methods. Bayesian posterior probability values are indicated for almost all nodes. The scale bar indicates substitutions per site.

Bayesian phylogenetic analysis using pol γ-β nucleotide sequences (fig. 3) also shows that the insect proteins have evolved at a much higher rate than vertebrates. The rate of substitutions observed for the sequences from most Deuterostomia species again matches that for the sequences from basal animal groups, suggesting that pol γ-β also retained more ancestral characters for these groups. Moreover, the sequence from the tunicate *Ci. intestinalis* again grouped with those from Arthropoda, indicating a remarkable resemblance and their significant divergence from the ancestral accessory subunit polypeptide. We were unable to find any pol γ-β coding sequence from species of nematodes, as discussed below.

The fast evolutionary rates observed for the pol γ-α and -β genes in insects, tunicates, and nematodes appear to follow a general tendency described for the majority of nuclear-encoded genes from these groups (Li 1997; Mitreva et al. 2005; *Drosophila* 12 Genomes Consortium et al. 2007; Denoeud et al. 2010). Interestingly, our preliminary analyses of the genes encoding mtSSB and mtDNA helicase show substitution rates that differ from those of the pol γ-α and -β genes among animal groups (Oliveira MT, Haukka J, Kaguni LS, unpublished data). Thus, it appears that the mtSSB and mtDNA helicase genes may have atypical evolutionary constraints, but this requires further validation.

### New Motif in the Exonuclease Domain of Vertebrate pol γ-α: Implications for DNA Binding

**Fig. 4.—:**
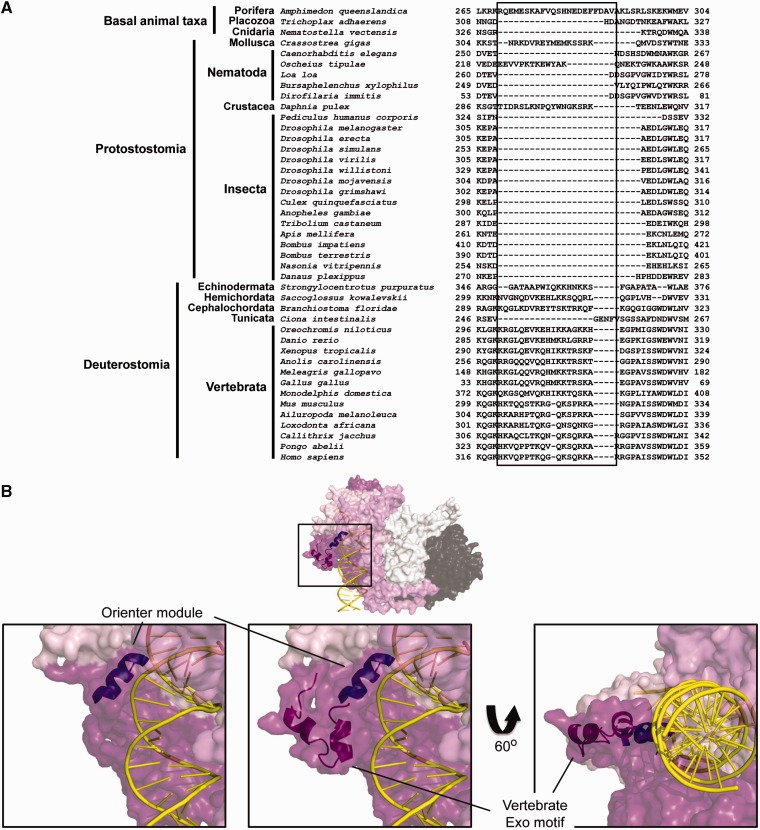
Identification of a new Exo motif in vertebrate pol γ-α, potentially implicated in primer–template DNA binding. (*A*) Amino acid sequence alignment indicates the presence of the extra residues (boxed) between pol γ-α motifs Exo II and III in all species of Vertebrata, and a few other animal groups, including other deuterostome species. These residues are disordered in the crystal structure of the human pol γ apo-holoenzyme (3IKM; Lee et al. 2009). (*B*) Structural model of the human residues indicated in (*A*) showing their proximity to the primer–template DNA binding cleft. The left panel shows the DNA binding cleft structure without the new vertebrate Exo motif, as it appears in the PDB data file 3IKM. Primer–template DNA binding to pol γ-α was modeled by Euro et al. (2011) and the orienter module (see text for details) is shown as described by Szczepanowska and Foury (2010). Colors are as in figure 1.

The MSA identified an insertion of approximately 17 amino acid residues between motifs Exo II and III of the pol γ-α exonuclease domain (H320–A336 in humans; A308–A309 in *D. melanogaster*) that is present in all species of Vertebrata, and possibly other Deuterostomia species (except *Ci. intestinalis*; fig. 4*A*). Similarly long regions in the same position are present in *Da. pulex* (Arthropoda: Crustacea), *Oscheius tipulae* (Nematoda: Rhabditida), *Cr. gigas* (Mollusca: Bivalvia), and *A. queenslandica* (Porifera: Demospongiae), but these have little sequence conservation and may represent independent insertion events into the pol γ-α genes of these species. The insertion is several residues downstream of the orienter, a structural module that is highly conserved within eukaryotes, and which has been reported to coordinate the balance between the polymerase and exonuclease functions (Szczepanowska and Foury 2010).

Locating the new vertebrate Exo motif in the crystal structure of the human pol γ-α (PDB: 3IKM; Lee et al. 2009) revealed that the insertion is part of a disordered region (K319–S344) for which we have no structural information, but which may indicate a flexible domain involved in transient interactions. Modeling the missing residues in the human pol γ-α structure based upon the secondary structure prediction algorithm built into the I-TASSER server resulted in two short alpha helices connected by a short loop, which orient this element toward the DNA binding cleft of the enzyme (fig. 4*B*). In particular, residues H320, K327, K331, and K335 are in close proximity to the minor groove of the primed DNA template modeled onto the putative DNA binding cleft of pol γ-α (Euro et al. 2011). We postulate that the new Exo motif may represent a module that serves to enhance DNA binding in vertebrate pol γs.

### New Motif in the IP Subdomain of Vertebrate pol γ-α: Implications for pol γ-β Interactions

**Fig. 5.—:**
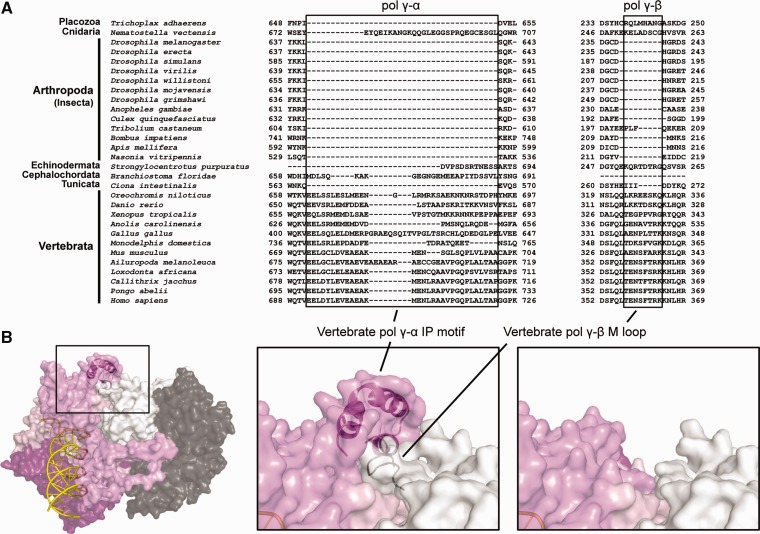
Identification of a new, putative interacting surface between the catalytic and accessory subunits of vertebrate pol γ. (*A*) Amino acid sequence alignments indicate the presence of new motifs in the pol γ-α IP subdomain (boxed, left panel) and in the pol γ-β middle domain (boxed, right panel) of all species of Vertebrata. Only the species for which both pol γ-α and -β sequences were retrieved are shown. The indicated residues in pol γ-α are disordered in the crystal structure of the human pol γ holoenzyme (3IKM; Lee et al. 2009); likewise those in pol γ-β are disordered in the crystal structure of the human pol γ-β dimer (2G4C; Fan et al. 2006). (*B*) Model of the human residues indicated in (*A*), suggesting that the predicted structural elements are in close proximity to each other. The right panel shows the possible interacting region in the absence of the newly identified motifs, as they appear in the PDB files 3IKM and 2G4C. Primer–template DNA binding to pol γ was modeled by Euro et al. (2011). Colors are as in figure 1.

The spacer region of pol γ-α, which connects the N-terminal exonuclease domain to the C-terminal polymerase domain, contains intrinsic processivity (IP) and AID subdomains (fig. 1). As their names suggest, these subdomains provide structural platforms for supporting both the intrinsic processivity of pol γ-α alone and the enhanced processivity of the holoenzyme, respectively (Lee et al. 2009). Our MSA also identified an insertion of 30 amino acid residues on average in the IP subdomain (E692–R722 in humans; L640–S641 in *D. melanogaster*) that is present consistently in all species of Vertebrata (fig. 5*A*). Other deuterostome species, such as *St. purpuratus* (Echinodermata) and *Branchiostoma floridae* (Cephalochordata), and some other noninsect animal species also have insertions of varying sizes in this position. However, the low sequence similarity does not provide enough evidence for homology among the new vertebrate IP motif and the regions to which it aligns in these other animals. In fact, the N-terminal region of this motif in vertebrates is highly conserved, but its C-terminus shows high variability. Again, the sequence from *Ci. intestinalis* resembles significantly those of insect species due to the lack of any amino acid residues in this region. In summary, this element is a distinct and conserved, derived feature in vertebrate pol γ-α; for other metazoan groups, there is no clear indication of its evolutionary constraints.

The new IP motif was localized in a region of the human pol γ-α crystal structure for which most of the residues were also disordered (G674–R709) (Lee et al. 2009). Although the C-terminus of the motif contains a residue mutated in some cases of human patients with Alpers disease (R722H) and possibly implicated in DNA binding (Euro et al. 2011), modeling the missing 36 amino acids resulted in a helix-turn-helix structure located in close proximity to a loop at the end of the Middle domain of the proximal pol γ-β protomer (fig. 5*B*). Part of this loop in the proximal pol γ-β, which to our knowledge has not been described previously and is hereafter called the “M loop” (T357–K364 in humans; D238–H239 in *D. melanogaster*), is again almost exclusive to vertebrates (fig. 5*A*), although Echinodermata (*St. purpuratus*), Cnidaria (*N. vectensis*), and Placozoa (*T. adhaerens*) do possess several residues in this same region. A firm correlation between the conserved new IP motif in pol γ-α and the M loop of pol γ-β occurs only for vertebrate species; the absence of both structural elements is firmly correlated only for insects and tunicates. Unfortunately, without a better taxa representation, we are unable to conclude whether both elements have been lost in insects and tunicates or gained in vertebrates.

### Dimerization of pol γ-β

**Fig. 6.—:**
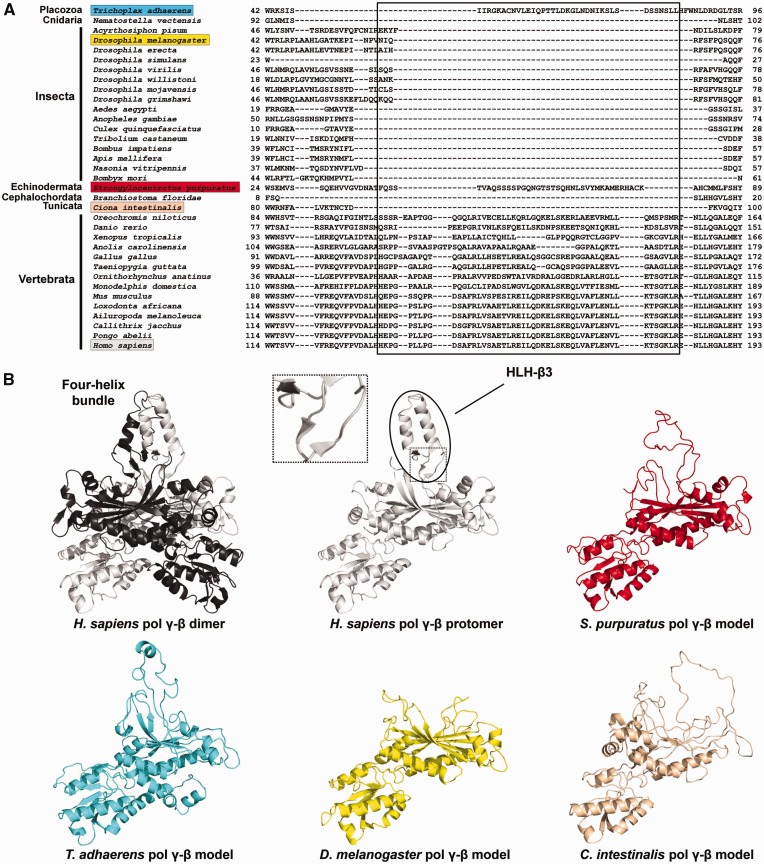
Dimerization of vertebrate pol γ-β through the formation of the four-helix bundle structure. (*A*) Amino acid sequence alignment indicates the presence of the HLH-3β domain (boxed) in all species of Vertebrata and possibly in few other animal groups. (*B*) Comparison of the crystal structure of the human pol γ-β dimer and structural models for pol γ-β of *Trichoplax adhaerens*, *Strongylocentrotus purpuratus*, *Drosophila melanogaster,* and *Ciona intestinalis*, showing that only vertebrate pol γ-β can fold into a HLH-3β structure and therefore form the four-helix bundle dimerization interface. The inset shows the three short β-sheets at the base of the HLH-3β structure. For more information about the structural features of the four-helix bundle fold, see Kamtekar and Hecht (1995) and Carrodeguas et al. (2001).

Structural and biochemical data have documented that the human and mouse pol γ-βs form homodimers in solution and that the homodimer form associates with pol γ-α to constitute a functional heterotrimeric holoenzyme (Carrodeguas et al. 2001; Fan et al. 2006; Yakubovskaya et al. 2006; Lee et al. 2009). The major pol γ-β dimerization interface is provided by the HLH-β3 [helix-loop-helix, 3 β-sheets] domain (H133–R182 in humans; N63–Q65 in *D. melanogaster*), which is present consistently across all vertebrate species (fig. 6*A*). Interestingly, the echinoderm *St. purpuratus* and the placozoan *T. adhaerens* also have amino acid residues that could potentially fold into a partial HLH-β3 structure. We tested this hypothesis by modeling the structure of pol γ-β from these two species and that from *D. melanogaster* and *Ci. intestinalis* (control species that lack completely the HLH-β3 element). Neither the helix-loop-helix structure, responsible for the formation of the four-helix bundle with the adjacent pol γ-β, nor the three β sheets found at the base of the four-helix bundle are clearly observed for *St. purpuratus* and *T. adhaerens* (fig. 6*B*). As expected, the pol γ-β models for *D. melanogaster* and *Ci. intestinalis* also have none of the structural elements necessary for the HLH-β3 folding. All these proteins are, therefore, most likely unable to homodimerize using the same structural features adopted by the vertebrate pol γ-β. We propose here that the ability to form homodimers is exclusive to vertebrate pol γ-β, and that this feature appears to have emerged through the acquisition of a structural evolutionary novelty, the HLH-β3 domain.

### Deletion of pol γ-α AID Subdomain and Absence of the pol γ-β Gene in Nematoda

**Fig. 7.—:**
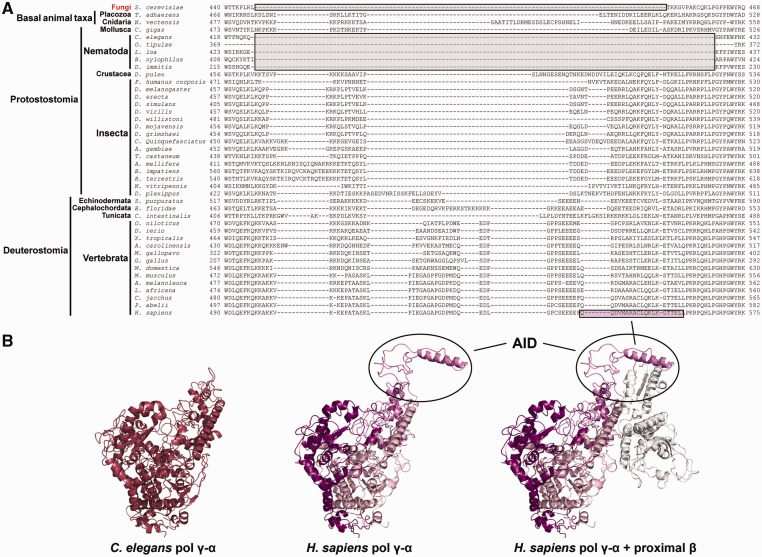
The likely monomeric nature of nematode pol γ. (*A*) Amino acid sequence alignment indicates the absence of the pol γ-α AID subdomain (gray boxes) in Nematoda species, which is similar to the well-characterized enzyme from the yeast *Saccharomyces cerevisiae*. The pink box highlights the residues forming the L helix of human pol γ-α, the major point of interaction with the proximal pol γ-β. (*B*) Structural model of the *Caenorhabditis elegans* pol γ-α (left panel) adjacent to the human pol γ structure (3IKM; Lee et al. 2009) that contains a fully folded AID subdomain (middle and right panels). Colors are as in figure 1.

Our MSA also shows clearly that the residues of pol γ-α AID subdomain are completely absent in all species of Nematoda (fig. 7). In addition, we searched exhaustively for homologs of pol γ-β in diverse sequence databanks, but found only nematode sequences related to aminoacyl-tRNA synthetase genes, with very high *E* value scores (data not shown). Others have indicated previously, using an alignment with a small number of amino acid sequences, that the *C. elegans* pol γ-α may not have the AID subdomain and might function as a monomeric enzyme (Bratic et al. 2009; Addo et al. 2010); our bioinformatic analyses provide support for this claim, and suggest that this is a common feature for all nematode species.

## Discussion

Although the fields of animal mitochondrial genomics and mtDNA replication have expanded greatly in recent years, insights from these two fields have not yet effectively cross-fertilized our understanding of mitochondrial genome biology. Complete mitochondrial genomes of virtually all animal groups (reviewed in Gissi et al. 2008 and D’Onorio de Meo et al. 2012) and thousands of human individuals (reviewed in Ye et al. 2014) are currently available, showing strong constraints such as the small size of the molecule (∼16 kb), its circular conformation and compact gene organization (∼37 genes and few noncoding nucleotides), the strictly orthologous encoded genes, and the very low importance of recombination sensu stricto for the purpose of genome inheritance (supplementary fig. S3 and table S2, Supplementary Material online). On the other hand, research has also shown a great variability in nucleotide and amino acid substitution rates, gene rearrangement, number and length of noncoding regions, and genetic codes, whose underlying mechanisms are related generally to mtDNA transaction processes, such as replication, transcription, and repair. Testing the functional importance of such changes has been difficult due to our inability to perform mtDNA site-directed mutagenesis (reviewed in Moraes et al. 2014); at the same time, this experimental constraint does not apply to the study of mitochondrial proteins encoded in the nucleus, as are all of the proteins involved in mtDNA metabolism.

In one of the first attempts to correlate genomic structural diversity and functional mechanisms, Jiang et al. predicted, based on the variability of nucleotide composition bias along the mitochondrial genome of snakes, that their duplicated major noncoding regions (D-loops) may contain an additional origin of heavy-strand replication, implying a different replication mode (see vertebrate mtDNA replication mechanisms below) (Jiang et al. 2007). *Cis*-elements that control transcription and replication are most likely present in the mtDNA noncoding regions, and hence their genomic rearrangements would reflect more readily mechanistic differences in these fundamental processes. However, a number of important functional sites have been described within the mtDNA coding region, such as the binding sites for transcription termination factors, which in fact have a documented impact on both transcription and replication, regulating/restricting the progression of mtDNA replication forks to prevent collisions and to facilitate productive interaction between the two machineries (Hyvarinen et al. 2007, 2011; Joers et al. 2013). Therefore, evolutionary changes involving only gene rearrangements of the mitochondrial genome are also likely to correlate with changes in maintenance mechanisms. To develop molecular insights into DNA polymerase structure and function that could impact genome evolution, we have reported here the sequence and structural evolution of the key enzyme in animal mtDNA replication, pol γ, establishing correlations and evaluating their implications at the biochemical/molecular level, in consideration of the features of the mitochondrial genome in different metazoan groups.

**Fig. 8.—:**
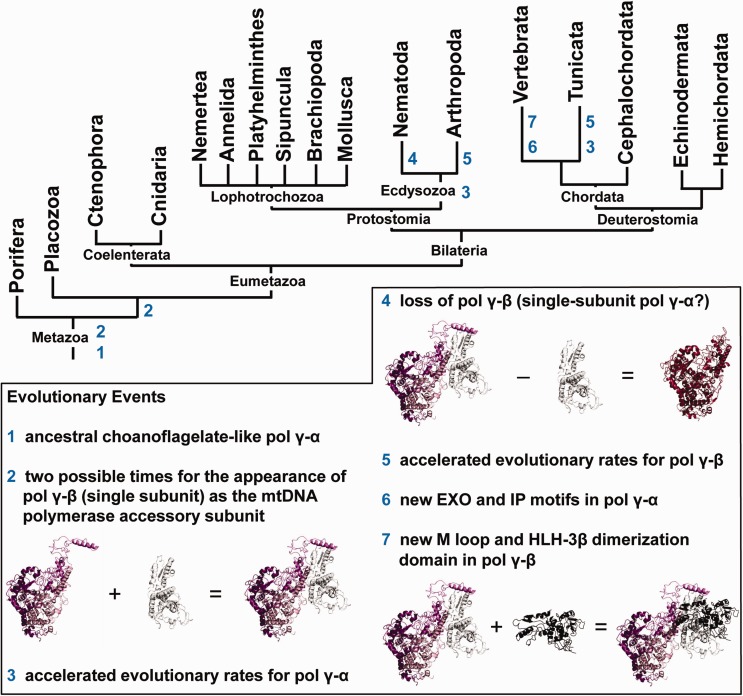
Phylogeny of animal groups indicating the molecular evolutionary events identified in this work for the catalytic and accessory subunits of the mitochondrial replicase. The tree is based on that in Philippe et al. (2009).

Analyses of amino acid sequence alignments, Bayesian phylogenetic inferences, and protein modeling allowed us to identify three important evolutionary patterns within the catalytic and accessory subunits of animal pol γ (fig. 8). First, it appears that vertebrate pol γs show a high level of constraint, with little sequence and structural variability within the group. Both subunits have evolved in a highly similar fashion (figs. 2 and 3) that is distinct from that of other animal groups, and together have acquired at least four structural evolutionary novelties: New Exo and IP motifs in pol γ-α (figs. 4 and 5), and a new M loop and the HLH-3β domain in pol γ-β (figs. 5 and 6). The new motif in the Exo domain is potentially present in other deuterostome species, such as echinoderms, hemichordates, and cephalochordates, but it is clearly absent in tunicates (fig. 4*A*). The amino acid residues involved have the potential to fold into a structure that may enhance and/or stabilize enzyme–DNA interactions (fig. 4*B*). Interestingly, these amino acids appear disordered in the crystal structure of the human pol γ apo-holoenzyme (Lee et al. 2009), indicating that the motif may adopt a flexible structure that might be stabilized upon primer–template DNA binding. In addition to a proposed role in DNA binding per se, the new motif may function specifically in concert with the conserved element known as the orienter (Szczepanowska and Foury 2010). The orienter lies only three amino acids upstream and within 8.3 Å of the N-terminus of the new motif in the three-dimensional structure (fig. 4*B*), and has been shown to coordinate the exonuclease and polymerase functions of pol γ to enhance the fidelity of the holoenzyme. Thus, the close proximity of the new motif in the Exo domain and its likely capacity to bind DNA suggests that it may enhance and/or regulate the function of the orienter. The acquisition of the new motif in the IP subdomain of vertebrate pol γ-α is correlated with the acquisition of the M loop in the middle region of vertebrate pol γ-β (fig. 5). These structures may interact, thereby increasing the contact surface between the catalytic and the proximal accessory subunit. However, this putative enhanced interaction may only be stable either upon binding to DNA or to another component of the mtDNA replisome, given that the residues in both subunits are disordered in the crystal structures of the human apo-holoenzyme and in the pol γ-β dimer, respectively (Fan et al. 2006; Lee et al. 2009). We believe that these new structural elements found in pol γ-α and -β of vertebrates are not only remarkable from an evolutionary point of view but also important from a biomedical perspective, as they lie within the structural and functional regions defined as clusters 2 and 5 by Euro et al., which bear diverse pathogenic mutations of the human pol γ-α (Euro et al. 2011; Farnum et al. 2014), or in close proximity to the disease mutation R369G in human pol γ-β (Young et al. 2011; Craig et al. 2012). Future biochemical study of each of these three new elements is warranted to test our predictions, and to determine their specific roles in both vertebrate pol γ function and mtDNA replication.

Perhaps the most striking result regarding the evolution of vertebrate pol γ is the homodimeric nature of pol γ-β in this animal group, which we propose to have arisen with the acquisition of the HLH-3β domain (fig. 6). The two alpha-helices within this domain in one pol γ-β protomer interact with those in the other protomer to form a four-helix bundle, a common protein fold that is characterized by four helices packed against each other, in a roughly lengthwise configuration, at a specific angle relative to a virtual central axis (Kamtekar and Hecht 1995; Carrodeguas et al. 2001). Residues from echinoderm and placozoan species, aligned with the vertebrate HLH-3β domain, are clearly nonhomologous, having evolved independently and unable to fold into a similar structure (fig. 6). We attempted to identify the origin of the vertebrate dimerization domain in BLAST searches of its sequence against the human, mouse, and zebrafish genomes, using relaxed parameters, but only the pol γ-β gene sequences themselves were obtained as results (data not shown). Previously published alignments (Fan et al. 2006; Lee et al. 2010) using the amino acid sequences of only a few animal pol γ-βs (human, mouse, and *D. melanogaster*) mistakenly suggested that two insertions into mammalian pol γ-β created the dimer interface. Our analysis using the highest number of animal pol γ-β sequences available to date (35 sequences vs. the commonly used alignment of the human, mouse, frog, and fly sequences [Carrodeguas et al. 2001; Fan et al. 2006; Lee et al. 2009]) argues clearly that the residues comprising the HLH-3β domain were inserted as a whole, early in the evolution of Vertebrata.

Deletion variants of the human and mouse pol γ-β that lack most of the HLH-3β domain (called ΔI4, and comprising residues R147–G179 in humans) are observed as monomers in solution, though the proteins crystallize as dimers (Carrodeguas et al. 2001; Lee et al. 2009, 2010). Using this pol γ-β variant and related proteins, it has been shown that the functions of the two pol γ-β protomers that constitute the β dimer in the human holoenzyme are distinct: The proximal subunit strengthens primer–template DNA interactions whereas the distal one accelerates the DNA synthetic rate (Lee et al. 2010). Although the authors argue that the homodimeric nature of human pol γ-β provides the molecular basis for these functions of the accessory subunit, our earlier biochemical data show at least in the fly that the monomeric *D. melanogaster* pol γ-β imparts the same functions to the cognate fly holoenzyme (Lewis et al. 1996; Wang et al. 1997). Thus, at least in invertebrates, dimerization of pol γ-β is not a structural requirement for enhanced DNA binding, or the increased processivity and rate of nucleotide polymerization of the pol γ holoenzyme. If the additional pol γ-β protomer indeed provides increased activity to the vertebrate holoenzyme, its effect is likely mitigated by the fact that the stimulation of the human pol γ by its cognate mtSSB is only moderate (∼3-fold), and is observed only at low salt concentrations in vitro (Oliveira and Kaguni 2010). In contrast, *D. melanogaster* pol γ is stimulated up to 20-fold by its cognate mtSSB, and this is observed over a broad range of KCl concentrations (Farr et al. 1999). In this regard, we would speculate that the functions of the distal pol γ-β protomer in the vertebrate mtDNA replisome are likely at least in part contributed by mtSSB in insect (invertebrate) systems, either directly by protein–protein interactions, or by its ability to organize the DNA substrate.

An interesting question that has been raised previously (Lee et al. 2010) is whether the homodimeric state of vertebrate pol γ-β is a basal or a derived character, taking into account that class II aminoacyl-tRNA synthetases also function as homodimers. Our data argue that the pol γ-β from all invertebrate species is monomeric (except in nematodes, which have lost the gene), including basal animal species, and that this is an ancestral character for metazoans. Thus, we propose that the dimerization of modern class II aminoacyl-tRNA synthetases evolved independently from that of vertebrate pol γ-β but perhaps for a common reason, to allow more sophisticated mechanistic or regulatory strategies during protein translation and mtDNA replication, respectively. This is supported further by the fact that the sequence and structure of the dimerization domain of the *Th. thermophilus* glycyl-tRNA synthetase (Logan et al. 1995; Arnez et al. 1999), a prototype of prokaryotic class II aminoacyl-tRNA synthetases, is significantly dissimilar to the HLH-3β domain of vertebrate pol γ-β (Carrodeguas et al. 2001; Fan et al. 2006).

The shared sequence and structural features described above for vertebrate pol γ, absent in other metazoans, correlate with the fact that vertebrate mitochondrial genomes are extremely invariable in genome architecture, gene content and strand asymmetry, and in the position and number of noncoding regions (supplementary fig. S3 and table S2, Supplementary Material online; reviewed in Gissi et al. 2008). We hypothesize that the correlation between stabilization of mitochondrial genomic features and shared pol γ structure and sequence evolution may reflect a common mechanism of mtDNA replication in vertebrates. In this regard, it is tempting to speculate that the dimerization of the vertebrate accessory subunit of pol γ is related to its acquired ability to bind to double-stranded DNA: Carrodeguas et al. (2002) showed that the human pol γ-β dimer binds specifically to template–primer junctions in vitro and on the basis of physiological experiments, Di Re et al. (2009) have proposed that it serves a role in regulation of nucleoid structure. Holt, Jacobs and collaborators have shown that the mtDNA of human tissues and cells in culture, mouse, and chicken (*Gallus gallus*) are replicated in a similar fashion through one (or two) mechanism(s) that involve(s) the incorporation of extensive tracts of RNA throughout the mtDNA lagging strand (Yang et al. 2002; Yasukawa et al. 2006; Pohjoismaki et al. 2010). This RNA appears to originate from processed transcripts of mitochondrial genes that hybridize to the displaced strand during synthesis of the leading DNA strand, and the authors propose that these transcripts later serve as primers for lagging strand synthesis (Reyes et al. 2013). There is also evidence that at least some fraction of the replicating mtDNA molecules are replicated by a coupled leading and lagging strand synthesis mechanism, resembling that of bacterial species in the replication of their circular chromosomes (Holt et al. 2000; Reyes et al. 2005). Although longstanding alternate mechanisms exist (reviewed in Bogenhagen and Clayton 2003), there has always been a consensus that the mode(s) of mtDNA replication is (are) shared among vertebrate species, and our data corroborate this idea.

The second evolutionary pattern we have identified among the animal pol γ subunits is that both the pol γ-α and -β genes of arthropods and tunicates have evolved approximately twice as rapidly as their vertebrate homologs (figs. 2 and 3), despite the fact that the polypeptides they encode tend to be shorter (supplementary figs. S1 and S2, Supplementary Material online). In addition to the phylogenetic clustering of arthropod and tunicate sequences, *Ci. intestinalis* pol γ-α and -β have some structural features that are more similar to arthropods than to other deuterostome species: The complete absence of the new Exo and IP motifs in pol γ-α and of the M loop and the HLH-3β domain in pol γ-β. Even though these are clearly vertebrate elements, echinoderms do possess residues that align with the new Exo and IP motifs, the M loop and the HLH-3β domain, cephalochordates may also form the new Exo and IP motifs, and hemichordates may possess the new Exo motif. We investigated whether the *Ci. intestinalis* sequences might result from a contamination of an arthropod DNA that was mistakenly assigned to this tunicate, or a product of a possible lateral gene transfer. Serving to exclude the possibility of contamination, we found that the sequences of both genes were firmly assembled into chromosomes 12 and 7, respectively (data not shown), and not randomly annotated in an unplaced contig/scaffold. The pol γ-β gene architecture in the *Ci. intestinalis* genome, for example, is highly similar to that in the human, mouse, chicken, and zebrafish genomes (eight exons with conserved exon/intron boundaries), whereas the homologous gene of *Apis mellifera* and *Drosophila* species has only two exons, with variable exon/intron boundaries (data not shown), indicating that the probability of lateral transfer of an arthropod gene is minimal. Thus, the sequences of *Ci. intestinalis* used in this report are indeed encoded in its genome, and most likely cluster with those of arthropods as a consequence of the phenomenon of long branch attraction (reviewed in Bergsten 2005).

Tunicate mtDNAs exhibit a very high degree of genome variability, which includes frequent protein-coding gene rearrangements, extra copies of tRNA genes, maximal gene strand asymmetry and extremely short noncoding regions (supplementary fig. S3 and table S2, Supplementary Material online; reviewed in Gissi et al. 2008). Among Deuterostomia, this is the taxon that may be the most likely to employ a substantially different mode of replicating its mitochondrial genome; in addition to the features of the mtDNA itself, the monomeric nature of pol γ-β and the fast rate of evolutionary change for the catalytic and accessory subunit genes all serve as indicators. However, experimental data to validate this hypothesis are needed. For arthropods, on the other hand, a substantial amount of research toward the understanding of mtDNA replication is available, using *D. melanogaster* as a model system (reviewed in Oliveira et al. 2010). Although this species may not be representative of the entire diverse group of Arthropoda, considering that its mitochondrial genome (Lewis et al. 1995), and that of other dipterans, shares features with basal species of the phylum, such as the horseshoe crab *Limulus polyphemus* (Chelicerata: Xiphosura) (Lavrov et al. 2000) and Amblypygi spiders (Chelicerata: Arachnida) (Fahrein et al. 2009), one might expect that studies with *D. melanogaster* may reveal a conserved and basal mechanistic model of mtDNA replication. Joers and Jacobs (2013) have described recently that *D. melanogaster* mtDNA is replicated predominately through a strand-coupled DNA synthesis mode, initiating within the major noncoding region and proceeding unidirectionally at an irregular rate of elongation. Unlike the situation in vertebrates, the presence of RNA molecules hybridized to the mtDNA lagging strand was not detected, although DNA replication intermediates containing short stretches of single-stranded DNA was observed for a small subset of the replicating mtDNA molecules. In combination with our findings on the rapid evolution of the arthropod pol γ subunits and the heterodimeric composition of the holoenzyme, the data suggest that arthropods may employ a unique mode of replication among metazoan mtDNAs. Considering that the mtDNA features among arthropod species are also highly diverse (supplementary fig. S3 and table S2, Supplementary Material online), with groups such as the myriapods and the Acari arachnids showing numerous rearrangements of gene order (reviewed in Gissi et al. 2008), we can also predict that additional mechanisms of replication will be identified.

Remarkably, as the third pattern of animal pol γ evolution, we found that the oligomeric form of the holoenzyme is more variable than the concept of the monomeric versus homodimeric nature of pol γ-β. Our data argue that pol γ-β is not found in any nematode species, and that this is a derived character: First, we retrieved no gene coding for a homologous protein, despite exhaustive databank searches and second, we observed that nematode pol γ-α lacks the residues needed to form the AID subdomain, the structural platform for the interactions between the catalytic and the accessory subunits in other animal species (fig. 7). Although it may be premature to conclude that nematode pol γ-α is a monomeric enzyme, we suggest that the mtDNA replisome in this animal group is distinct from that of any other metazoan taxa, and it might employ a distinct mtDNA replication mechanism. The facts that nematode pol γ-αs are the fastest evolving sequences among metazoans (fig. 2), and the mtDNA of Nematoda species has unusual features, such as loss of the *atp8* gene, all genes coded only in one strand, and frequent changes in the gene order among species (supplementary fig. S3 and table S2, Supplementary Material online; reviewed in Gissi et al. 2008), are all consistent with this speculation. Indeed, Lewis et al. (2015) have reported that mtDNA replication in the nematode *C. elegans* most likely proceeds via a rolling circle mechanism, which differs from any other mechanism documented in metazoans to date. Alternatively, one might argue that this monomeric pol γ-α is not the processive, replicative DNA polymerase of nematode mitochondria because the gene is not essential for *C. elegans* development and survival (Bratic et al. 2009; Addo et al. 2010); it could be a mtDNA repair enzyme that does not require processive DNA synthesis, therefore eliminating the need for pol γ-β (Longley et al. 1998; Copeland and Longley 2014). However, it is important to point out that the yeast *S. cerevisiae* pol γ is a processive enzyme that does not contain a pol γ-β-like accessory subunit (Foury 1989). Furthermore, the knockdown of pol γ-α in *C. elegans* does cause strong mtDNA depletion as in *D. melanogaster*, mouse, and humans (Iyengar et al. 1999, 2002; Spelbrink et al. 2000; Hance et al. 2005; Fukuoh et al. 2014). In our view, the pol γ-α sequences we retrieved for nematodes do represent the mitochondrial replicase, and the successful development of *C. elegans* with a substantially lower level of both pol γ-α and mtDNA most likely indicates that mitochondrial oxidative phosphorylation is the feature dispensable for egg and larval viability in this organism.

Although we attempted to correct for the bias in taxa sampling by excluding several species of *Drosophila* and mammals from our data set, a large overrepresentation of sequences from Insecta and Vertebrata remain, limiting the interpretation of our results for other animal groups. Nonetheless, we consider the correlations presented here regarding pol γ evolution as robust, and in all likelihood are a reflection of distinct mechanistic differences in mtDNA replication. Because of its roles in mitochondrial function, pol γ has been studied extensively from a clinical and biomedical perspective. Our study argues that revealing its interesting evolutionary path(s) among metazoans, which has just recently caught the attention of biochemists and structural biologists, is not only of interest per se, but may also serve to promote the understanding of human disease-related phenotypes caused by pol γ-α and -β mutations. Moreover, investigating whether or not the evolutionary paths of the mtDNA helicase and mtSSB throughout metazoan history are consistent with those of pol γ might reveal coevolution toward a more efficient replisome. For example, we have demonstrated recently that the *D. melanogaster* mtDNA helicase contains an iron–sulfur cluster in its N-terminal domain, which appears to be absent in the human homolog (Stiban et al. 2014). What its functions and phylogenetic distribution are, and how this property has evolved represent important new questions. Clearly, molecular evolutionary studies of each of the protein components of the animal mtDNA replisome are warranted to achieve a better understanding both of organelle evolution and of the processes leading to mitochondrial dysfunction.

## Supplementary Material

Supplementary tables S1 and S2 and figures S1–S3 are available at *Genome Biology and Evolution* online (http://www.gbe.oxfordjournals.org/).

Supplementary Data
