# Supplementary material for: Evolution of the Metazoan Mitochondrial Replicase
Source: Genome Biol Evol. 2015 Mar 3;7(4):943–59. doi: 10.1093/gbe/evv042 (PMC4419789; doi:10.1093/gbe/evv042)
Supplement: Supplementary Data [file supp_7_4_943__index.html]

Evolution of the Metazoan Mitochondrial Replicase — Supplementary Data 

# Evolution of the Metazoan Mitochondrial Replicase

## Supplementary Data

files

**Files in this Data Supplement:**

- Supplementary Data - pdf file
