# Supplementary material for: Evolution of the Metazoan Mitochondrial Replicase
Source: Genome Biol Evol. 2015 Mar 3;7(4):943–59. doi: 10.1093/gbe/evv042 (PMC4419789; doi:10.1093/gbe/evv042)
Supplement: Supplementary Data [file supp_evv042_Supp_material_composite.pdf]

Supplemental Table

Table S1. Total non-redundant sequences for animal pol γ-α and β retrieved from diverse public databases.

Species	Common name	Phylum	Class	Order	Accession Number
Pol γ-α					
Amphimedon queenslandica	sponge	Porifera	Demospongiae	Haplosclerida	XM_003389221.1
Trichoplax adhaerens	placozoan	Placozoa	Tricoplacia	-	XM_002116311.1
Nematostella vectensis	starlet sea anemone	Cnidaria	Anthozoa	Actiniaria	scaffold_62
Hydra magnipapillata	fresh water polyp	Cnidaria	Hydrazoa	Hydroida	XP_002166917.2
Crassostrea gigas	pacific oyster	Mollusca	Bivalvia	Ostreoida	EKC38630.1
Caenorhabditis elegans	roundworm	Nematoda	Chromadorea	Rhabditida	NM_064191.1
Bursaphelenchus xylophilus	pine wood nematode	Nematoda	Chromadorea	Tylenchida	scaffold01109
Oscheius tipulae	hermaphroditic soil nematode	Nematoda	Chromadorea	Rhabditida	959 Nematode Genomes
Dirofilaria immitis	heartworm	Nematoda	Chromadorea	Spirurida	959 Nematode Genomes RNA-seq
Loa loa	eye worm	Nematoda	Chromadorea	Spirurida	XM_003141747
Daphnia pulex	water flea	Arthropoda	Crustacea	Diplostraca	EFX73911.1
Pediculus humanus corporis	human body louse	Arthropoda	Insecta	Phthiraptera	XM_002425672.1
Drosophila melanogaster	fruitfly	Arthropoda	Insecta	Diptera	NM_057473.3
Drosophila sechellia	fruitfly	Arthropoda	Insecta	Diptera	XM_002035723.1
Drosophila yakuba	fruitfly	Arthropoda	Insecta	Diptera	XM_002088608.1
Drosophila erecta	fruitfly	Arthropoda	Insecta	Diptera	XM_001969538.1
Drosophila simulans	fruitfly	Arthropoda	Insecta	Diptera	XM_002079353.1
Drosophila persimilis	fruitfly	Arthropoda	Insecta	Diptera	XM_002023169.1
Drosophila pseudoobscura pseudoobscura	fruitfly	Arthropoda	Insecta	Diptera	XM_001356205.2
Drosophila ananassae	fruitfly	Arthropoda	Insecta	Diptera	XM_001962747.1
Drosophila willistoni	fruitfly	Arthropoda	Insecta	Diptera	XM_002066715.1

Drosophila grimshawi	fruitfly	Arthropoda	Insecta	Diptera	XM_001988310.1
Drosophila virilis	fruitfly	Arthropoda	Insecta	Diptera	XM_002057431.1
Drosophila mojavensis	fruitfly	Arthropoda	Insecta	Diptera	XM_002004015.1
Aedes aegypti	yellow fever mosquito	Arthropoda	Insecta	Diptera	XM_001647501.1
Culex quinquefasciatus	Southern house mosquito	Arthropoda	Insecta	Diptera	XM_001862579.1
Anopheles gambiae	mosquito	Arthropoda	Insecta	Diptera	XM_311006.5
Tribolium castaneum	Red flour beetle	Arthropoda	Insecta	Diptera	XM_963795.2
Apis mellifera	Honey bee	Arthropoda	Insecta	Hymenoptera	XM_395230.4
Bombus impatiens	common eastern bumble bee	Arthropoda	Insecta	Hymenoptera	XM_003490636.1
Bombus terrestris	buff-tailed bumblebee	Arthropoda	Insecta	Hymenoptera	XM_003393733.1
Nasonia vitripennis	jewel wasp	Arthropoda	Insecta	Hymenoptera	XM_001602152.2
Danaus plexippus	monarch butterfly	Arthropoda	Insecta	Lepidoptera	EHJ64805.1
Branchiostoma floridae	Florida lancelet	Chordata	Cephalochordata/ Leptocardii	Amphioxiformes	XM_002601541.1
Strongylocentrotus purpuratus	purple sea urchin	Echinodermata	Echinoidea	Echinoida	XM_003726345.1
Paracentrotus lividus	common urchin	Echinodermata	Echinoidea	Camarodonta	Contig987_March08, sequence was extracted from the continuous alignment with S. purpuratus POLG1
Ciona intestinalis	vase tunicate	Chordata	Ascidiacea	Enterogona	XM_002601541.1
Oreochromis niloticus	Nile tilapia	Chordata	Actinopterygii	Perciformes	XM_003440509.1
Danio rerio	zebrafish	Chordata	Actinopterygii	Cypriniformes	XM_001921095.2
Xenopus tropicalis	western clawed frog	Chordata	Amphibia	Anura	XM_002932235.1
Xenopus laevis	African clawed frog	Chordata	Amphibia	Anura	NM_001087995.1
Anolis carolinensis	green anole	Chordata	Reptilia	Squamata	XM_003227051.1
Mus musculus	house mouse	Chordata	Mammalia	Rodentia	AK133584.1
Rattus norvegicus	Norway rat	Chordata	Mammalia	Rodentia	NM_053528.1
Cricetulus griseus	Chinese hamster	Chordata	Mammalia	Rodentia	XM_003512023.1
Monodelphis domestica	gray short tailed opossum	Chordata	Mammalia	Didelphimorphia	XM_003339979.1

Cavia porcellus	domestic guinea pig	Chordata	Mammalia	Rodentia	XM_003475248.1
Equus caballus	horse	Chordata	Mammalia	Perissodactyla	XM_001503047.1
Bos taurus	cattle	Chordata	Mammalia	Artiodactyla	XM_002702596.1
Sus scrofa	pig	Chordata	Mammalia	Artiodactyla	XM_001927064.2
Ailuropoda melanoleuca	giant panda	Chordata	Mammalia	Carnivora	XM_002919566.1
Canis lupus familiaris	dog	Chordata	Mammalia	Carnivora	XR_139923.1
Loxodonta africana	African savanna elephant	Chordata	Mammalia	Proboscidea	XM_003413892.1
Callithrix jacchus	white tufted ear marmoset	Chordata	Mammalia	Primates	XM_002749138.1
Macaca mulatta	rhesus monkey	Chordata	Mammalia	Primates	XM_001092360.2
Nomascus leucogenys	Northern white cheeked gibbon	Chordata	Mammalia	Primates	XM_003268488.1
Pongo abelii	Sumatran orangutan	Chordata	Mammalia	Primates	XM_002825804.1
Pan troglodytes	chimpanzee	Chordata	Mammalia	Primates	XM_523149.3
Homo sapiens	human	Chordata	Mammalia	Primates	NM_001126131.1
Gallus gallus	chicken	Chordata	Aves	Galliformes	XM_425079.3
Meleagris gallopavo	turkey	Chordata	Aves	Galliformes	XR_118363.1
Taeniopygia guttata	zebra finch	Chordata	Aves	Passeriformes	XR_054793.1
Saccoglossus kowalevskii	acorn worm	Hemichordata	Enteropneusta	-	XM_002733822.1
Pol γ-β					
Trichoplax adhaerens	placozoan	Placozoa	Tricoplacia	-	XM_002116806.1
Nematostella vectensis	starlet sea anemone	Cnidaria	Anthozoa	Actiniaria	XM_001622808.1
Crassostrea gigas	Pacific oyster	Mollusca	Bivalvia	Ostreoida	EKC19709.1
Ixodes scapularis	black-legged tick	Arthropoda	Acari	Ixodida	XM_002402635.1
Daphnia pulex	water flea	Arthropoda	Crustacea	Diplostraca	EFX64478.1
Drosophila melanogaster	fruitfly	Arthropoda	Insecta	Diptera	FJ635829.1
Drosophila erecta	fruitfly	Arthropoda	Insecta	Diptera	XM_001969535.1
Drosophila simulans	fruitfly	Arthropoda	Insecta	Diptera	XM_002079356.1
Drosophila virilis	fruitfly	Arthropoda	Insecta	Diptera	XM_002057434.1
Drosophila willistoni	fruitfly	Arthropoda	Insecta	Diptera	XM_002064759.1
Drosophila mojavensis	fruitfly	Arthropoda	Insecta	Diptera	XM_002002440.1

Drosophila grimshawi	fruitfly	Arthropoda	Insecta	Diptera	XM_001988313.1
Drosophila persimilis	fruitfly	Arthropoda	Insecta	Diptera	XM_002023176.1
Drosophila pseudoobscura	fruitfly	Arthropoda	Insecta	Diptera	XM_001356202.2
Drosophila ananassae	fruitfly	Arthropoda	Insecta	Diptera	XM_001962744.1
Aedes aegypti	yellow fever mosquito	Arthropoda	Insecta	Diptera	XM_001655250.1
Anopheles gambiae	mosquito	Arthropoda	Insecta	Diptera	XM_560198.4
Culex quinquefasciatus	Southern house mosquito	Arthropoda	Insecta	Diptera	XM_001869204.1
Tribolium castaneum	red flour beetle	Arthropoda	Insecta	Coleoptera	XM_001808620.1
Acyrthosiphon pisum	pea aphid	Arthropoda	Insecta	Hemiptera	XM_001942879.2
Bombus impatiens	common eastern bumble bee	Arthropoda	Insecta	Hymenoptera	XM_003487102.1
Bombus terrestris	buff-tailed bumblebee	Arthropoda	Insecta	Hymenoptera	XM_003398583.1
Apis mellifera	honey bee	Arthropoda	Insecta	Hymenoptera	XM_003250402.1
Nasonia vitripennis	jewel wasp	Arthropoda	Insecta	Hymenoptera	XM_001604448.2
Bombyx mori	domestic silkworm	Arthropoda	Insecta	Lepidoptera	AK385430.1 Whole gene
Ciona intestinalis	vase tunicate	Chordata	Ascidiacea	Enterogona	XM_002128585.1
Branchiostoma floridae	Florida lancelet	Chordata	Cephalochordata/ Leptocardii	Amphioxiformes	XM_002603575.1
Strongylocentrotus purpuratus	purple sea urchin	Echinodermata	Echinoidea	Echinoida	XM_785363.2
Oreochromis niloticus	Nile tilapia	Chordata	Actinopterygii	Perciformes	XM_003447969.1
Danio rerio	zebrafish	Chordata	Actinopterygii	Cypriniformes	XM_001922324.3
Xenopus tropicalis	western clawed frog	Chordata	Amphibia	Anura	XM_002942392.1
Anolis carolinensis	green anole	Chordata	Reptilia	Squamata	XM_003224433.1
Ornithorhynchus anatinus	platypus	Chordata	Mammalia	Monotremata	XM_001510754.2
Ailuropoda melanoleuca	giant panda	Chordata	Mammalia	Carnivora	XM_002924508.1
Canis lupus familiaris	dog	Chordata	Mammalia	Carnivora	XM_548032.3
Equus caballus	horse	Chordata	Mammalia	Perissodactyla	XM_001495189.2
Loxodonta africana	African savanna elephant	Chordata	Mammalia	Proboscidea	XM_003417305.1
Bos taurus	cattle	Chordata	Mammalia	Artiodactyla	NM_001075191.1

Delphinidae	dolphin	Chordata	Mammalia	Cetacea	ENSTTRG00000008785 (Ensembl)
Cavia porcellus	domestic guinea pig	Chordata	Mammalia	Rodentia	XM_003465931.1
Mus musculus	house mouse	Chordata	Mammalia	Rodentia	BC119131.1
Rattus norvegicus	Norway rat	Chordata	Mammalia	Rodentia	NM_001107060.1
Cricetulus griseus	Chinese hamster	Chordata	Mammalia	Rodentia	XM_003501876.1
Monodelphis domestica	gray short tailed opossum	Chordata	Mammalia	Didelphimorphia	XM_001379307.2
Nomascus leucogenys	Northern whitecheeked gibbon	Chordata	Mammalia	Primates	XM_003262632.1
Callithrix jacchus	whitetuftedear marmoset	Chordata	Mammalia	Primates	XM_002748152.1
Pongo abelii	Sumatran orangutan	Chordata	Mammalia	Primates	XM_002827729.1
Pan troglodytes	chimpanzee	Chordata	Mammalia	Primates	XM_001162210.2
Homo sapiens	human	Chordata	Mammalia	Primates	NM_007215.3
Gallus gallus	chicken	Chordata	Aves	Galliformes	XM_415670.3
Taeniopygia guttata	zebra finch	Chordata	Aves	Passeriformes	XM_002194622.1
Meleagris gallopavo	chicken	Chordata	Aves	Galliformes	XM_003211538.1

Table S2. Mitochondrial genomic features and mode of mtDNA replication in Vertebrata, Tunicata, Arthropoda and Nematoda

Animal Group	Gene content	Number of distinct genome architectures[1]	Gene strand asymmetry[2]	Main mode of mtDNA replication[3]
Vertebrata	Typical 37 genes[4]	43/ 849 genomes sequenced (5)[5]	28 genes in one strand, 9 in the other	RITOLS/ theta-like and strand-displacement
Tunicata	Typical 37 genes plus two extra tRNA genes[4]	21/ 24 genomes sequenced (88)[6]	Maximal - all genes encoded in one strand	Not yet determined
Arthropoda	Typical 37 genes[4] (duplications or losses of tRNA genes are frequent)	70/ 154 genomes sequenced (45)[5] (mostly due to tRNA gene rearrangements)	For most insects, 23 genes in one strand, 14 in the other; high variability in other groups	Theta-like
Nematoda	Lacking the ATP8 gene[4] in most species/ extra tRNA genes in some species	17/ 26 genomes sequenced (65)[5] 26[7]/ 103 genomes sequenced (25)[6]	Maximal - all genes encoded in one strand for most species	Not yet determined

[1] defined as the number of different, non-random arrangements of functional elements (genes and non-coding regions) in the genome

[2] differences in the number of genes encoded in both strands of the mtDNA

[3] See the main text for details.

[4] See Figure S3 for details.

[5] according to the data presented in Gissi *et al.* (2008)

[6] Retrieved from Organelle Genome Resources, NCBI - January, 2015

[7] based solely on the organization of protein-coding genes; the actual number of distinct genome architectures, which include tRNA and rRNA gene rearrangements, is likely to be significantly higher

```
Saccharomyces cerevisiae        M------------------------------------------------- 1
Amphimedon queenslandica        M------------------------------------------------- 1
Trichoplax adhaerens            M------------------------------------------------- 1
Nematostella vectensis          M------------------------------------------------- 1
Crassostrea gigas               M------------------------------------------------- 1
Caenorhabditis elegans          M------------------------------------------------- 1
Oscheius tipulae                M------------------------------------------------- 1
Loa loa                         M------------------------------------------------- 1
Bursaphelenchus xylophilus      M------------------------------------------------- 1
Dirofilaria immitis             --------------------------------------------------
Daphnia pulex                   M------------------------------------------------- 1
Pediculus humanus corporis      M------------------------------------------------- 1
Drosophila melanogaster         M------------------------------------------------- 1
Drosophila erecta               M------------------------------------------------- 1
Drosophila simulans             M------------------------------------------------- 1
Drosophila virilis              M------------------------------------------------- 1
Drosophila willistoni           M------------------------------------------------- 1
Drosophila mojavensis           M------------------------------------------------- 1
Drosophila grimshawi            M------------------------------------------------- 1
Culex quinquefasciatus          M------------------------------------------------- 1
Anopheles gambiae               M------------------------------------------------- 1
Tribolium castaneum             M------------------------------------------------- 1
Apis mellifera                  M------------------------------------------------- 1
Bombus impatiens                M------------------------------------------------- 1
Bombus terrestris               M------------------------------------------------- 1
Nasonia vitripennis             MFLLSDAG------------------------------------------ 8
Danaus plexippus                M------------------------------------------------- 1
Strongylocentrotus purpuratus   MFG-----V---------------------------------------GK 6
Saccoglossus kowalevskii        M------------------------------------------------- 1
Branchiostoma floridae          M------------------------------------------------- 1
Ciona intestinalis              --------------------------------------------------
Oreochromis niloticus           M------------------------------------------------- 1
Danio rerio                     M------------------------------------------------- 1
Xenopus tropicalis              M------------------------------------------------- 1
Anolis carolinensis             M------------------------------------------------- 1
Meleagris gallopavo             M------------------------------------------------- 1
Gallus gallus                   --------------------------------------------------
Monodelphis domestica           MWGGGEGDLYLTLLEENSLQEPPANVLKTSDGRLGKDPKAVSGQKWEAAA 50
Mus musculus                    M------------------------------------------------- 1
Ailuropoda melanoleuca          M------------------------------------------------- 1
```

```
Loxodonta africana            M------------------------------------------------- 1
Callithrix jacchus            M------------------------------------------------- 1
Pongo abelii                  M------------------------------------------------- 1
Homo sapiens                  M------------------------------------------------- 1

Saccharomyces cerevisiae      ----------------------------TKLMVRSECMLRMVRRRPLRVQ 23
Amphimedon queenslandica      -----------------------------------------------SVP 4
Trichoplax adhaerens          ------------------VTAMAGRRMLEKLATNHAKTMTMWRAIFKSAR 33
Nematostella vectensis        -------------------TGWWASPSACCSAWRTDLNGLVIVPSPSSWP 32
Crassostrea gigas             -----------ASYLNNVRRGYHSCSIERMLALKRGSNTFLRSS------ 34
Caenorhabditis elegans        ------------------------------------------------RP 3
Oscheius tipulae              --------------------------------------------------
Loa loa                       -------------------SVRCGSSVVRKSWSFAITRWYTVGVNSAEQP 32
Bursaphelenchus xylophilus    --------------------VKCDSEHEMLRRALSLSSRRYAPMVPHQTP 31
Dirofilaria immitis           --------------------------------------------------
Daphnia pulex                 --------------------------------------------------
Pediculus humanus corporis    --------KNFTQIILKPENSRIKLRWLYKKKFYSTQTDFYIKDNSNKLP 43
Drosophila melanogaster       --------------------------QFHLIRKYAS-----------K-- 12
Drosophila erecta             --------------------------QLHLIRKYAS-----------K-- 12
Drosophila simulans           --------------------------QLHLIRKYAS-----------K-- 12
Drosophila virilis            ----------------------------RLLRCYAT-----------K-- 10
Drosophila willistoni         --------TDDLLCNSNLESIKSKETAMILLRCYTT-----------RAP 32
Drosophila mojavensis         ----------------------------RLLRWYAT-----------K-- 10
Drosophila grimshawi          ----------------------------HPLRCYAT-----------K-- 10
Culex quinquefasciatus        ------------------------QRIAYIRRYCGT-----------KSR 16
Anopheles gambiae             -------------------------RLLAHRRWYGV-----------VTP 15
Tribolium castaneum           --------------------------------------------------
Apis mellifera                --------------------------------------------------
Bombus impatiens              ---------KSRVNIAMKLQKIVRSKLIMNRCIINICNRHKSDMINHNIS 42
Bombus terrestris             -----------------------------KRRIINMCNRHKSNVINCNVS 22
Nasonia vitripennis           --------------------------------------------------
Danaus plexippus              -------------------------------------RNIKKLCHIYRRG 14
Strongylocentrotus purpuratus GCRKSVVHLCSYCRLIHGRPLYPSARFRKTLPWQHE---STTSSSPFSTS 53
Saccoglossus kowalevskii      ----------------------------ATRKILQCVPSIFHQNNVTFLP 23
Branchiostoma floridae        -------------------------SATA-------------KLLPLWAP 13
Ciona intestinalis            --------------------------------------------------
Oreochromis niloticus         --------------------------------APLCKPLLRQGIRQGLCP 19
Danio rerio                   -------------------------LRLISCHRWQTF------------- 13
Xenopus tropicalis            ------------------------NRLL--------------RKATPLFP 13
Anolis carolinensis           --------------------------------------------------
```

```
Meleagris gallopavo             --------------------------------------------------
Gallus gallus                   --------------------------------------------------
Monodelphis domestica           GCSSNSNSSDEETGSERLKELPKDTQLMSRLVWRVI-------AARGVLP 93
Mus musculus                    ------------------------SRLL----WKKV-AGA--KVASGPVP 20
Ailuropoda melanoleuca          ------------------------SRLL----WKRE-AGAATAVGPGPVP 22
Loxodonta africana              ------------------------SRLL----WKKV-AGA---VVPGPVP 19
Callithrix jacchus              ------------------------SRLL----WRKVAAGA--TVGPGLAP 21
Pongo abelii                    ------------------------VKPISLTGEQKT-GRV--SLQRFPAH 24
Homo sapiens                    ------------------------SRLL----WRKV-AGA--TVGPGPVP 20

Saccharomyces cerevisiae        -FCARWFSTK---------------------------------------- 32
Amphimedon queenslandica        KFGCRFFI------------------------------------------ 12
Trichoplax adhaerens            -SPTFVYIRKRF-------------------------------------- 44
Nematostella vectensis          -SFSEVYRIGPFAWRVISAVFSASSPSAIIIIINIPFYSVI--------- 72
Crassostrea gigas               --------------------------------------------------
Caenorhabditis elegans          PKIMQIFHVSRRQ------------------------------------- 16
Oscheius tipulae                --------------------------------------------------
Loa loa                         --------------------------------------------------
Bursaphelenchus xylophilus      --------------------------------------------------
Dirofilaria immitis             --------------------------------------------------
Daphnia pulex                   --------------------------------------------------
Pediculus humanus corporis      -KSNKVLTSTILKTEEEERK------------------------------ 62
Drosophila melanogaster         -VSREHYASSSVKIFRRVKP-PQKVNK----------------------- 37
Drosophila erecta               -ISRDHYASSSVKIYRRVKP-PQKVNK----------------------- 37
Drosophila simulans             -ASREHYASSSVKIFRRVKP-QQKVNK----------------------- 37
Drosophila virilis              -AGREHYASSNVKIFRKL-PSKTKLKS----------------------- 35
Drosophila willistoni           -APREHYASSSIKIYRKARAPKQKQLK----------------------- 58
Drosophila mojavensis           -AGREHYASSSIKVYRKL-PSKTKVRT----------------------- 35
Drosophila grimshawi            -AGREHYASSSVKIYRKLQPSTIKPK------------------------ 35
Culex quinquefasciatus          -AVVEIFPRSTVRRLNLPDSKCEEPS------------------------ 41
Anopheles gambiae               -SQSDILPHSTVRRLKVA-------------------------------- 32
Tribolium castaneum             --------------------------------------------------
Apis mellifera                  --------------------------------------------------
Bombus impatiens                ANESKRFKKKIISIKKCGQLLTLPENISMCNQKHENDVNKDYIAKNSVVL 92
Bombus terrestris               VSEGKQFEKRNISIKKCGPLLTLPENISMCNQKHESDVNKDYKAKNSVVL 72
Nasonia vitripennis             --------------------------------------------------
Danaus plexippus                -YSNDILPSSESVIIRQNKPEKDIDNLETPVEIHP--------------- 48
Strongylocentrotus purpuratus   -DQNHLSKDDSS-----VGKPEESHSSSSV-------------------- 77
Saccoglossus kowalevskii        -SVSNGLH---------VHRFSAATSK----------------------- 40
Branchiostoma floridae          -SVCRFAHHVHP-----VAQHSWI-------------------------- 31
```

```
Ciona intestinalis            --------------------------------------------------
Oreochromis niloticus         -PPGTGQAPTLA-----VQMASGA-------------------------- 37
Danio rerio                   ------VRSGCW-----VRRCSTS-------------------------- 26
Xenopus tropicalis            ----SWKTRGCG-----YRKCSYAPQL----------------------- 31
Anolis carolinensis           --------------------------------------------------
Meleagris gallopavo           --------------------------------------------------
Gallus gallus                 --------------------------------------------------
Monodelphis domestica         WCFQRWASGSSVS----QQFCEAE-------------------------- 113
Mus musculus                  -ATARWVSSSVL-----DPVPSDG----------------RP-------- 40
Ailuropoda melanoleuca        -APGRWVSSSGP-----DPVPSDG--------------QPQP-------P 45
Loxodonta africana            -ALGRWVSSSAP-----VPDPSDG--------------QPQP-------L 42
Callithrix jacchus            -APGRWVSSSVL-----ASDPSDGRR---------QQLQQQP-------- 48
Pongo abelii                  -SRGAGVLSLCPRCRTHRERATGGRG---------QQQQQQPSEHNSRAQ 64
Homo sapiens                  -APGRWVSSSVP-----ASDPSDGQRRRQQQQQQQQQQQQQP-------Q 57

Saccharomyces cerevisiae      --------------------------------------------------
Amphimedon queenslandica      --------------------------------------------------
Trichoplax adhaerens          --------------------------------------------------
Nematostella vectensis        --------------------------------------------------
Crassostrea gigas             --------------VCK--------------------------------- 37
Caenorhabditis elegans        --------------------------------------------------
Oscheius tipulae              --------------------------------------------------
Loa loa                       --------------------------------------------------
Bursaphelenchus xylophilus    --------------------------------------------------
Dirofilaria immitis           --------------------------------------------------
Daphnia pulex                 --------------------------------------------------
Pediculus humanus corporis    --------------VIK--------------------------------- 65
Drosophila melanogaster       --------------------------------------------------
Drosophila erecta             --------------------------------------------------
Drosophila simulans           --------------------------------------------------
Drosophila virilis            --------------------------------------------------
Drosophila willistoni         --------------------------------------------------
Drosophila mojavensis         --------------------------------------------------
Drosophila grimshawi          --------------------------------------------------
Culex quinquefasciatus        --------------------------------------------------
Anopheles gambiae             --------------------------------------------------
Tribolium castaneum           TNSNTFLNTVPFTAPLI--------------------------------- 18
Apis mellifera                --------SLRNISEIA--------------------------------- 10
Bombus impatiens              PNKSQVRVEYEDLSVIPKNFEQSLYPLIGSAKSNYNNSLIEHKDIQTLEN 142
Bombus terrestris             PNKSQVREEYEDLSVIPKNFEQSLYPLIGNAKSNYKNSLIEHKDIQTLEN 122
```

Nasonia vitripennis	--	
Danaus plexippus	--	
Strongylocentrotus purpuratus	--	
Saccoglossus kowalevskii	--	
Branchiostoma floridae	--	
Ciona intestinalis	--	
Oreochromis niloticus	--	
Danio rerio	--	
Xenopus tropicalis	--	
Anolis carolinensis	--	
Meleagris gallopavo	--	
Gallus gallus	--	
Monodelphis domestica	--	
Mus musculus	--	
Ailuropoda melanoleuca	--	
Loxodonta africana	--	
Callithrix jacchus	--	
Pongo abelii	--	
Homo sapiens	--	

Saccharomyces cerevisiae	-----------KNTAEAPRINPVGIQYL----------------------	49
Amphimedon queenslandica	-----------RNLSDQTRFNPLGVQML----------------------	29
Trichoplax adhaerens	-----------YCQHREHRVNAINIQML----------------------	61
Nematostella vectensis	-----------YVKLCQFRYNPVNIQML----------------------	89
Crassostrea gigas	VNVHHSCSVK-TASTAKSRVNEINIQMI----------------------	64
Caenorhabditis elegans	-----------IATSKTSNFERKTIETV----------------------	33
Oscheius tipulae	-----------FMACDAMKLSRCSQNLA----------------------	18
Loa loa	-----------STSKRNASFSLRKIDLV----------------------	49
Bursaphelenchus xylophilus	------------TSFKPSDLISQSIKIV----------------------	47
Dirofilaria immitis	--	
Daphnia pulex	-----EGTTMPSGVQYCYLVRMISLQKIDSKGEVVPSIFRLIGSHRDRAR	46
Pediculus humanus corporis	KKCVDENVNE-VENKPSSRINELNIQML----------------------	92
Drosophila melanogaster	---PKKPENV-ENGPTEYAENLVKVQMI----------------------	61
Drosophila erecta	---PKKSENV-EDGQSEYAENLVKVQMI----------------------	61
Drosophila simulans	---PKKPENV-EDGQTEYAENLVKVQMI----------------------	61
Drosophila virilis	KHALPTTSTT-SPANVEYAENLVKVQMI----------------------	62
Drosophila willistoni	--NSEPTPVA-GSKANESFQNPVKIQMI----------------------	83
Drosophila mojavensis	-EHVPSAQTK-AAQNVEYAENLVKVQMI----------------------	61
Drosophila grimshawi	---PKPKHVP-QPASNEYAENLVKVQMI----------------------	59
Culex quinquefasciatus	-----------PAQPPGVRLNEMNIQLL----------------------	58

```
Anopheles gambiae               ---PKPPPAK-PPDDRGPRLNEMSIQML---------------------- 56
Tribolium castaneum             KKTIPPSPCT-FEVCVGPRVNQFNIQML---------------------- 45
Apis mellifera                  ENKEEEICNE-RISENDIRINEINIQML---------------------- 37
Bombus impatiens                ENNQKQTYNK-RTNDNDVRTNEINMQML---------------------- 169
Bombus terrestris               ENNQKQTYNE-RTNDSDIRINEINMQML---------------------- 149
Nasonia vitripennis             -----------GLASKRLRKPLISIKRL---------------------- 25
Danaus plexippus                -----------ENDPKEFRVNDVNIQMI---------------------- 65
Strongylocentrotus purpuratus   -----SVDST-LDTSPQLRMNAINIQML---------------------- 99
Saccoglossus kowalevskii        -----AIDQE-KTEEESTRYNPIHIQML---------------------- 62
Branchiostoma floridae          -----PAMQY-CSSTIPTRMNPIDIQML---------------------- 53
Ciona intestinalis              --------------------NKFGIQML---------------------- 8
Oreochromis niloticus           -----LGRPL-QLDSTQTRLNPLNIQML---------------------- 59
Danio rerio                     -----VKSKP-QQGSDQTRLNPLNIQML---------------------- 48
Xenopus tropicalis              -----QTETL-ETEMSQRRMNPLNIQML---------------------- 53
Anolis carolinensis             ------AQDA-NAASKQLRMNPLGIQML---------------------- 22
Meleagris gallopavo             --------------------------------------------------
Gallus gallus                   --------------------------------------------------
Monodelphis domestica           -----ASSVG-LRDECQKRENALHIQML---------------------- 135
Mus musculus                    -----PSQMP-SSENGQLRLNPLLIQML---------------------- 62
Ailuropoda melanoleuca          -----PQQAP-SSEGGQLRHNPLHIQML---------------------- 67
Loxodonta africana              -----QQQMP-SSEGGQPRYNPLRIQML---------------------- 64
Callithrix jacchus              ------PQVP-SSEGGQLRHNPLHIQML---------------------- 69
Pongo abelii                    -----QAQCPISREGGQLRHNPMHIQML---------------------- 87
Homo sapiens                    -----QPQVL-SSEGGQLRHNPLDIQML---------------------- 79

Saccharomyces cerevisiae        ----G---------ESLQRQVFGS------CGGKDEVEQSD--------- 71
Amphimedon queenslandica        ----S---------KSLYQQVFPG----------AESQTEPSQ------- 49
Trichoplax adhaerens            ----S---------DTLHRQIFRR----------GNDQNASNAHDQYIDG 88
Nematostella vectensis          ----S---------ASLHEQLFPD----------TPANTQST-------- 108
Crassostrea gigas               ----P---------EKLHEQIFPK----------ADTKEKKVV------- 84
Caenorhabditis elegans          ----C---------PRVHNYLFPH-------------------------- 44
Oscheius tipulae                ----A----------------FWK-------------------------- 22
Loa loa                         ----P---------ERLHRHLFGN-------------------------- 60
Bursaphelenchus xylophilus      ----P---------NPVNRYLFGP-------------------------- 58
Dirofilaria immitis             --------------------------------------------------
Daphnia pulex                   EFGHA---------SRLHGSGYKS----------TGLNH----------- 66
Pediculus humanus corporis      ----S---------DNLHGQIFKD----------GNLSSSDNN------- 112
Drosophila melanogaster         ----S---------RNLHAQLFPQ----------APRSIS------EQQV 82
Drosophila erecta               ----S---------RNLHAQLFPQ----------TPRSIS------EQQV 82
Drosophila simulans             ----S---------RNLHAQLFPQ----------APRSIS------EQQV 82
```

```
Drosophila virilis            ----S---------QNLHRQLFPQ----------AKREYT------NAER 83
Drosophila willistoni         ----S---------QNLHKQIFPQ----------SPRRNL------AESV 104
Drosophila mojavensis         ----S---------QNLHAQLFPQ----------SKREYS------EAEH 82
Drosophila grimshawi          ----S---------QNLHGQLFPQ----------AHRKYT------ESER 80
Culex quinquefasciatus        ----S---------AGLHRQVFGD----------AAKQQKVDT------- 78
Anopheles gambiae             ----S---------DSLYRQIFRP----------TAQHGRH----APASP 79
Tribolium castaneum           ----S---------PHLFEQVFKG----------LSVNKCDE-------- 64
Apis mellifera                ----P---------KSLHKQIFKN----------SETPQK---------- 54
Bombus impatiens              ----P---------KSLYKQIFKN----------CSKQEKIPE------- 189
Bombus terrestris             ----P---------KSLYEQIFKN----------SSKQEKISK------- 169
Nasonia vitripennis           ----ACEATNFLSGGAMNQHKFNS----------------------TGLI 49
Danaus plexippus              ----S---------KNIYDQLFRT----------PQPTLDA--------- 83
Strongylocentrotus purpuratus ----S---------KNLHQRIFNRKASSRSKVKQATETKND--------- 127
Saccoglossus kowalevskii      ----S---------KSLHQQIFREKFGEV-----SNTESQE--------- 85
Branchiostoma floridae        ----Y---------KPLHDQIFK-----------QEPTYPT--------- 70
Ciona intestinalis            ----P---------EKLHKQIFKE----------TRIKDENP-------- 27
Oreochromis niloticus         ----S---------KGLHEQIFQG----------LEPEYRE--------- 77
Danio rerio                   ----S---------RNLQEQIFRG----------QTQEYTD--------- 66
Xenopus tropicalis            ----S---------KGLHAQVFRG----------KQVQHAE--------- 71
Anolis carolinensis           ----S---------KGLHEQIFRG----------AQVQYSE--------- 40
Meleagris gallopavo           ------------------EQI----------------------------- 4
Gallus gallus                 --------------------------------------------------
Monodelphis domestica         ----S---------RGLHEQIFGQ----------PEMQPSE--------- 153
Mus musculus                  ----S---------RGLHEQIFGC----------GGEMPDE--------- 80
Ailuropoda melanoleuca        ----S---------RGLHEQIFGR----------GGETPGE--------- 85
Loxodonta africana            ----S---------RGLHDQIFGH----------GGEMPDE--------- 82
Callithrix jacchus            ----S---------RGLHEQIFGQ----------GGEMPGE--------- 87
Pongo abelii                  ----S---------RGLHEQIFG-----------GGEMPGE--------- 104
Homo sapiens                  ----S---------RGLHEQIFGQ----------GGEMPGE--------- 97

Saccharomyces cerevisiae      KLMELSKKSLKDHGLWGKKTLIT-----DPISFPLPPLQGRSLD--EHFQ 114
Amphimedon queenslandica      EAINKSVSHLSEHGLWTNGSGTT--VTQENIDINLPPLFGENIL--SHFT 95
Trichoplax adhaerens          ELLEKIKNHLQQHDLADKQTSKQ-----EDVAFQLPELHGSNID--EHFR 131
Nematostella vectensis        DVIQKCIEHLSAHGLWGKAKSAA-----RDVDMTLPPLLGENID--DHFR 151
Crassostrea gigas             SGLDDIKNNLAAHGLWDKNTTVL-----DDVTFDLPKLHGNSVT--DHFY 127
Caenorhabditis elegans        --------------VSAPSTSSA-----AEYTGVLPPLVADNVV--DHFG 73
Oscheius tipulae              ----------------------------------------------RLFR 26
Loa loa                       ---YPVPENTLKHD--------------PFEALDLPQLNGSDLL--DHFE 91
Bursaphelenchus xylophilus    -----------------LNTAGPPGLDPIFSKLQLPPLNG-TVL--EHFK 88
Dirofilaria immitis           --------------------------------------------------
```

Daphnia pulex	------RQHLSIHHLYGKSPSKL-----PDIDLKLPTLLGKNIA--EHFE	103
Pediculus humanus corporis	ILIKNSKKLLKQHGLWTNDIKLL-----EDVELDLPPLKGKNIE--EHFW	155
Drosophila melanogaster	ASAKVYKDELRRHGVDIESSAPV-----SDVQLKLPALRGANIE--EHFH	125
Drosophila erecta	ASAKAYKEELRRHGVDIESSAPV-----SDVQLKLPPLRGANIE--EHFH	125
Drosophila simulans	ASAKVYKDELRRHGVDIESSAPV-----SDVQLKLPPLRGANIE--EHFH	125
Drosophila virilis	VAAKLYNAELQRHGIDVASTEPM-----PDVKLKLPPLRGKNIE--EHFY	126
Drosophila willistoni	AAARDYKADLHRHGIDIDSSMPI-----EDVNLELPPLHGENIE--EHFY	147
Drosophila mojavensis	LAAKAYNAELLRHGIDVTSTSPI-----PDVELKLPPLRGRNIE--EHFH	125
Drosophila grimshawi	ATAKLYNAELLRHGIDVKSTTAV-----PDVQLKLPPLRGTNIE--EHFH	123
Culex quinquefasciatus	SKLESLRKELTRHGIPLDNPDIR-----PDVDFRLPRLRGVGIE--EHFF	121
Anopheles gambiae	TLVKSLRDDLLRHGIDASSPALL-----PDVDVKLPPLRGANIE--EHFH	122
Tribolium castaneum	AIVKECLKTLQEHNMVQKPENFL-----NDVELKIPPLEGKNIE--EHFH	107
Apis mellifera	--------------ISKEDATRL-----PKVDIKIPLLEGDDIE--QHFY	83
Bombus impatiens	EEIEAIKNNLRSYGINTEDASRL-----PDVNIKIPPLEGDNIQ--EHFY	232
Bombus terrestris	EEIEAIKKDLRSYGINTEDASRL-----PDVNIKIPPLEGDNIE--EHFY	212
Nasonia vitripennis	TETRTNSNLMVNYRSSSTSSMMLFSVHYPMDDSQIPRVEIGSIDKFKKYK	99
Danaus plexippus	GLIKSCLNHLEKHGIDIKKSTYL-----PDVQLKIPKLQGKDIE--EHFF	126
Strongylocentrotus purpuratus	ELLEKTKEHLENHRLWGKEGSIL-----PDVDFKLPRLFGSSID--EHFQ	170
Saccoglossus kowalevskii	KKINKSIKHLKRHGLWGKQSTLL-----PEVDFQLPELYGDNIE--DHFR	128
Branchiostoma floridae	EDVERSVHHLKTHDLWGKETSAL-----PEVDFKLPDLLGSNID--EHFK	113
Ciona intestinalis	KQIQQSIEHLDKFGLWGKEQESL-----PDYDVTLPKLYGNNVN--EHFE	70
Oreochromis niloticus	EAVQRSIRHLQKHQLWGKDTSLL-----PDVDLKLPKMYGKNID--EHFR	120
Danio rerio	EDVERSIRHLEHHGLWGKETSLL-----PDVQLRLPEMYGSNID--EHFR	109
Xenopus tropicalis	EDIQRSINHLKSHELWGQETSTV-----PDIELQLPKLYGNDIE--EHFQ	114
Anolis carolinensis	EDIQKSVEHLRKHDLWGKETSTL-----PDVELRLPRMYGSNID--EHFR	83
Meleagris gallopavo	--VPLE	8
Gallus gallus	--	
Monodelphis domestica	EAIQQSVEHLQKHGLWGQETSAL-----PDVELQLPRLYGEDLD--EHFR	196
Mus musculus	AAVQRSVEHLQKHGLWGQPATPL-----PDVELRLPRLFGGNLD--QHFR	123
Ailuropoda melanoleuca	AAVRRSVEHLQKHGLWGQPATPL-----PDVELRLPPLYGGSLD--QHFR	128
Loxodonta africana	AAVRRSVEHLQRHGLWGQPAASL-----PDVELCLPPLYGGNLD--QHFR	125
Callithrix jacchus	AAVRRSVEHLQKHGLWEQPATPL-----PDVELRLPPLYGDNLD--EHFR	130
Pongo abelii	AAVRRSVEHLQKHGLWGQPAAPL-----PDVELRLPPLYGDNLD--QHFR	147
Homo sapiens	AAVRRSVEHLQKHGLWGQPAVPL-----PDVELRLPPLYGDNLD--QHFR	140
Saccharomyces cerevisiae	KIGRFNSEPYKSFCEDKFT--------------------------EMVA-	137
Amphimedon queenslandica	KLAEDQVSPYRPLIASLVC------------------------EGSLSS-	120
Trichoplax adhaerens	YIANQQIAHYKILIDRLID-------------------------ATLPS-	155
Nematostella vectensis	TIARQQSKAYYDMSQDIAS-------------------------SSLPS-	175
Crassostrea gigas	SIANQQLDGYIQLTQMLMG-------------------------GPIPP-	151

```
Caenorhabditis elegans         VLAEQQTRKYKELLEKACE-------------------------FDLETA 98
Oscheius tipulae               IIGSEQAAPYLQLLQKTAEQFSYKYNVCDNDDWTRNLVAEGSSCTKFRN- 75
Loa loa                        NTAIKQFEPYRRLLVEATT------------------------IRKLPV- 116
Bursaphelenchus xylophilus     HIGQELFGPFETLLEKAAN-------------------------LKIPK- 112
Dirofilaria immitis            --------------------------------------------------
Daphnia pulex                  KIAEKQCGSYRNKLLDLTS-------------------------HCIPD- 127
Pediculus humanus corporis     ILGEEQSKPYRNLLLNFIS-------------------------KKLPK- 179
Drosophila melanogaster        NIAKEQVQPYEELLLPLVQ------------------------CEQLPK- 150
Drosophila erecta              NIAQEQVQPYEELLLPLVQ------------------------CEKLPK- 150
Drosophila simulans            NIAKEQVQPYEELLLPLVQ------------------------CEQLPK- 150
Drosophila virilis             AIAKEQVAPYEALLQPLVA------------------------CQQLPA- 151
Drosophila willistoni          NIAKEQVKPYEELLWPLVK------------------------CRELPN- 172
Drosophila mojavensis          SIAEEQVAPYKTLLQALIA------------------------CKELPA- 150
Drosophila grimshawi           AIAKEQVAPYEALLHPLVK------------------------CSKLPA- 148
Culex quinquefasciatus         NIAREQSKPYRDLLEALVR-------------------------GEVPS- 145
Anopheles gambiae              SIAEEQSEPYRRAIEQLPG--------------------------APPN- 145
Tribolium castaneum            LIGDQQVKPYRDLVEKLLT-------------------------NKIPK- 131
Apis mellifera                 NIGKAQIKPYLKLIKNIMK--------------------------NIPQ- 106
Bombus impatiens               NIANKQIEPYIKLIKNIMK--------------------------DIPP- 255
Bombus terrestris              NISNTQIKPYIKLIKNIMK--------------------------DIPP- 235
Nasonia vitripennis            VIIEEET---RDALIVFFE-------------------------DDLEK- 120
Danaus plexippus               NIGETQC--FKNLMKDGMR------------------------------- 143
Strongylocentrotus purpuratus  ILANKQSASYLDMAHELAE-------------------------TLPPP- 194
Saccoglossus kowalevskii       VLAQQQSNTYLMLAESLAE-------------------------AKLPT- 152
Branchiostoma floridae         EIAKQQSKPYLDAAQVIAN-------------------------SRLPT- 137
Ciona intestinalis             NVASEQSRPYREALNKLVK-------------------------NELPN- 94
Oreochromis niloticus          HLAQTQSLPYLEAATKLKL-------------------------AELPP- 144
Danio rerio                    LLAQKQSLPYLEAASQLQK-------------------------AQIPE- 133
Xenopus tropicalis             ILAQKQSLPYLEAANDLMN-------------------------CQLPT- 138
Anolis carolinensis            ILAQKQSLPYLEAAKELLQ-------------------------CEMPA- 107
Meleagris gallopavo            ACAYSQS-----------------------------------------T- 16
Gallus gallus                  --------------------------------------------------
Monodelphis domestica          ILAQKQSLPYLEAAHELLQ-------------------------AELPP- 220
Mus musculus                   LLAQKQSLPYLEAAASLLE-------------------------AQLPP- 147
Ailuropoda melanoleuca         LLAQKQSLPYLEAANSLLQ-------------------------AQLPP- 152
Loxodonta africana             LLAQKQSLPYLEAANLLLQ-------------------------AQLPP- 149
Callithrix jacchus             LLAQKQSLPYLEAANSLLQ-------------------------AQLPP- 154
Pongo abelii                   LLAQKQSLPYLEAANLLLQ-------------------------AQLPP- 171
Homo sapiens                   LLAQKQSLPYLEAANLLLQ-------------------------AQLPP- 164
```

Saccharomyces cerevisiae	--------RP--AEWLRKPGWVKYVP-GMA---PVEVAYPDEELVVFDVE	173
Amphimedon queenslandica	--------PP--TQWNYKPGWTCYSN-DGS---ITLVPFPDEKALIFDVE	156
Trichoplax adhaerens	--------KP--LTWECRPGWTRYD--GNQ---ATSVEFPDEEALVFDVE	190
Nematostella vectensis	--------VP--DMWEFRAGWCRYAE-DADGLHVTQVECPPDDALVFDVE	214
Crassostrea gigas	--------KP--KKWSQRAGWTKYDPKTGK---ATAVKYPEEDIFVFDVE	188
Caenorhabditis elegans	KNVLEHIDKT--DGWHYKTGWTRYPFSTSEP--PETIDFPRDSILFFDIE	144
Oscheius tipulae	--------LSD-IDWKFAKGWTRYSHEDGS---TRSVPFPEEDVLIFDVE	113
Loa loa	--------IP--EKWIFHPGWTRYGM-NES---PKQVDHPLEDLIFFDVE	152
Bursaphelenchus xylophilus	--------KP--KTWEYKIGWTKYLE-DGR---TVAVDAPDADLLFFDVE	148
Dirofilaria immitis	--	
Daphnia pulex	--------IP--QVWNFAPGWTRYNQ-NGE---MDLVDFPEEDAYVFDVE	163
Pediculus humanus corporis	--------SP--DKWLCQKGWTKYEE-GKE---PVQVQFPDEDALIVDVE	215
Drosophila melanogaster	--------RP--KRWAFHTGWTAYDPEDGT---ATPVDHPLEKGLVFDVE	187
Drosophila erecta	--------RP--KRWAFFTGWTAYDPDDGT---ATPVDHPLEKGLVFDVE	187
Drosophila simulans	--------RP--KRWAFHTGWTAYDPEDGT---ATPVDHPLEKGLVFDVE	187
Drosophila virilis	--------KP--KRWAFYAGWTAYDPVDGS---ATPVPQPLEKGIIFDVE	188
Drosophila willistoni	--------KP--KRWAFHAGWTAYNPIDGT---PEAVDQPLEKGLIFDVE	209
Drosophila mojavensis	--------KP--KRWAFHAGWTAYDPVSGT---ATPVAQPLETGIIFDVE	187
Drosophila grimshawi	--------KP--KRWAFYAGWTAYDPEDGT---ATPVAHPLEQGIIFDVE	185
Culex quinquefasciatus	--------IP--KKWSEEPGWTCYDPLRG----AVAVPYPEDNALVFDVE	181
Anopheles gambiae	--------PP--KVWSRTAGWTRYDPVTGS---ASSIPFPDDPALVFDVE	182
Tribolium castaneum	--------PP--DKWVMQEGWTRYVP-GEE---PQQVPYPLEEGLIFDIE	167
Apis mellifera	--------MP--NQWILNEGWTRYTV-DN----IQKVDYPLEDAIIFDVE	141
Bombus impatiens	--------MP--NQWLLNEGWTRYSV-DG----VQKVDHPLEDAIIFDVE	290
Bombus terrestris	--------MP--NQWLLNEGWTRYSV-DG----VQKVDHPLEDAIIFDVE	270
Nasonia vitripennis	--------LERKISKVSDSPQSKKDNLKSI---SKTVDI--LGAKLKDIS	157
Danaus plexippus	---------------------------------RILVAFNG-----FTVK	155
Strongylocentrotus purpuratus	--------MP--EDWVFKEGWMKYVE-NES---PVPVDFPEDQSLVFDME	230
Saccoglossus kowalevskii	--------MP--TEWIKEYGWTKYES-NGS---TMSVAYPEDRALVFDVE	188
Branchiostoma floridae	--------MP--REWRWSRGWTKYDA-TGH---VTTVDYPDEEAIVFDVE	173
Ciona intestinalis	--------MPGLDDWVDQPGWTRYTYFNGK-LMTEPVEYPAGDAVVFDCE	135
Oreochromis niloticus	--------MP--QEWSWEVGWTRYGP-TGD---RQKVDFPDESALVFDVE	180
Danio rerio	--------MP--QEWAWEVGWTRYGA-DGE---SQKVNFPDENALVFDVE	169
Xenopus tropicalis	--------MP--QTWAWQSGWTRYTA-TGE---TELVDFPDEKALVFDVE	174
Anolis carolinensis	--------MP--LEWAWEAGWTQYGP-GGE---RKAVECPGERAMVFDVE	143
Meleagris gallopavo	--------NP-----------------------RDYVCESRENVFIAVII	35
Gallus gallus	--	
Monodelphis domestica	--------LP--NKWAWKEGWTRYEP-DGK---GQQVEFPEERALVFDVE	256
Mus musculus	--------EP--KSWAWAEGWTRYGP-EGE---AEPVAIPEERALVFDVE	183
Ailuropoda melanoleuca	--------RP--PSWAWAKGWTRYGP-AGE---AEPVAIPEERALVFDVE	188

Loxodonta africana	--------QP--PSWAWAEGWTRYGP-GGE---AEPVAIPEERALVFDVE	185
Callithrix jacchus	--------QP--PSWAWAEGWTRYGP-EGE---AVPVAIPEERALVFDVE	190
Pongo abelii	--------KP--PAWAWAEGWTRYGP-EGE---AVPVAIPEERALVFDVE	207
Homo sapiens	--------KP--PAWAWAEGWTRYGP-EGE---AVPVAIPEERALVFDVE	200
Saccharomyces cerevisiae	TLYNVSDYPTLATALSSTA-WYLWCSPFICGGD----------------	205
Amphimedon queenslandica	VCVPEGHAPKLAIAMSPNN-VYSWVSPRLFSERDFA-------------	191
Trichoplax adhaerens	TSVMNGRIPVMATAVSPTH-WYSWVSHHLILDD-LR-------------	224
Nematostella vectensis	VLQSEGLSPTLAVAASPSA--YSWVSPRLVKDKSYQ-------------	248
Crassostrea gigas	ILVMEGHYPTMATALSPTN-WYSWCSEAVTKDK-FR-------------	222
Caenorhabditis elegans	LVVRDGTLPTLAIALGRDA-WYGWCSDRLIYESE---------------	177
Oscheius tipulae	VCLKDGLLPTLAVALSDKA-WYGWCSERLTEDTP---------------	146
Loa loa	VCVRDGLLPTLATAVTPKA-WYSWCSDRLVNGGD---------------	185
Bursaphelenchus xylophilus	VCVKEGHLPTLAVAVSETH------CGRLVEDTP---------------	176
Dirofilaria immitis	--	
Daphnia pulex	VCVKDGNLPTLATAVSKNY-WYSWCSNQLFTDNKVQ----------SSIL	202
Pediculus humanus corporis	VCVSVGNLPTLATAVSENA-WYGWVNSDLAEGIDSL-------------	250
Drosophila melanogaster	VCVSEGQAPVLATAVSTKR-WYSWVSSKLTKHR-LSVEK---LEPLDVDT	232
Drosophila erecta	VCVNEGQAPVLATAVSTKR-WYSWVSSKLTKHR-LNVEN---LEPLDVDT	232
Drosophila simulans	VCVSEGQAPVLATAVSTKR-WYSWVSPKLTKHR-LSVEQ---LEPLDVDT	232
Drosophila virilis	VCVSEGSAPVLATAVSTKR-WYCWVSPKLTKHR-LSVPQ---VEPIDVDQ	233
Drosophila willistoni	VCVKAGQGPVLATAVSTER-WYSWVSPKLTKHR-MGVDHMEDMEPCDGDV	257
Drosophila mojavensis	VCVRAGNDPVMATAVSTKR-WYCWVSPKLTKHR-LTVPH---VEPIDVDS	232
Drosophila grimshawi	VCVREGSTPVLATAVSTER-WYCWVSPKLTKHR-LSVPH---VEPIDVAQ	230
Culex quinquefasciatus	VCVPAGAAPVMATALSPTR-WYSWTSPRLIGELPSG-------------	216
Anopheles gambiae	VCVRAGPLPVMATATGTAGCWYSWTSPLLSSGE-AA------------PT	219
Tribolium castaneum	VAVTVGKAPTLATAVSNEA-WYGWVSQMLLDGT-SK-------------	201
Apis mellifera	VCIKEGSLPTLATAVTNKA-WYGWVSKSLIDGV---------------SR	175
Bombus impatiens	VCMKEGPLPTLATAVTNEA-WYGWVSKSLIDGS---------------SR	324
Bombus terrestris	VCMKEGPLPTLATAVSNEA-WYGWVSKSLINGL---------------SR	304
Nasonia vitripennis	NKTDESRFNEINLQMLSKN-LYSQIFESSVDNKEQI-------------	192
Danaus plexippus	VPGPSSCRERIFNSTSLRA-RYGWVSEPLANDNKHE-------------	190
Strongylocentrotus purpuratus	CCMAEGNFPTLAVAVSTNA-WYSWCSHRLIEDH-LT-------------	264
Saccoglossus kowalevskii	VCMTEGKLPTMAVAVSQNA-WYSWCSEQITEDR-FS-------------	222
Branchiostoma floridae	VCMSEGDFPTLATAASPNA-WYSWVSDRLLEDH-YS-------------	207
Ciona intestinalis	VLVSASEAPIMAVAASTDA-WFSWSSSCLKQED----------------	167
Oreochromis niloticus	VCTTEGQCPTLAVAVSPTN-WYSWCSKRLIEER-YS-------------	214
Danio rerio	VCMAEGHCPTLAVALSPTA-WYSWCSHRLVEDR-YA-------------	203
Xenopus tropicalis	VCVSEGHCPTLAAAVSPQY-WYSWCSRRLIEDR-YT-------------	208
Anolis carolinensis	VCMEEGHCPTLAVAASPRA-WYSWCSKRLLEQR-YT-------------	177

```
Meleagris gallopavo            ETFAEIRVQFQQMWGSRSS-TTSTATTQMFHED-AA-------------- 69
Gallus gallus                  --------------------------------------------------
Monodelphis domestica          VCIAEGSCPTLAVAVSPNS-WYSWCSRRLLEER-YS-------------- 290
Mus musculus                   VCLAEGTCPTLAVAISPSA-WYSWCSRRLVEER-YS-------------- 217
Ailuropoda melanoleuca         VCLAEGTCPTLAVAISPSA-WYSWCSRRLVEER-YS-------------- 222
Loxodonta africana             VCLAEGTCPTLAVAISPSA-WYSWCSQRLVEER-YS-------------- 219
Callithrix jacchus             VCLAEGTCPTLAVAISPSA-WYSWCSRRLVEER-YS-------------- 224
Pongo abelii                   VCLAEGICPTLAVAISPSA-WYSWCSRRLVEER-YS-------------- 241
Homo sapiens                   VCLAEGTCPTLAVAISPSA-WYSWCSQRLVEER-YS-------------- 234

Saccharomyces cerevisiae       -------DPAALIPLNTLN------------KEQVIIGHNVAYDRARVLE 236
Amphimedon queenslandica       --EKSKVNFDELIPLEG----------GESWSERIVVGHNVSYDRARIKE 229
Trichoplax adhaerens           LGWKHDNTLKDLIPMEANQKGH--NSQKKNHLKKLIVGHNIGYDRSYIRE 272
Nematostella vectensis         --WIVQPRPDLYIPLETSDG------KETHWRERLVIGHSVGYDRSYVKE 290
Crassostrea gigas              --WTRNLRLCDLIPLESSPEEH--GCHLKKGRKKIVIGHNVGFDRSFVKE 268
Caenorhabditis elegans         --IPEIPTKADLIPIGEIG------------MEKVIIGHNVGFDRARCRE 213
Oscheius tipulae               --YPSCSREEHLISIGDPA------------QSRMIIGHNVGYDRSRVRE 182
Loa loa                        --IPELYRLNHLIAFETDE---------KDLKYRLIMGHNVAFDRSRVRE 224
Bursaphelenchus xylophilus     --VPERCSLQHLIPLEDGSP-----------DPKLVIGHNVGYDRARVRE 213
Dirofilaria immitis            ---------------------------------RLIVGHNVAFDRSRIRE 17
Daphnia pulex                  KNMESIYELKDLINLESKTEQD--RLPLKDQKERIVIGHNVSYDRSRIKE 250
Pediculus humanus corporis     --PTQFYTAEKLIPLGD----------KNKTNPAIVVGHNVSYDRSKIKE 288
Drosophila melanogaster        DSERPHYTTDELIPLGT-------------TGPGLVVGHNVSYDRARLKE 269
Drosophila erecta              DSERPHYTTDELIPLGT-------------SGPGLVVGHNVSYDRARLKE 269
Drosophila simulans            DSERPHYT------------------------------------------ 240
Drosophila virilis             N-ERPHYTLDELIPLGC-------------GEPGLVVGHNVSYDRARLRE 269
Drosophila willistoni          DTERPHYTLDELIPLGS--------------GPGLIVGHNVSYDRARLRD 293
Drosophila mojavensis          N-ERPHYTLDELIPLGH-------------KEPSLVVGHNVSYDRARIRE 268
Drosophila grimshawi           S-DRPHYMLDELIPLGS-------------GAPGLVVGHNVSYDRARLLE 266
Culex quinquefasciatus         --ENHRYQLDEFIPLESTPKDK--QSDGKFSTPRVVVGHNVSYDRARIRE 262
Anopheles gambiae              DQTAADYRLSQLIPLEADT-----PTDERLHRPRVVIGHNASYDRARVRE 264
Tribolium castaneum            PVTTHQYNLDQLIPLESTTKDTGAKLTNHMKKPKIIIGHNVSYDRARVKE 251
Apis mellifera                 NFEGKQYTMDELIPMESIITERGEKLTSFHKKPKIIVGHNVSFDRSKIKE 225
Bombus impatiens               NFEGKQFIIDELIPMESTSSENGEKLTSFHKKPKLIVGHNVSYDRSKIKE 374
Bombus terrestris              NFEGKQFSIDELIPMESTAIENGKKLSSFHKKPKLIVGHNVSYDRSKIKE 354
Nasonia vitripennis            ------------------------DVDKFQQMPKIIIGHNVSFDRVRVKE 218
Danaus plexippus               --HHNSVRYEDLIPLETDGF----EPVGDITRPRMVVGHNVSYDRSKIKE 234
Strongylocentrotus purpuratus  --LSQKLALPDLIPLETKKGST--KPPNKKWTERLIVGHNVSFDRSFVKE 310
Saccoglossus kowalevskii       --WAQKIGLKDLIPLDQQQS-------DGEWLPGVVIGHNVSFDRALVKE 263
Branchiostoma floridae         --WSPHPTLDDLITMETAVRSI--KPGGEDWREKIVIGHNVSFDRSYIKE 253
```

Ciona intestinalis	--PRMEANLESMIPIESAV-----HVKENCTECKLVIGHNVGYDRARIQE	210
Oreochromis niloticus	--WSNQLTLADLIPLETPFNSA--RPPGGKWKERLIVGHNVSFDRAHIKE	260
Danio rerio	--WSSDLSLADLIPLETLANSS--QPRGGEWKERLVVGHNVSFDRAHIKE	249
Xenopus tropicalis	--WSKELSLSDLIPLETSVNCN--CITKNNWTERLVVGHNISFDRAHIKE	254
Anolis carolinensis	--WSSQLTLPDLIPMERGSGFS-----KQDGQERLVVGHNVSFDRAHIKE	220
Meleagris gallopavo	--GGWQLVAVD---VNKPQGRA--PACLQEGGERVVVGHNVAFDRAFIRE	112
Gallus gallus	--	
Monodelphis domestica	--WARSLYLSDLIPLETPAGIS--GPAKQDWSERLIVGHNVCFDRAFIKE	336
Mus musculus	--WTSQLSPADLIPLGGSTSAS--SSTKQDGQEQLVVGHNVSFDRAHIRE	263
Ailuropoda melanoleuca	--WTSQLSPADLIPLEVPASAG--GPTQRGWQEQLVVGHNVSFDRAHIRE	268
Loxodonta africana	--WTSQLSPADLIPLEIPANAG--SPTQRDWQEQLVVGHNVCFDRAHIRE	265
Callithrix jacchus	--WTSQLSPADLIPLEVPAGAS--GPTQRDWQEKLVVGHNVSFDRAHVRE	270
Pongo abelii	--WTSQLSPADLIPLEVPAGAS--SPTQRDWQEQLVVGHNVSFDRAHIRE	287
Homo sapiens	--WTSQLSPADLIPLEVPTGAS--SPTQRDWQEQLVVGHNVSFDRAHIRE	280

Saccharomyces cerevisiae	EYN-FRDSKAFFLDTQSLHIASFGLCSRQRPMFMKNNKKKEAEVESEVHP	285
Amphimedon queenslandica	QYL-FNGPKTKFLDTLSLHTCVSGQTSTQKVLWRSALKRKRQEMESKAFV	278
Trichoplax adhaerens	QYN-IESGNTVFLDTMSLHNCVGGLTSIQRGNWKKMNNGD----------	311
Nematostella vectensis	QYY-IKGPKTYFLDTLSLHMCVSGLTGLQRHLWTAANSGR----------	329
Crassostrea gigas	QYY-IKKSQMRFLDTMSLHIALCGMTSFQQVLYQSSKKST--NRKDVREY	315
Caenorhabditis elegans	AYQSINGSKIRFMDTMSMSIPMYGMADHQQSLYEMYDVET----------	253
Oscheius tipulae	SYS-RQKHKIRYMDTMSMSVSMFGMADHQQIAGQGKVEDEEEVVPKTKEW	231
Loa loa	QYY-RKGTNTRFWDTMSMAIPIYGMADHQVALYEKKDTEV----------	263
Bursaphelenchus xylophilus	QYP-LKESNVKFWDTMSMNTIIQGMADHQRLLYEKKDVED----------	252
Dirofilaria immitis	QYY-RKGTNTRFWDTMSMAIPIYGMADHQVALYEKKDTEV----------	56
Daphnia pulex	QYF-IEGTKVRFFDTMALHIAISGITSFQRNMLHAAKSGTTIDRSLKNPQ	299
Pediculus humanus corporis	QYS-FESNAVRFLDTMAIHTCIGGINKNQTKDLNETSIFN----------	327
Drosophila melanogaster	QYL-TEDTGTRFVDTMSLHMCVSGVTSYQRAMLKSKKEPA----------	308
Drosophila erecta	QYL-IEDTGTRFVDTMSLHMCVSGVTSYQRAMLKSKKEPA----------	308
Drosophila simulans	------------------------LTSYQRAMLKSKKEPA----------	256
Drosophila virilis	QYL-IEDTGTRFVDTMSLHMCVSGITSYQRAMLKSKKEPA----------	308
Drosophila willistoni	QYL-LEDTRTRFVDTMSLHMCVSGVTSYQRAMLKSKKEPA----------	332
Drosophila mojavensis	QYL-IEDTKTRFVDTMSLHICVSGVTSYQRAMLKSKKDPA----------	307
Drosophila grimshawi	QYL-LEDTGTRFVDTMSLHMCVSGVTSYQRAMLKSKKEPA----------	305
Culex quinquefasciatus	QYW-LKSTGLRFVDTMSLHVCVSGVTSYQRAMLKSSKELP----------	301
Anopheles gambiae	QYW-VEPTALRFLDTMSLHVCVSGITSYQRAMLKSSKQLP----------	303
Tribolium castaneum	QYW-LNQTATRFLDTMSLHVCVSGLTSYQRAILKSGKIDE----------	290
Apis mellifera	QYW-LKQTGLRFIDTMSLHICIGGINSYQRSILNSTKNTE----------	264
Bombus impatiens	QYW-LKHTGLRFIDTMSLHICIGGINSYQRTILNSKKDTD----------	413
Bombus terrestris	QYW-LKQTGLRFIDTMSLHICVGGINSYQRTILNSKKDTD----------	393

```
Nasonia vitripennis            QYW-LNPSATKFLDTMALHICISGLSSYQRAILKSTNSKD---------- 257
Danaus plexippus               QYW-LNKTGVRFMDTMSMHICVSGVTSYQRTVLKDKNKEP---------- 273
Strongylocentrotus purpuratus  QYL-LQGTKTRFLDTLSLHMCVSGQTTLQRALWQVNARGG--GATAAPWI 357
Saccoglossus kowalevskii       QYL-MKGPKTRYLDTMSFHMAISGLTSFQRILWNYHKKNKNVGNQDVKEH 312
Branchiostoma floridae         QYF-MKGPKTRFLDTMSLHMAISGLTSFQRILWLASRAGKKQGLKDVREY 302
Ciona intestinalis             QYY-SQLTNVRFLDTMSMHIGVSGFNFQQRVLSQATRSEV---------- 249
Oreochromis niloticus          QYL-LKGSKVRFMDTMSLHMAISGLTGFQRTLWMASKLGKKRGLQEVKEH 309
Danio rerio                    QYL-LKGSKMRFLDTMSLHMAISGLTGFQRSLWMASKYGKRKGLQEVKEH 298
Xenopus tropicalis             QYL-IKGSKMRFMDTMSMHMAISGLTGFQRTLWMASKYGKKKGLQEVKQH 303
Anolis carolinensis            QYM-IQGSRTRFLDTMSMHMAISGLTGFQRSLWMAARQGKRRGQQQVQQH 269
Meleagris gallopavo            QYL-VQGSRVRFLDTMSMHMAISGLTGFQRSLWMAAKHGKRKGLQQVRQH 161
Gallus gallus                  ----MEGSRVRFLDTMSMHMAISGLTGFQRSLWMAAKHGKRKGLQQVRQH 46
Monodelphis domestica          QYL-IQGSRMRFLDTLSMHMAISGLSSFQRSLWIAAKQGKQKGSQMVQKH 385
Mus musculus                   QYL-IQDSRMRFLDTMSMHMAISGLSSFQRSLWMGAKQGKHKTQQSTKRG 312
Ailuropoda melanoleuca         QYL-IQGSRMRFLDTMSMHMAISGLSGFQRSLWMAAKQGKRKARHPTQRG 317
Loxodonta africana             QYL-IQGSRMRFLDTMSMHMAISGLSGFQRSLWMAAKQGKRKARHLTQKG 314
Callithrix jacchus             QYL-IQGSRMRFLDTMSMHMAISGLSSFQRSLWIAAKQGKHKAQCLTKQN 319
Pongo abelii                   QYL-IQGSRMRFLDTMSMHMAISGLSSFQRSLWIAAKQGKHKVQPPTKQV 336
Homo sapiens                   QYL-IQGSRMRFLDTMSMHMAISGLSSFQRSLWIAAKQGKHKVQPPTKQG 329

Saccharomyces cerevisiae       ------------EISIEDYDDPWLNVSALNSLKDVAKFHC---KID---L 317
Amphimedon queenslandica       QSHNEDEFFDAVAKLSRLSKEKWMEVSSPNSLADMYQLYC--GGEK---I 323
Trichoplax adhaerens           ----------HDANGDTNKEAFWAKLSCPSSLADIYTLYT---NNT---L 345
Nematostella vectensis         -----------------KTRQDWMQAGAGNKLVDLYALYC--DGEA---V 357
Crassostrea gigas              MEMKSSRK--------QMVDSYWTNESTMNNLNDVYQHHC--GGRP---L 352
Caenorhabditis elegans         ------------NDSHSDWMNAWKGRVSKNSLVAVHDHLY--PDKA---I 286
Oscheius tipulae               YAK---------QNEKTGWKAAWKSRVTKNGLKDVADFLL---KLP---M 266
Loa loa                        -----------DDSGPVGWIDYWRSLVCKNSLSALHEKLC--GTTSLKPL 300
Bursaphelenchus xylophilus     ------------VLYQIPWLQYWKRRVCRNSLEDVYGKFYPEKSGS---L 287
Dirofilaria immitis            -----------DDSGPVGWVDYWRSLVCKNSLSALHEKLC--GTTSLKPL 93
Daphnia pulex                  YWNGKSRK--------TEENLEWQNVSSFNGLNDVYKLYC--GGLG---L 336
Pediculus humanus corporis     ---------------------DSSEVFCLISLRDMYKLYC---GRE---I 350
Drosophila melanogaster        -----------------AEDLGWLEQSSLNSLVEVHRLYC--GGDT---L 336
Drosophila erecta              -----------------AEDLGWLEQSSLNSLVDVHRLYC--GGDA---L 336
Drosophila simulans            -----------------AEDLGWLEQSSLNSLVEVHRLYC--GGDA---L 284
Drosophila virilis             -----------------EEDLSWLEQSALNNLVDVHRLYC--GGEP---L 336
Drosophila willistoni          -----------------PEDLGWLEQSSLNSLVEVHRLYC--GGEP---L 360
Drosophila mojavensis          -----------------VEDLDWLAQSSLNNLVDVHRLYC--GGEP---L 335
Drosophila grimshawi           -----------------AEDLDWLEQSSLNSLVDVHRLYC--GGEP---L 333
Culex quinquefasciatus         -----------------LEDLSWSSQSSLNNLADVYSLYC--DGAK---L 329
```

Anopheles gambiae	-----------------AEDAGWSEQSSLNNLADVYALYC--GGPP---L	331
Tribolium castaneum	------------------EDEIWKQHSSLNSLNEVHKLYC--GGKS---V	317
Apis mellifera	------------------EKCNLEMQTSLNNLADIHKFYC---GSE---I	290
Bombus impatiens	------------------EKLNLQIQTSLNSLAEIHKFYC---GSE---I	439
Bombus terrestris	------------------EKLNLQIQTSLNSLAEVHKFYC---GSE---I	419
Nasonia vitripennis	------------------EHEHLKSISSLNSLSDIHKFYY---GFN---L	283
Danaus plexippus	----------------HPHDDEWREVSSLNGLSDVHRLYC---GVA---I	301
Strongylocentrotus purpuratus	QKKHNKKS-----FGAPATA--WLAESSPNNLADVYSLYS-DDGST---L	396
Saccoglossus kowalevskii	LKKSQQRL-----QGPLVH--DWVEVSSPNNLGDVYSLYC--DGLA---L	350
Branchiostoma floridae	TSKTRKQF-----KGQGIGGWDWLNVSSINNLGDVHVLYT--GGPK---L	342
Ciona intestinalis	--------GENFVSGSSAFDNDWVSMGTTNALKEVHKFYC---GKT---L	285
Oreochromis niloticus	IKKAGKKH-----EGPMIGSWDWVNISSINNLADVHALYV--GGPP---L	349
Danio rerio	MKRLGRRP-----EGPKIGSWEWVNISSINNLADVHALYV--GGDP---L	338
Xenopus tropicalis	IKKTRSKF-----DGSPISSWDWVNISSINNLADVHALYV--GGPP---L	343
Anolis carolinensis	IKKTRSKT-----GGPAISSWDWVNISSINNLADVHALYV--GGQP---L	309
Meleagris gallopavo	MKKTRSKA-----EGPAVSSWDWVHVSSINNLADVHALYV--GGEP---L	201
Gallus gallus	MKKTRSKA-----EGPAVSSWDWVHVSSINNLADVHALYV--GGEP---L	86
Monodelphis domestica	IKKTQSKA-----KGPLIYAWDWLDISSINNLADVHSLYV--GGPP---L	425
Mus musculus	-QKSPRKA-----NGPAISSWDWMDISSANNLADVHNLYV--GGPP---L	351
Ailuropoda melanoleuca	-QKSPRKA-----SGPVVSSWDWLDISSVNNLADVHGLYV--GGPP---L	356
Loxodonta africana	-QNSQNKG-----RGPAIASWDWLGISSVNNLADVHSLYV--GGPP---L	353
Callithrix jacchus	-QKSQRKA----RGGPVISSWDWLNISSVNSLAEVHSLYV--GGPH---L	359
Pongo abelii	-QKSQRKA----RRGPAVSSWDWLDISSVNNLAEVHRLYV--GGPP---L	376
Homo sapiens	-QKSQRKA----RRGPAISSWDWLDISSVNSLAEVHRLYV--GGPP---L	369

Saccharomyces cerevisiae	D------KTDRDFFASTDKSTIIENFQKLVNYCATDVTATSQVFDEIFPV	361
Amphimedon queenslandica	D------KSLSEIFIKGNSSDIRDNFQDLMGYCYQDVKCTYEILKVLYPL	367
Trichoplax adhaerens	D------KSPVDILIKGSMRDIKDNFQTLMEYCATDVYATHVVLKNVYKL	389
Nematostella vectensis	D------KSLVDVFIHGDMGDVRAQFQDLMAYCAKDVSLTHEVFAALWPF	401
Crassostrea gigas	E------KATRDVFVKGSMADVREMYQELCTYCASDVEATLHVFRKVWPQ	396
Caenorhabditis elegans	NAEGFSKKTMRASFVKDPIEQIREDFQPLMSYCARDNILCAEIYFRLWPE	336
Oscheius tipulae	N------KDTRGYFVKEDIETIREHFQKLMRYCAEDNLATALIFSKMWPM	310
Loa loa	N------KSLQTFFVKEPIDEIRRSFQDLTTYCAYDVVACFELYQVLYPE	344
Bursaphelenchus xylophilus	D------KTYQSVFVTEPIEVIRANFQILIDYCAGDVQACIDVYKKLYPM	331
Dirofilaria immitis	N------KSLQRFFVKEPIDEIRRSFQDLMTYCAYDVIACFELYQVLYPE	137
Daphnia pulex	D------KEKRNIFVTGSLTDIKDDFQQLVTYCARDCQATYRILCVLLPE	380
Pediculus humanus corporis	S------KTERDVFVKGSILDIKNDFENLMSYCSKDTIATFEILQKIFPI	394
Drosophila melanogaster	S------KEPRNIFVEGTLEQVRQSFQSLTNYCASDVEATHRILRVLYPL	380
Drosophila erecta	S------KEPRNIFVEGTLEQVRQSFQSLTNYCASDVEATHRILRVLYPL	380
Drosophila simulans	S------KEPRNIFVEGTLEQVRQSFQSLTNYCASDVEATHRILRVLYPL	328

```
Drosophila virilis            S------KEPRNIFVEGTLEQVRQQFQSLVNYCAGDVEATHRILTKLYPM 380
Drosophila willistoni         S------KEPRNIFVEGTMEQVRQNFQSLVNYCAGDVEATYRILSQLYPL 404
Drosophila mojavensis         S------KEPRNIFVEGTLEQVRQQFQSLVNYCAGDVEATHRILGKLYPL 379
Drosophila grimshawi          S------KEPRNIFVEGTLEQVRQQFQSLVNYCAGDVEATHRILTVLYPL 377
Culex quinquefasciatus        D------KEKRNLFVEGTLPEIRAEFQSLMSYCAGDVAATRNVLTKLFPL 373
Anopheles gambiae             A------KSQRDTFVEGTLQDVRADFDSLMSYCAADVRATGAVLQRLWPL 375
Tribolium castaneum           A------KETRDLFVTGSLNDIREEFQTVMKYCSGDVVATYSVLNELFPM 361
Apis mellifera                N------KNVREVFINGTLKNIKEDFNSLMIYCANDVIATHNVLCKLFPI 334
Bombus impatiens              N------KKFRDTFVDGTLINVKEDFNSLMSYCANDVVATHNVLRQLFPI 483
Bombus terrestris             T------KEFRDTFVNGTLINIKEDFNYLMSYCANDVVATHNVLRQLFPI 463
Nasonia vitripennis           N------KQGRDIFMNGEIQDIKKQFNESMLYCASDVVATHKIFKKMFPI 327
Danaus plexippus              D------KQTRDVFVEGCLEDVQNNFQDLMKYCAGDVIATHNVLRALLPL 345
Strongylocentrotus purpuratus D------KTKRDTFVHGSMADIRADFQSLMTYCAQDVAATHKVISKLLPM 440
Saccoglossus kowalevskii      D------KTERKVFVTGSIQDIRHQFQELMSYCARDVKATHEVFSKILPM 394
Branchiostoma floridae        E------KSERDVFVKGTLQDVRNQFQELMTYCARDVRATHEIFSMQLPM 386
Ciona intestinalis            D------KELRDVFVTGNIKDVRENFQELMWYCAKDVQATYNVLAKLTPL 329
Oreochromis niloticus         Q------KEARETFVKGSMTDVRNNFQELMQYCALDVKATHEVFTEQLPL 393
Danio rerio                   M------KESRELFVKGSMSDIRGNFQELMQYCALDVLATQEVFTQQLPL 382
Xenopus tropicalis            E------KEARELFVKGSISDIRTEFQELMRYCALDVQATHEIFQEQFPL 387
Anolis carolinensis           E------KEARELFVKGSMTDIRNNFQDLMRYCAFDVLATHEVCQEQLPL 353
Meleagris gallopavo           Q------KEARELFVKGTMADVRNNFQELMSYCASDVRATHEVFQEQLPL 245
Gallus gallus                 Q------KEARELFVKGTMADVRNNFQELMSYCASDVRATYEVFQEQLPL 130
Monodelphis domestica         E------KEARELFVKGTMKDIRENFQDLMQYCALDVQATYEVFQEQLPL 469
Mus musculus                  E------KEPRELFVKGSMRDIRENFQDLMQYCARDVWATFEVFQQQLPL 395
Ailuropoda melanoleuca        E------KEPRELFVRGSMKDIRENFQDLMQYCAQDVWATYEVFQQQLPL 400
Loxodonta africana            E------KEPRELFVKGSMKDIRENFQDLMQYCAQDVWATYEVFQQQLPL 397
Callithrix jacchus            E------KEPRELFVKGSMKDIRENFQDLMQYCAQDVWATHEVFQQQLPL 403
Pongo abelii                  E------KEPRELFVKGTMKDIRENFQDLMQYCAQDVWATHEVFQQQLPL 420
Homo sapiens                  E------KEPRELFVKGTMKDIRENFQDLMQYCAQDVWATHEVFQQQLPL 413
                                     *     :       :   ::    **  *      :
Saccharomyces cerevisiae      FLKKCPHPVSFAGLKSLSKCILPTKLNDWNDYLNSSESLYQQSKVQIESK 411
Amphimedon queenslandica      FLHHCPHPVTLAGMLEMSTMYLPVNES-WNTFMQSAS------------- 403
Trichoplax adhaerens          FLLRCPHPVSFAGMLEMSSIFLPVDES-WQYYLNTVEYTYQDLENELNTL 438
Nematostella vectensis        FLERFPHPVTFAGMLEMGSAYLPIDQS-WDSYLRDCNDTYEDLEQEMKGS 450
Crassostrea gigas             FLERFPNPVTLSGMMEMGSAYLPINQN-WERYLNQSDAVYDDLQNELSRL 445
Caenorhabditis elegans        FIKRFPHPATLSGMLNMGNVYLPINSY-WKMFYEKNVQTCEQKKTAATRK 385
Oscheius tipulae              FRQRFPEDITLCGMLTMADAYLPINQH-WRAFSERNYKRSNESKHSSAHA 359
Loa loa                       FRKRFPHPVTWQGMLEIGNVYLPVTKN-WRKFFDLNETRANHENKIAAIG 393
Bursaphelenchus xylophilus    FKERFPSPITSLGMIIVADAYLPVTSN-WRKFFEKCENDADKMNNTSVKA 380
Dirofilaria immitis           FTKRFPHPVTWQGMLEIGNVYLPITKN-WRKFFDNNETRANNENKIAAIG 186
```

```
Daphnia pulex                  FQKRFPHPVTLAGMLEMSTSYLPVNRN-WQRYLQESEDTYRDSENELTQS 429
Pediculus humanus corporis     FLNRFPHPVTLAGMLEMSVAFLPVTSN-WKKFILEADQTYEDLKNEMSLI 443
Drosophila melanogaster        YAERFPHPASLAGMLEMGSAYLPVNSN-WERYIREAQLTYEDLSIEAKYH 429
Drosophila erecta              YADRFPHPASLAGMLEMGSAYLPVNSN-WERYIREAQLTYEDLSIEAKYH 429
Drosophila simulans            YAERFPHPASLAGMLEMGSAYLPVNSN-WERYIREAQLTYEDLSIEAKYH 377
Drosophila virilis             FAARFPHPVTLAGMLEMGSAYLPVNSN-WERYIRDAQLTYEDLSIEAKYH 429
Drosophila willistoni          YAERFPHPASLAGMLEMGSAYLPVNSN-WERYIHESQLTYEDLSIEAKYH 453
Drosophila mojavensis          YAERFPHPVTLAGMLEMGTAYLPVNSN-WERYIRDAQLAYEDLSIEAKYH 428
Drosophila grimshawi           YAERFPHPVTLAGMLEMGSAYLPVNSN-WERYIRDAQLAYEDLNIEAKYH 426
Culex quinquefasciatus         FLQRFPHPATLAGMLEVGNAYLPVNSN-WPRYIQESDLAYEDLDIEAKHL 422
Anopheles gambiae              FRERFPHPATLAGMLEMGSAYLPVNGN-WTRYLTEADLAFEDLDLESKHQ 424
Tribolium castaneum            FLERFPHPVTLAGMLELSTGYLPINSN-WNRYIDNSEQAYEDMDIEGRLL 410
Apis mellifera                 FEERFPHPVTLAGMLELGTAYLPINSN-WKKYLNESETTFEDLNYETKIV 383
Bombus impatiens               FKKRFPHPVTLAGMLEIGTAYLPINSN-WQKYLNESETTFEDLNYETKVI 532
Bombus terrestris              FEERFPHPVTLAGMLEIGTAYLPINSN-WQKYLNESETTFEDLNYETKVI 512
Nasonia vitripennis            FIKRFPHPATLAGMLELSTTYLPVNYN-WKRYLEEAETTYKDLNFENKVC 376
Danaus plexippus               FLERFPHPVTFAGMLELGSAYLPVNSN-WSQYLDSADTVFEDLKLESQQI 394
Strongylocentrotus purpuratus  FSDRFPHPVTFAAMLEMGQAYLPINQN-WERYLRDAQETFDNLQQEMKQS 489
Saccoglossus kowalevskii       YFERFPNPVTFAAMLEMSQVYLPVNNN-WQRYLNETQGTYEDLQKEMKLS 443
Branchiostoma floridae         FLERFPHAVTLAGMLEMGQCYLPVNSN-WERYLRDAEDTFQELQKEMKLS 435
Ciona intestinalis             FFDRFPHPVTFAGMLEMGQMYLPVNNN-WFRYIRSCQDQYNSLNLELEKH 378
Oreochromis niloticus          FMERCPHPVTLAGMLEMGVSYLPINQN-WGRYLEDSQDVYEELQREMKKS 442
Danio rerio                    FMERCPHPVTLAGMLEMGVCYLPVNHN-WRRYLEDAQATYEELQREMKKS 431
Xenopus tropicalis             FMERCPHPVTLSGMLEMGVSYLPVNQN-WERYLDEAQTSYEELQKEMKKS 436
Anolis carolinensis            FLERCPHPVTFAGMLEMGVSYLPVNQN-WERYLEEAQLTFEELQKEMKKS 402
Meleagris gallopavo            FMERCPHPVTFAGMLEMGVSYLPVNSN-WRRYLDDAQGTYEELQKEMKKS 294
Gallus gallus                  FMERCPHPVTFAGMLEMGVSYLPVNSN-WRRYLDDAQGTYEELQKEMKKS 179
Monodelphis domestica          FMERCPHPVTLAGMLEMGVSYLPVNQN-WERYLVEAQSTYEELQREMKKS 518
Mus musculus                   FLERCPHPVTLAGMLEMGVSYLPVNQN-WERYLTEAQNTYEELQREMKKS 444
Ailuropoda melanoleuca         FLERCPHPVTLAGMLEMGVSYLPVNQN-WERYLVEAQSTYEELQREMKKS 449
Loxodonta africana             FLERCPHPVTLAGMLEMGVPYLPVNQN-WERYLAEAHSTYEELQREMKKS 446
Callithrix jacchus             FLERCPHPVTLAGMLEMGVSYLPVNQN-WERYLVEAQGTYEELQREMKKS 452
Pongo abelii                   FLERCPHPVTLAGMLEMGVSYLPVNQN-WERYLSEAQGTYEELQREMKKS 469
Homo sapiens                   FLERCPHPVTLAGMLEMGVSYLPVNQN-WERYLAEAQGTYEELQREMKKS 462
                               :  : *   :  .:  :.   **     *  :
Saccharomyces cerevisiae       IVQIIKDIVLLKDKPDF----YLKDPWLSQLDWTTKPLRL---------- 447
Amphimedon queenslandica       --------------------------------------------------
Trichoplax adhaerens           LKHLADDACHLLRN-DR----FRDDPWLWSLNWNIKRNSLKSLSNI---- 479
Nematostella vectensis         LMHLADEACNYHH--DR----YSRDPWLWSLDWSVQDVRTKVPKKP---- 490
Crassostrea gigas              LQKLANEACELLHD-KR----YEEDLWLWDLDWSVNTYKLNKPKKK---- 486
```

```
Caenorhabditis elegans         IIESARLVAKRLDDEGEEIGPEKNDVWMWHHDWTFNQKQ----------- 424
Oscheius tipulae               TISSAQELIK---------------------------------------- 369
Loa loa                        VVYAAKELVEKLEKPIQS---YKHDPWMWSVDWSIRKGE----------- 429
Bursaphelenchus xylophilus     VVTSARALVEELENENK----FEQDPWLWVSDWQCKYKTI---------- 416
Dirofilaria immitis            VIYAARELMEKLEKPNES---YKSDPWMWSVDWSSHGQE----------- 222
Daphnia pulex                  LKREADRASHMMTD-KS----YEKDPWLWDLDWSTKPLKVKKTSVP---- 470
Pediculus humanus corporis     LSRKSDLACRLFHN-DE----FKKDLWLWDENWSIQNLKLTK-------- 480
Drosophila melanogaster        LGRRAEEACSLLLD-DQ----YRQNLWLWDEDWSVQELKLKQPP------ 468
Drosophila erecta              LGRRAEEACSLLLD-DQ----YRQNLWLWDEDWSVQELKLKQPP------ 468
Drosophila simulans            LGRRAEEACSLLLD-DQ----YRQNLWLWDEDWSVQELKLKQPP------ 416
Drosophila virilis             LGRRAEEACALLHD-EQ----YRQHLWLWDEDWSVQSLKLKQLP------ 468
Drosophila willistoni          LGRRAEEACSLLHD-EQ----YKKHLWLWDEDWSVQSLKLKKAP------ 492
Drosophila mojavensis          LGRRAEEACALLHD-EK----YRKNLWLWDEDWSVQALKLKQMP------ 467
Drosophila grimshawi           LGRRAEEACALLHD-EQ----YRQHLWLWDEDWSVQQLKLKQLP------ 465
Culex quinquefasciatus         LAQRADAACRLLHD-AE----FRKDLWLWDQDWSVQELKLKVAKKVGRK- 466
Anopheles gambiae              LAQRAEAACGLLHD-AA----YRRDLWLWDQDWSVQELKLKAAKVEGRK- 468
Tribolium castaneum            LAKRAEQACQLLHD-DK----YKEDLWLWDEDWTVKNLKIKKTSPK---- 451
Apis mellifera                 LTKRANAVCKLLHN-KK----YKEDLWMWDEDWSTQMVKVKTQSLKKNLK 428
Bombus impatiens               LAKRANAVCELMHN-EK----YKEDLWMWDEDWSTQTFKVKAQYSKTKIK 577
Bombus terrestris              LAKRANAVCELMHN-EK----YKEDLWMWDEDWSTHTLKVKAQYSKTKIR 557
Nasonia vitripennis            LAKRADEVCRLSIS-SQ----YKNDPWMWDEDWSIKNMNLKKGYDK---- 417
Danaus plexippus               LSSKADEACRMMEN-EG----YKEDPWMWDQDWSVQKLKLKKNATK---- 435
Strongylocentrotus purpuratus  LMHIANDACNMHHQ-DK----YREDPWLWELDWSVDDYRLRKTIPL---- 530
Saccoglossus kowalevskii       LMHLANDSCELLHN-DR----YKKDPWLWELDWSSTELKMKKIKGK---- 484
Branchiostoma floridae         LMHLANDACQLMHN-DR----YQEDPWLWELDWSTQELTLKKETSA---- 476
Ciona intestinalis             LTGLASDACHYAVN-EK----YKTDKWLCDLDWTTRPYKLLTKPRKGWV- 422
Oreochromis niloticus          LMILADDACQLLEN-DR----YKEDPWLWDLEWDVQEFKQKKVAAS---- 483
Danio rerio                    LMILADDACQLLQD-DR----YKDDPWLWDLDWDVQEFKQKKVPVS---- 472
Xenopus tropicalis             LMNLANDACQLLTE-DA----YKEDPWLWDLEWDIQEFKQKKTKIS---- 477
Anolis carolinensis            LMNLADGACQLLHG-DR----YKDDPWLWDLEWDLQEFKQKKQKKKENW- 446
Meleagris gallopavo            LMNLANDACQLLHE-DR----YKEDPWLWDLEWDTQEFKQKKPPK----- 334
Gallus gallus                  LMNLANDACQLLHE-DR----YKEDPWLWDLEWDTQEFKQKKPAK----- 219
Monodelphis domestica          LMDLANDACHLVSG-DR----YKDNPWLWDLEWDLQEFKLKKKKI----- 558
Mus musculus                   LMDLANDACQLLSG-ER----YKEDPWLWDLEWDLQEFKQKKAKKV---- 485
Ailuropoda melanoleuca         LMDLANDACQLLSG-QR----YKEDPWLWDLEWDLQEFKQKKAKKV---- 490
Loxodonta africana             LMDLANDACQLLSG-ER----YKEDPWLWDLEWDLQEFKQKKAKKV---- 487
Callithrix jacchus             LMDLANDACQLLSG-ER----YKEDPWLWDLEWDLQEFKQKKARKV---- 493
Pongo abelii                   LMDLANDACQLLSG-ER----YKEDPWLWDLEWDLQEFKQKKAKKV---- 510
Homo sapiens                   LMDLANDACQLLSG-ER----YKEDPWLWDLEWDLQEFKQKKAKKV---- 503
```

Saccharomyces cerevisiae	--	
Amphimedon queenslandica	--	
Trichoplax adhaerens	----------SKKLLKTITG------------------------------	489
Nematostella vectensis	----------KKPRKKKTSA------------------------------	500
Crassostrea gigas	----------PKDTNKEKTP------------------------------	496
Caenorhabditis elegans	--	
Oscheius tipulae	--	
Loa loa	--	
Bursaphelenchus xylophilus	--	
Dirofilaria immitis	--	
Daphnia pulex	----------KKKKSAAVIP------------------------------	480
Pediculus humanus corporis	-----------KKVPNNNET------------------------------	489
Drosophila melanogaster	---------KRKPLPTVELK------------------------------	479
Drosophila erecta	---------KRKPLPTVEVK------------------------------	479
Drosophila simulans	---------KRKPLPTVELK------------------------------	427
Drosophila virilis	---------KRKQLPTVHLL------------------------------	479
Drosophila willistoni	---------KRKPLPKMDEE------------------------------	503
Drosophila mojavensis	---------KRKQLSTVQLP------------------------------	478
Drosophila grimshawi	---------KRKQLPTVPLQ------------------------------	476
Culex quinquefasciatus	----------KKKGEVGEIS------------------------------	476
Anopheles gambiae	---------GKEPGSKAEEP------------------------------	479
Tribolium castaneum	----------TKQIETPPPQ------------------------------	461
Apis mellifera	NISQIQNAKRKKETKTQSYL------------------------------	448
Bombus impatiens	QVCKAQKTEEKKKTETQSYL------------------------------	597
Bombus terrestris	QVCKTRQTKEKKKTETQSYL------------------------------	577
Nasonia vitripennis	-------------IWKITTI------------------------------	424
Danaus plexippus	----------KKDTIESKKPASEDVNRISSKFELLSDEYV----------	465
Strongylocentrotus purpuratus	----------SERAKKKKKD------------EECSKEKVE---------	549
Saccoglossus kowalevskii	----------SKNGNNTQTDVGEVTNDGLSQKSQSMVSAVDMKDNEDDCN	524
Branchiostoma floridae	----------KKKKIKVEKT------------DEGEDQRVKPERKSTKKK	504
Ciona intestinalis	-----AK---EKPDLKVKSY------------------------------	434
Oreochromis niloticus	----------KKKGSKKADNK--------------QIATPLPDWE-----	504
Danio rerio	----------KKKAKQEAEAT------------EAANDSEAIDWP-----	495
Xenopus tropicalis	----------KKQKRLKEAQ-------------ESVGNKFIEDLN-----	499
Anolis carolinensis	----------RKKDQGNHEDF-----------PKVVEKASTMECQ-----	470
Meleagris gallopavo	----------RKKDQKINSET--------------SETGSAQEWQ-----	355
Gallus gallus	----------RKKDQKINSEA-----------SETGRWAGGLQPSVL---	245
Monodelphis domestica	----------RKNDQNISCRS-----------PAKAKNSSEMEWQ-----	582
Mus musculus	----------KK--PASASKL----------PIEGAGPFGDPMDQ-----	508
Ailuropoda melanoleuca	----------RKKEPTAASKL----------PIEGAGAPGDPKDQ-----	515

```
Loxodonta africana            ----------KKKEPATASKL----------PTTGAGAPGDPKDQ----- 512
Callithrix jacchus            ----------K-KEPATASKL----------PIEGPGAPGDPMDQ----- 517
Pongo abelii                  ----------K-KEPATASKL----------PIEGAGAPGDPMDQ----- 534
Homo sapiens                  ----------K-KEPATASKL----------PIEGAGAPGDPMDQ----- 527

Saccharomyces cerevisiae      --------------------------------------------------
Amphimedon queenslandica      --------------------------------------------------
Trichoplax adhaerens          --------------------------------------------------
Nematostella vectensis        --------------------------------------------------
Crassostrea gigas             --------------------------------------------------
Caenorhabditis elegans        --------------------------------------------------
Oscheius tipulae              --------------------------------------------------
Loa loa                       --------------------------------------------------
Bursaphelenchus xylophilus    --------------------------------------------------
Dirofilaria immitis           --------------------------------------------------
Daphnia pulex                 --------------------------------------------------
Pediculus humanus corporis    --------------------------------------------------
Drosophila melanogaster       --------------------------------------------------
Drosophila erecta             --------------------------------------------------
Drosophila simulans           --------------------------------------------------
Drosophila virilis            --------------------------------------------------
Drosophila willistoni         --------------------------------------------------
Drosophila mojavensis         --------------------------------------------------
Drosophila grimshawi          --------------------------------------------------
Culex quinquefasciatus        --------------------------------------------------
Anopheles gambiae             --------------------------------------------------
Tribolium castaneum           --------------------------------------------------
Apis mellifera                --------------------------------------------------
Bombus impatiens              --------------------------------------------------
Bombus terrestris             --------------------------------------------------
Nasonia vitripennis           --------------------------------------------------
Danaus plexippus              --------------------------------------------------
Strongylocentrotus purpuratus --------------------------------------------------
Saccoglossus kowalevskii      HALNFKEAPYGQSAESVRSSDEFEFDENSAYEEYAENSFLSTGNECDQTI 574
Branchiostoma floridae        KKK----------------------------------------------- 507
Ciona intestinalis            --------------------------------------------------
Oreochromis niloticus         EDP----------------------------------------------- 507
Danio rerio                   EDP----------------------------------------------- 498
Xenopus tropicalis            EDT----------------------------------------------- 502
Anolis carolinensis           EDP----------------------------------------------- 473
```

```
Meleagris gallopavo            EDP----------------------------------------------- 358
Gallus gallus                  SDP----------------------------------------------- 248
Monodelphis domestica          EDP----------------------------------------------- 585
Mus musculus                   EDP----------------------------------------------- 511
Ailuropoda melanoleuca         EDP----------------------------------------------- 518
Loxodonta africana             EDP----------------------------------------------- 515
Callithrix jacchus             EDL----------------------------------------------- 520
Pongo abelii                   EDL----------------------------------------------- 537
Homo sapiens                   EDL----------------------------------------------- 530

Saccharomyces cerevisiae       --------------------------------------------------
Amphimedon queenslandica       --------------------------------------------------
Trichoplax adhaerens           ---------------------------------------------ELTEN 494
Nematostella vectensis         ----------------SAATITLDRMESDSLECPSHNELI--------HN 526
Crassostrea gigas              --------------------------------------------------
Caenorhabditis elegans         --------------------------------------------------
Oscheius tipulae               --------------------------------------------------
Loa loa                        --------------------------------------------------
Bursaphelenchus xylophilus     --------------------------------------------------
Dirofilaria immitis            --------------------------------------------------
Daphnia pulex                  ---------------------------SLNNGESENQTNKEDNDDVILEI 503
Pediculus humanus corporis     ------------------------------------------NVKVTAKE 497
Drosophila melanogaster        -------------------------------------DSGNT-----PEE 487
Drosophila erecta              -------------------------------------YAVNT-----PEE 487
Drosophila simulans            -------------------------------------DSGNT-----PEE 435
Drosophila virilis             -------------------------------------EQELN-----PEQ 487
Drosophila willistoni          -----------------------------------------------CSS 506
Drosophila mojavensis          -------------------------------------EQGLD-----VEQ 486
Drosophila grimshawi           ------------------------------------DERQLN-----AEQ 485
Culex quinquefasciatus         ------------------------------------EEAGGSVDEQVDEE 490
Anopheles gambiae              -------------------------------------LAGGD------EQ 486
Tribolium castaneum            -------------------------------------SPDDE------AT 468
Apis mellifera                 -------------------------------------TDDED------EE 455
Bombus impatiens               -------------------------------------TDDED-----EEE 605
Bombus terrestris              -------------------------------------TDDED-----EEE 585
Nasonia vitripennis            ------------------------------------------IPVTVIVK 432
Danaus plexippus               -------------------------------------DSLKTNEKVTHDF 478
Strongylocentrotus purpuratus  ----------------------------------SEEEEN--------EE 557
Saccoglossus kowalevskii       ESLPFNTQKRSYSQDASVSKPTFKTSELVAIARKRKREDE--------KR 616
Branchiostoma floridae         -------------------------------GKKEEAEEAE------DQE 520
```

```
Ciona intestinalis            -----------------------------LLPLDYNTEELKFLGKSIRKE 455
Oreochromis niloticus         -------------------------------GPPSEEEME--------GP 518
Danio rerio                   -------------------------------GPPLEEEEG--------DS 509
Xenopus tropicalis            -------------------------------GPPSEEEES--------RP 513
Anolis carolinensis           -------------------------------GPPTEEEEQ--------GI 484
Meleagris gallopavo           -------------------------------GPPSEEEEL--------KA 369
Gallus gallus                 -------------------------------GPPSEEEEL--------RA 259
Monodelphis domestica         -------------------------------GPPIEEEELQ-------SD 597
Mus musculus                  -------------------------------GPPSEEEELQ-------RS 523
Ailuropoda melanoleuca        -------------------------------GPPS-EEEVQ-------RD 529
Loxodonta africana            -------------------------------GPPSEEEESE-------RD 527
Callithrix jacchus            -------------------------------GPPSEEEEFQ-------QD 532
Pongo abelii                  -------------------------------GPCSEEEEFQ-------QD 549
Homo sapiens                  -------------------------------GPCSEEEEFQ-------QD 542

Saccharomyces cerevisiae      -------------TKKGVPAKCQKLPGFPEWYRQLFPSKD---------- 474
Amphimedon queenslandica      --------------------------------------------------
Trichoplax adhaerens          IDDKILEERLKLQKERLHAKRGSQKSGYPDWYADLCPPLY-------IKS 537
Nematostella vectensis        EDKEELLSAIP-PAKEIMYKRRQHLPGYP-WYRKLCPKNG---------- 564
Crassostrea gigas             ---DEILEDIL-ASKDRIPKVSRHMVGYPAWYRELCPRTS---------- 532
Caenorhabditis elegans        --------------------------SNFEWFNKLFKARS---------- 438
Oscheius tipulae              -------------------------------YRK---------------- 372
Loa loa                       --------------------------KFPIWYESLLRTKN---------- 443
Bursaphelenchus xylophilus    --------------------------ARPAWYVNLFNKKS---------- 430
Dirofilaria immitis           --------------------------KFPVWYESLLRTRN---------- 236
Daphnia pulex                 QKLKCQFQELF-NTKELLFKRNSFLPGYPNWYSSLCDRLQ---------- 542
Pediculus humanus corporis    DALNEKFQYLF-DLGKILPKNKPFLPGYPMWYRKLCEKTD---------- 536
Drosophila melanogaster       RRLQAKFQHLY-DQQALLPARRPLLPGYPLWYRKLCRKPPAKRADEILE- 535
Drosophila erecta             RRLQAKFQHLY-DQQALLPARRPLLPGYPLWYRKLCRKPPTKRSDEILE- 535
Drosophila simulans           RRLQAKFQHLY-DQQALLPARRPLLPGYPLWYRKLCRKPPAKRADEILE- 483
Drosophila virilis            RRLQRKFQHLY-DQRALLPARRPLLPGYPQWYRKLCRKPPTCYTDDQPEE 536
Drosophila willistoni         SPFQAKFQHLY-DQRALLPARRPLLPGYPQWYRKLCQKPPLQTSERDLQ- 554
Drosophila mojavensis         LRLQRKFQYLY-NQRALLPSRRPLLPGYPQWYRKLCRKPPPDYTED---- 531
Drosophila grimshawi          RRLLQKFQHLY-DQRALLPARRPLLPGYPQWYRKLCQKPPPDYSEEELQ- 533
Culex quinquefasciatus        DALAAKFQYLY-ETKALLPVRRPLLPGYPAWYRNLCSKPT---------- 529
Anopheles gambiae             TRLANKFAHLY-ATASRLPVRRPLLPGYPAWYRALCSKPT---------- 525
Tribolium castaneum           DPLEEKFRDLM-ATKANISRVNSHLPGYPTWYRKLSTKPD---------- 507
Apis mellifera                DPLEKKFAYLT-ETRKFLPLKLPHMSGYPAWYRKLCFKIN---------- 494
Bombus impatiens              DPLEKEFGYLM-ETRKFLPSKLRHMPGYPAWYRKLCPKVN---------- 644
Bombus terrestris             DPLEKEFGYLM-ETRKFLPSKLRHMPGYPAWYRKLCPKVN---------- 624
```

```
Nasonia vitripennis             TILNKQFRYLH-KTENFLPKKLPHMPGYPNWYRKLCKKNK---------- 471
Danaus plexippus                ENLNKKFKYLY-DLGDLLPVKRPFLAGYPAWYRKLCTKPG---------- 517
Strongylocentrotus purpuratus   VKEETCVEDVL-STAARIPKVNQHMTGYPVWFRELCPKKS---------- 596
Saccoglossus kowalevskii        LEAENRINDIL-ASKNLLYKRKQHMVGYPNWYRDLCPRFK---------- 655
Branchiostoma floridae          ETEEDRIQRVL-ETADRLPKIKRHMPGYPAWYRDLCPKVT---------- 559
Ciona intestinalis              RKKLDLIEKLN-ETANRLPLVKPHMPGAPKWYSELCVPEK---------- 494
Oreochromis niloticus           CPSRLAVENLK-ETVNRLPKRRQHLPAHPGWYRKLCEKMS---------- 557
Danio rerio                     DPRRELLQRLK-ETVSCLPKRRQHLPAHPSWYRKLCVKMS---------- 548
Xenopus tropicalis              SLAKIYLEDLKLKTLPLLPKRSQHLPGHPGWYRKLCPKLE---------- 553
Anolis carolinensis             PESRLSLNRLK-ETVALQPKRIQHLPGHPGWYRKLCPRLD---------- 523
Meleagris gallopavo             SESHTCLERLK-ETVTLQPKRLQHLPGHPGWYRKLCLRLE---------- 408
Gallus gallus                   PESSTCLERLK-ETITLQPKRLQHLPGHPGWYRKLCPRLE---------- 298
Monodelphis domestica           SAIRTRMEELK-ETAILLPKRCQHLPGHPGWYRKLCPRLN---------- 636
Mus musculus                    VTAHNRLQQLR-STTDLLPKRPQHLPGHPGWYRKLCPRLD---------- 562
Ailuropoda melanoleuca          GMARACLEHLK-GTAEVLPKRPQHLPGHPGWYRKLCPRLD---------- 568
Loxodonta africana              AAARACLQQLK-GTIELLPKRPQHLPGHPGWYRKLCPRLD---------- 566
Callithrix jacchus              VASRACLQKLK-GTTELLPKRPQHLPGHPGWYRKLCPRLD---------- 571
Pongo abelii                    VMARACLQKLK-ETTELLPKRPQHLPGHPGWYRKLCPRLD---------- 588
Homo sapiens                    VMARACLQKLK-GTTELLPKRPQHLPGHPGWYRKLCPRLD---------- 581

Saccharomyces cerevisiae        --------TVEP-KITIKSRIIPILFKLSWENSPVIWSKESGWCFNVPHE 515
Amphimedon queenslandica        --------------------------------------------------
Trichoplax adhaerens            ELN-SKWRSSPV-NISPTKRIVPLLLRLAYNNNPVYYDKKYGWGYLVLRQ 585
Nematostella vectensis          --------EGAS-LVSPQCQTAPLLLRMTWDGFPLHYSRKHGWGYLVPGR 605
Crassostrea gigas               --D-PDWKPGPS-LVSTSRRITPKLLRMTYKGYPLHYDDKYGWGYLIPDK 578
Caenorhabditis elegans          FVNLSLDEVDSH-HIALKSHLIPAIFGFVFGPFPLFKTRSKGWGFLVPNW 487
Oscheius tipulae                -----------E-HVVAKSTVIPSIYGLVYHSYPLYKTKKAGWGYLAPRN 410
Loa loa                         LVHTPVEELSQA-DVKLKSRVVPRLFGLCWGPYPLHYKTDKGWGFLVPKD 492
Bursaphelenchus xylophilus      LVDSNVDELHSD-HVKLKSSDVPRIFGLCYGPYPIFHKRDYGWGFLVPAA 479
Dirofilaria immitis             LIYMPVEKLSQA-DVKLKSRVVPRLFGLCWGPYPLHYKTDKGWGFLIPKD 285
Daphnia pulex                   -----DGLIGPT-EVSTSAQVVPKLLKLTWDGYPLFYSRELGWGYLVPGR 586
Pediculus humanus corporis      ---------SNV-HLTTSMLVTPKLLNLTWENFPLYHSKKHGWGILVADI 576
Drosophila melanogaster         DDE-EPWSPGAS-EISTGMQIAPKLLSLCWEGYPLHYEREQGWGFLVPFR 583
Drosophila erecta               DDE-EPWFPGAS-EISTGMQIAPKLLSLCWEGYPLHYEREQGWGFLVPFR 583
Drosophila simulans             DDE-EPWFPGAS-EISTGMQIAPKLLSLCWEGYPLHFEREQGWGFLVPFR 531
Drosophila virilis              ANV-EPWTPGAC-DISTGMQIAPKLLSLCWEGYPLHYQREHGWGFLVPFR 584
Drosophila willistoni           -SE-EEWTPGAT-GISTGMQIAPKLLSLCWEGYPLHYLKEHGWGFLVPFR 601
Drosophila mojavensis           VNE-EPWTPGAC-DISTGMQLAAKLLSLCWESYPLHYLREHGWGFLVPFR 579
Drosophila grimshawi            QQD-EPWTPGAS-GISTGMQIAPKLLSLCWEGYPLHYQKGHGWGFLVPFR 581
Culex quinquefasciatus          --T-EDWSPGPT-EIGTGMQIAPKLLRLCWEGYPLHYIRGQGWGFLVPHK 575
```

Anopheles gambiae	--A-GDWCPGPSGKLGTGMQIAPKLLSLCWEGYPLHYIRGHGWGFLVPFK	572
Tribolium castaneum	--S-PDWVPGPH-LISTSTRITPKLLSLTWEGYPLHYTREKGWGFLIPFT	553
Apis mellifera	--D-ENWSPGPQ-NISISKQIVPKLLNLTWENFPLHYIKNKGWGILVPHT	540
Bombus impatiens	--D-ENWSPGPQ-NISTSKHIVPKLLNLTWECYPLHYVKDKGWGILVPHT	690
Bombus terrestris	--D-ENWSPGPQ-NISTSKHVVPKLLNLTWECYPLHYVKDKGWGILVPHT	670
Nasonia vitripennis	----INVFPRTQ-QLSTSMLITPKLLNLTWEQYPLYHIKNHGWGFLVKDL	516
Danaus plexippus	--KDPDWRAGAN-NITTSMQITPKLLRLSWEGYPLHHLQSEGWGFLVPYS	564
Strongylocentrotus purpuratus	--H-VDWSPGPS-LISSQTRITPKLLRLTWDGYPLHYTIKHGWGYLVPGK	642
Saccoglossus kowalevskii	-VD-SDWKPGPS-LITSQVQVTPKLLRLTWDGFPLYRDDKHGWGYLVPGR	702
Branchiostoma floridae	--G--AWSPGPN-LISAQARVTPKLLRLRWDGFPLHYEASQGWGYLVPGR	604
Ciona intestinalis	--D-QNWKPGAT-LLSTLTRVTPRLLRLMWQGCPLYHVKDYGWGYLCPDQ	540
Oreochromis niloticus	--D-NSWSPGAS-LISLQMRLTPKLMGLTWDGFPLHYTEKHGWGYLVPGR	603
Danio rerio	--EAEDWSPGAS-LISLQMRITPKLMGLTWDGFPLHYTDQHGWGYLVPGR	595
Xenopus tropicalis	--D-PDWLPGPS-LISLQMRLTPKLMRLTWDGFPLHYSEKHGWGYLVPGK	599
Anolis carolinensis	--A-PSWVPGPS-LISLQMRVTPKLMRLTWDGFPLHYSEKYGWGYLVPGR	569
Meleagris gallopavo	--E-EGWVPGPS-LISLQMRVTPKLMRLAWDGFPLHYSEKHGWGYLVPGR	454
Gallus gallus	--E-EGWVPGPS-LISLQMRVTPKLMRLAWDGFPLHYSEKHGWGYLVPGR	344
Monodelphis domestica	--D-PDWIPGPS-LISLQMRVTPKLMMLTWDGFPVHYSEKHGWGYLVPGR	682
Mus musculus	--D-PAWAPGPS-LLSLQMRVTPKLMALTWDGFPLHYSDSHGWGYLVPGR	608
Ailuropoda melanoleuca	--D-PAWTPGPS-LLSLQMRVTPKLMAMTWDGFPLHYSERHGWGYLVPGR	614
Loxodonta africana	--D-PAWTPGPS-LLSLQMRVTPKLMALTWDGFPLHYSEQHGWGYLVPGR	612
Callithrix jacchus	--D-PAWTPGPS-LLSLQMRVTPKLMALTWDGFPLHYSERHGWGYLVPGR	617
Pongo abelii	--D-PAWTPGPS-LLSLQMRVTPKLMALTWDGFPLHYSERHGWGYLVPGR	634
Homo sapiens	--D-PAWTPGPS-LLSLQMRVTPKLMALTWDGFPLHYSERHGWGYLVPGR	627

Saccharomyces cerevisiae	QVETYK--	521
Amphimedon queenslandica	--	
Trichoplax adhaerens	DAIADE--------TLYGTSHKEKCGNGDTDEQKLQFPVRSW-LKILKER	626
Nematostella vectensis	TDNLHP-------------RDPGEETAEKLERPGPDTPFPTVFQSLWQQH	642
Crassostrea gigas	HKAASD-------RQKLAMA----------EEGMSKFPIANL-----FKM	606
Caenorhabditis elegans	KPVEKA-------LQDPNFHEIDAKIDFKPDSAVKKFPLRSF--------	522
Oscheius tipulae	RK------------------------------KKGKIP------------	418
Loa loa	SRIALS--------DVPEMEEVILRRR-----VKVTIPVKAI--------	521
Bursaphelenchus xylophilus	GIFSITFVDSCCLEEVPDVVEVPLQRR-----ETVKFPARRI--------	516
Dirofilaria immitis	SYAALS--------DVPEMEEVVLRRG-----VKATIPVKAI--------	314
Daphnia pulex	PLNLSR---------------------IDEDVCEIPFPLKDA-LALFPPR	614
Pediculus humanus corporis	FA----------------------------ENVNTLLPFEKL--------	590
Drosophila melanogaster	SD----------------------------SEGVDRLPMEQL-----LAH	600
Drosophila erecta	SD----------------------------SEGVDRLPINQL-----LAR	600
Drosophila simulans	SD----------------------------SEGVDRLPMDQL-----LAR	548

Drosophila virilis	SVE---------------------------CSGNSKLPIEQL-----LTR	602
Drosophila willistoni	ND----------------------------ARESQALPLEQL-----LQR	618
Drosophila mojavensis	SSQ---------------------------SNRSTNIPIEQL-----LGR	597
Drosophila grimshawi	SGD---------------------------NSRQTDLPIEQL-----LER	599
Culex quinquefasciatus	YRRDEE------------------------DEDGGQIPLEQL-----VAA	596
Anopheles gambiae	YARDEEF-----------------------EADGAALPLEQL-----VAA	594
Tribolium castaneum	DD----------------------------TETSRKLPLAKL-----LEK	570
Apis mellifera	ND----------------------------LNIKTKIPLKQL-----LAQ	557
Bombus impatiens	NN----------------------------LDIKTKIPLRQL-----LAH	707
Bombus terrestris	NN----------------------------LDIETKVPLRQL-----LAH	687
Nasonia vitripennis	NKCDIK-------------------------------P------------	523
Danaus plexippus	RHIAE-------------------------DAKEPLVPLEKI-----LEI	584
Strongylocentrotus purpuratus	PDSRYQ-------QRTEP---------------GPSFPLNLQ-DMMSSRQ	669
Saccoglossus kowalevskii	QHNMSR-------DKQDATSQGDA---ESVDKKMVEFPIRSL-RKLMKDI	741
Branchiostoma floridae	TDNMNG-------TDKQD--------------GERNFPTKAV-LDAYKEY	632
Ciona intestinalis	TAP----------------------------------PVEKK--------	548
Oreochromis niloticus	RDNLDS-------QE---------------ENTGLVCPHRAI-ESVYKEY	630
Danio rerio	RDNLDI-------SE---------------DNEEPVCPYRAI-ESVYKEY	622
Xenopus tropicalis	KDNILN-------NEQEE---------------TILCPYRAI-EDIFSEY	626
Anolis carolinensis	KDNLAE-------PGPGD--------------PLPTCPFRVI-EHLYREH	597
Meleagris gallopavo	QDNLPA-------ASAEP--------------EGPVCPHRAI-EQLYRQH	482
Gallus gallus	QDNLPA-------ASAEP--------------EGPVCPHRAI-ERLYRQH	372
Monodelphis domestica	QDNLLV-------DSSET--------------DVAACPYRAI-EALYQKY	710
Mus musculus	RDNLTE-------PPVSPT----------VESAAVTCPYRAI-ESLYRKH	640
Ailuropoda melanoleuca	RDNLAQ-------APAETA----------PASAGVACPYRAI-ESLYRKH	646
Loxodonta africana	RDNLAK-------VPVGTA----------LASAGVACPYRAI-ESLYRKH	644
Callithrix jacchus	RDNLAK-------LLAGPT----------LESAGVVCPYRAI-ESLYRKH	649
Pongo abelii	RDNLAN-------LPTGTT----------LESAGVVCPYRAI-ESLYRKH	666
Homo sapiens	RDNLAK-------LPTGTT----------LESAGVVCPYRAI-ESLYRKH	659

Saccharomyces cerevisiae	-------------------------------AKNYVLADSV---------	531
Amphimedon queenslandica	--	
Trichoplax adhaerens	KNNSHDGQACIKALDT----------------SQVDYFNPI---------	651
Nematostella vectensis	RMNPLTSSPSPSPLDDEELDIRHL--------TDSDMWSEY---------	675
Crassostrea gigas	CNESVSEGLLVPMSDKELESMLNTA-------KDDMEWTERAKHLDEAGI	649
Caenorhabditis elegans	-------------------------------------YETV---------	526
Oscheius tipulae	--	
Loa loa	-------------------------------------LSVI---------	525
Bursaphelenchus xylophilus	-------------------------------------FDLI---------	520
Dirofilaria immitis	-------------------------------------LSVI---------	318

Daphnia pulex	PADQSVLTKGIITAEEAMLKLRQMTDLTADPVDLAMQWQAT---------	655
Pediculus humanus corporis	------KEITLKSFDIPLDFYK----------TDNLNYKNC---------	615
Drosophila melanogaster	CPVPEFARLSASKAESD-MAFDMLPGQVEQHLGKREHYKKL---------	640
Drosophila erecta	CPVPEFARQSASKAESD-LAFDMLPGQVEEHLGKREHYKKL---------	640
Drosophila simulans	CPVPEFARLSASKAESD-MAFDMLPGQVEQHLGKREHYKKL---------	588
Drosophila virilis	CAIPEFARQYAAGEDGE-LAMDMLPKQIDEHLSKRQFYKKI---------	642
Drosophila willistoni	CPVPEFARHCASANESE-LALDALPRNLDEHLGRREYFKKI---------	658
Drosophila mojavensis	CAIPEFARQFAVGEAVD-LAMDQLPKQVDEHLSKRHFYKKI---------	637
Drosophila grimshawi	CAIPEFARLYAAAENGD-MAMDMLPGQVEEQLAKRQFFKKI---------	639
Culex quinquefasciatus	CPVLE-INPAASADESS-EALEGLWRDVEATISRKDYYRKL---------	635
Anopheles gambiae	CPVVEPKHAAATPAEST-EALAQLAKHVEQTISRKDYYRRK---------	634
Tribolium castaneum	CPLSK---INTKEHDSS-DTFANITQIVQDNLMRKEYYSKI---------	607
Apis mellifera	CALPK---KEYLSDEICDDTMSTMNKELEKDLHKTEFWYNK---------	595
Bombus impatiens	CLFSK---MKDLVVETI-YMMSTINKEVQNNLHKTEFWRNK---------	744
Bombus terrestris	CSFSK---MEDLVDETM-YTTLTINKEVQNNLHKTEFWRDK---------	724
Nasonia vitripennis	--------------------------------SDCELLSQT---------	532
Danaus plexippus	CPLMT------SKADYVESELHMLPQNVEEDLSRRAYYARK---------	619
Strongylocentrotus purpuratus	EKQSSTQSD---	678
Saccoglossus kowalevskii	--------PKFSDSEKEDDLM-----------TEDEILSKI-EELGN---	768
Branchiostoma floridae	LKQRRDTRD---KEPLDQRYSG---------WANDDMWDHI-MDLSQ---	666
Ciona intestinalis	---------------VEDEDLLKE--------VDQESWNKQ---------	566
Oreochromis niloticus	CEQHSKERPDYLDSIPSDDLML---------TD-STVWTKV-EELSSLES	669
Danio rerio	CEQKGKEQPKYPDSPVSDELML---------TD-TAVWQTV-EEVSRLEM	661
Xenopus tropicalis	SKNKAKDRRVSEHSSLHEDFIL---------TDDSSMWQKV-EQLSRMEM	666
Anolis carolinensis	CKERGKEEPISLDTSLEEEHML---------TESSTIWQKV-EELSRMEM	637
Meleagris gallopavo	CLQRGQEQHPE-EVGVEDELMV---------LEGSSMWQKV-RGCGVWDE	521
Gallus gallus	CLQRGQEQPPE-EAGVEDELMV---------LEGSSMWQKV-EELSQLEL	411
Monodelphis domestica	CQETGKEQLVFQ--PLPEEFML---------TD-STVWQTV-EELSRLEP	747
Mus musculus	CLEQGKQQLEPQEVDLAEEFLL---------TDSSAMWQTV-EELGCLDV	680
Ailuropoda melanoleuca	CLEQGKQHPEPQQAGLAEEFLL---------TDGSTMWQTV-EELGCLEV	686
Loxodonta africana	CLEQGKQQPEPQEAGLAEEFLL---------TDNSTMWETV-EELGCLEL	684
Callithrix jacchus	CLEQGKQQLTPQEASLAEEFLF---------TDNSTIWQTL-EELDYLEV	689
Pongo abelii	CLEQGKQQLIPQEAGLAEEFLL---------TDNSAIWQTV-EELDYLEV	706
Homo sapiens	CLEQGKQQLMPQEAGLAEEFLL---------TDNSAIWQTV-EELDYLEV	699
Saccharomyces cerevisiae	--------------------------------------SQEE-----EEI	538
Amphimedon queenslandica	--	
Trichoplax adhaerens	--------------------------------------DVEL-----EPR	658
Nematostella vectensis	----EYQEIKANGKQQGLEGGSPRQEGCESGL------QGWR-----HSK	710
Crassostrea gigas	KKQAK---------------------------------KKDD----ILEV	662

Caenorhabditis elegans	--------------------------------------QNNVYMLGELPI	538
Oscheius tipulae	--	
Loa loa	--------------------------------------QQNI----AEGI	533
Bursaphelenchus xylophilus	--------------------------------------EKNK----KKEF	528
Dirofilaria immitis	--------------------------------------QQNI----AEGV	326
Daphnia pulex	-------------------RSSLNNDADPANLHPAIDKSKEKHTYLTSDR	686
Pediculus humanus corporis	--------------------------------------ETKN----KLVP	623
Drosophila melanogaster	--------------------------------------SQK-----QQRL	647
Drosophila erecta	--------------------------------------SQK-----QQRL	647
Drosophila simulans	--------------------------------------SQK-----QQRL	595
Drosophila virilis	--------------------------------------SQR-----QQQL	649
Drosophila willistoni	--------------------------------------SKR-----QQKL	665
Drosophila mojavensis	--------------------------------------SQR-----QLNL	644
Drosophila grimshawi	--------------------------------------SQR-----QQRL	646
Culex quinquefasciatus	--------------------------------------KQD-------GN	640
Anopheles gambiae	--------------------------------------ASD-----ASRA	641
Tribolium castaneum	--------------------------------------RKD---------	610
Apis mellifera	--------------------------------------KKNP----NIQS	603
Bombus impatiens	--------------------------------------KEKP----VAEL	752
Bombus terrestris	--------------------------------------NEKP----VPEL	732
Nasonia vitripennis	--------------------------------------TAKK--------	536
Danaus plexippus	--------------------------------------KKEE-----QAV	626
Strongylocentrotus purpuratus	--------------------DVPSDSRTNESS------AKTS-----RSA	697
Saccoglossus kowalevskii	----------------GNGSNWDSEEMLKMTK------KRLC-----RDS	791
Branchiostoma floridae	--KAK---------GEGNGEMEEAPIYDSSVL------YSNG-----ESA	694
Ciona intestinalis	--------------------------------------EVQS----NHTP	574
Oreochromis niloticus	LMEEN-----G---LRMRKSAEKNKNRSTDPH------YMKE-----ASH	700
Danio rerio	FDDEA----------LSTAAPSKRITKKVNSV------FKSL-----NGE	690
Xenopus tropicalis	DLSAE---------VPSTGTMKKRNNKPEPPA------EPEF-----CSL	696
Anolis carolinensis	EMDVD---------------PMSNQLRQDE--------MGFA-----QSH	659
Meleagris gallopavo	EIPHN---------------QGWGWLFELPRS------LMEE-----SSQ	545
Gallus gallus	DMERPGRAEQSQITVPGLTSRCHLQDEDGLPE------LVEE-----SSQ	450
Monodelphis domestica	DADFE------------TDRATQEET------------NSLQ-----LSQ	768
Mus musculus	EAEAK---------MEN---SGLSQPLVLPAA------CAPK-----SSQ	707
Ailuropoda melanoleuca	EAEVEAEAEAR---AECCGEAVPGQPLALTAA------GGPK-----ASQ	722
Loxodonta africana	EAEAK---------MENCQAAVPGQPSVLVSR------TAPS----TVSQ	715
Callithrix jacchus	EAEAK---------MENLRAAVPGQPLALTAP------GGPK-----DSQ	719
Pongo abelii	EAEAK---------MENLRAPVPGQPLALTAR------GGPK-----DTQ	736
Homo sapiens	EAEAK---------MENLRAAVPGQPLALTAR------GGPK-----DTQ	729

Saccharomyces cerevisiae	RTH-----------NLGLQCTGVLFKVPHPNGPTFNCTNLLTKSYNH-FF	576
Amphimedon queenslandica	--	
Trichoplax adhaerens	RNLSQGDKLLGPY-TIDIPG-VDFYKIPHKDGSHLRCGSPLSKDYLK-QY	705
Nematostella vectensis	PPSYHGV---GPFNDVDLPG-VWFFRIPHKNGDQYRCGNPLAKDYIT-KL	755
Crassostrea gigas	KETKY---------DIGIPG-CWFQKLPHKDGKGSNVGNPLSKDFLP-LI	701
Caenorhabditis elegans	SPDRKGF-------ELDEAGLVRFYQLDHPSGEG-NVGDPLTKHFAK-DL	579
Oscheius tipulae	----------------------NFYQ-----SGFGNVGDVISKSFYK-QF	440
Loa loa	GDVVLTH-------SHSSISIFGFHKLPHPNGEHDNVGDPISKAFRL-EI	575
Bursaphelenchus xylophilus	PDVMVPL-----RPDSSFAG-FNFYRLPHPKGEGQNVGNPFNKEYINCFF	572
Dirofilaria immitis	GDVVRTH-------SHSSISIFDFHKLPHPSGDNGNVGDPISKAFHL-EI	368
Daphnia pulex	PAWHVGI---GPY-DVGVVG-CWFFRLPHKSGVDNNVGNPLAKDYLN-KI	730
Pediculus humanus corporis	PFYYKGN---GIWCNHKIDDCCYFFKLPHKDGPLKNVGNPLSLNFTK-IM	669
Drosophila melanogaster	ETQYQGS---GVWCNKVLDDCCFFLKLPHKNGPSFRVGNPLSKDFLN-KF	693
Drosophila erecta	ETQYQGS---GVWCNKVLDDCCFFLKLPHKNGPSFRVGNPLSKDFLN-KF	693
Drosophila simulans	ETQYQGS---GVWCNKVLDDCCFFLKLPHKNGPSFRVGNPLSKDFLN-KF	641
Drosophila virilis	EQNYKGS---GVWCNQVLDDCCFFLKLPHKNGPSFRVGNPLSKDFLN-KF	695
Drosophila willistoni	ETQYKGS---GVWCNHILENSCFFLKLPHKNGPSYRVGNPLSRDFLN-KF	711
Drosophila mojavensis	EKQYKGS---GVWCNHVIDDCCFFLKLPHKNGPSFRVGNPLSKDFLN-KF	690
Drosophila grimshawi	EEKYKGS---GVWCNQVLEDCCFFLKLPHKNGPSFRVGNPLSKDFLN-KF	692
Culex quinquefasciatus	KNVYKGT---GIWCNLDLEECCYFLKLPHKDGPSHRVGNPLSKDFLN-KF	686
Anopheles gambiae	PAPYTGS---GVWCNVELEGCCYFLKLPHKDGGGHRVGNPLSKDFLA-KF	687
Tribolium castaneum	----KTS---GIWCSTEIEDCCYFLKLPHKNGAAYNVGNPLAKDFLN-KF	652
Apis mellifera	TDIYKGS---AHWCDTNIDNCCYFFKLPHKDGKNYNVGNPLSKHFLN-KF	649
Bombus impatiens	HNIYKGS---AFWCNVEIDNCCYFLKLPHKDGKSNNVGNPLSKDFLN-KF	798
Bombus terrestris	YNIYKGS---AFWCNVDIDNCCYFLKLPHKDGKDNNVGNPLSRDFLN-KF	778
Nasonia vitripennis	----------LHLPDNKIEN--FFFKLPHKNGILFNVGSPLSKDFLN-NF	573
Danaus plexippus	ANQYHGL---GVWCGVQIQGCCHFLRLPHKDGPKYKVGNPLARDFLN-MF	672
Strongylocentrotus purpuratus	PS-HSGR---LPVKDVNIPG-CWFYKLPHKDGADSNVGNPLAKDFLN-KV	741
Saccoglossus kowalevskii	PSWHVGN---GPHNDVDIPG-CWFFKMPHKDGSHKKVGNPLAKDFLS-KM	836
Branchiostoma floridae	PEFHRGA---GPYNDVDLPG-VWFFRLPHKDGAKFKVGNPLAKDYLN-KM	739
Ciona intestinalis	AENIEG-----------LPDGYTFHRLPHAEGAGKNVGSPLSRDFIS-HV	612
Oreochromis niloticus	CHYHHGN---GPYNDVDIPG-CWFFKLPHKDGNHNNVGSPFSKDFLA-KM	745
Danio rerio	CPYHHGN---GPYNDVNIPG-CWFFKLPHKDGNENNVGSPFSKDFLS-KM	735
Xenopus tropicalis	PDYHHGN---SPCSDVNVSG-CWFYKLPHKDGYANNVGSPFAKDFLP-KM	741
Anolis carolinensis	PEYHHGN---GPYNEVNIPG-CWFFRLPHKDGSDNNVGSPFAKDFLS-KM	704
Meleagris gallopavo	PTFHHGN---GPYNDVNVPG-CWFFKLPHK--------------------	571
Gallus gallus	PSFHHGN---GPYNDVNIPG-CWFFKLPHKDGNENNVGSPFAKDFLP-RM	495
Monodelphis domestica	PAYHHGN---GPYNDVNIPG-CWFFKLPHKDGNNYNVGSPFAKDFLP-KM	813
Mus musculus	PTYHHGN---GPYNDVNIPG-CWFFKLPHKDGNNYNVGSPFAKDFLP-KM	752
Ailuropoda melanoleuca	PAYHHGN---GPYNDVDVPG-CWFFKLPHKDGSSCNVGSPFAKDFLP-KM	767

Loxodonta africana	PTFHHGN---GPYNDVDVPG-CWFFKLPHKDGNSCNVGSPFAKDFLP-MM	760
Callithrix jacchus	PTYHHGN---GPYNDVDVPG-CWFFKLPHKDGNNYNVGSPFAKDFLP-KM	764
Pongo abelii	PSYHHGN---GPYNDVDIPG-CWFFKLPHKDGNSCNVGSPFAKDFLP-KM	781
Homo sapiens	PSYHHGN---GPYNDVDIPG-CWFFKLPHKDGNSCNVGSPFAKDFLP-KM	774
Saccharomyces cerevisiae	EKGVLKSES---ELAHQALQINSSGSYWMSARERIQSQFVVPSCKFPNEF	623
Amphimedon queenslandica	-----------------------------------NQFVV---WTNEES	414
Trichoplax adhaerens	EDGTITSF--GSDIAKRTVELKLMLSYWKNAHDRVRNQFVV---YYKHDE	750
Nematostella vectensis	DDKTLVSA--GDRSARRTLVTNKMISFWRNAHKRI-SQMAV---WFGADD	799
Crassostrea gigas	ETGVLKSLL---ITAENVLKLTKMCSYWKNNRDRISGQMAL---TLEEED	745
Caenorhabditis elegans	KELVLRPTR-YEEYFDVVLDSIQTTQFWTSYSDRYCSEVAI---WSPEAT	625
Oscheius tipulae	AEGTFRPTR-FEQNFDQIIDTSKITKHWTSYNKRYEEEIDV---YY----	482
Loa loa	EEGVLRPMR-YKKEFLDLYRARNTTRFWNNYRDRFQEQVTV---WF----	617
Bursaphelenchus xylophilus	KEGILRPTR-FEAECREFIDAGSVTRFWGNYRERYNEQVTC---WL----	614
Dirofilaria immitis	EEGVLWPMR-FKKKFSDLYRARNTTRFWNNYRDRFQEQVTI---WL----	410
Daphnia pulex	EDGTLKATS--SSIAEHILKLNRNIIYWRNARDRIMSQMVV---WLRQSQ	775
Pediculus humanus corporis	ASNFLCGSD---GTAKKVIDISTKITYWRNNRDRIKNQMVV---WMNENE	713
Drosophila melanogaster	AENVLSSGDPSCQAAARVIDIARMMSYWRNNRDRIMGQMVV---WLDSQQ	740
Drosophila erecta	SENVLSSGDPSCQAAARVIDIARMMSYWRNNRDRILGQMVV---WLNPQQ	740
Drosophila simulans	AENVLSSGDPSCQAAARVIDIARMMSYWRNNRDRIMGQMVV---WLDPQQ	688
Drosophila virilis	SEHALSSGDPTCQAAARVIEIARMMSYWRNNQDRILGQMVV---WLQAEE	742
Drosophila willistoni	SENVLSSGNPSCQAAARVIEIARMMSYWRNNRDRIMNQMVV---WLQREC	758
Drosophila mojavensis	SEHALSSGDPSCQAAARVIEIARMMSYWRNNRDRILGQMVV---WLHSEH	737
Drosophila grimshawi	SENALSSGDPTCQAASRVIEIARMMSYWRNNRDRILSQMVI---WLPAQQ	739
Culex quinquefasciatus	SENVLAGDG---KTAERVVEIARMLSYWRNNRDRITGQLVG---WLGKES	730
Anopheles gambiae	SENVLAGDG---RAAERVVEIARMLSYWRNNRDRIRGQLVV---WCRP--	729
Tribolium castaneum	SENVLAGDT---QSAEQVLAIARTLWYWRNNRDRIFDQMVV---WCDGAQ	696
Apis mellifera	SENILGSSD---RDAVEILKIARMVSYWRNNRDRIMSQFTI---WFEKSY	693
Bombus impatiens	SENVLASSD---ASAAEILKIERMISYWRNNRDRIMSQFAV---WFENSY	842
Bombus terrestris	SENVLASSD---ASAVEILKIARMVSYWRNNRDRIMSQFAV---WFENSY	822
Nasonia vitripennis	SKNILDSIT---TSTSRIIEISKMSSYWKNNRDRILSQLVI---WLDSKQ	617
Danaus plexippus	SQNVLSAQG---NEAEKV--------------------------------	687
Strongylocentrotus purpuratus	EDGTLGSG--GGAQANRILEINKFISFWRNAQKRISSQMVV---HLEGGD	786
Saccoglossus kowalevskii	EDGTLSSF--GGVQANRILEISKMISFWRNAQDRISSQMVI---SLERSE	881
Branchiostoma floridae	EDGTLTAAA-GGP-ANRTLEVNKMVSFWRNARDRVMSQMVV---WLKKKE	784
Ciona intestinalis	ESGLLSSYN--PLDALRCLEINKITSYWRSSQKRVLNQIVV---EDANDK	657
Oreochromis niloticus	EDGTLRAGR-GGTNATRALEINKMMSFWRNAHKRISSQMVL---WLRKGE	791
Danio rerio	ESGTLQAGR-EGTNATRALEINKMISFWRNAQKRISSQMVV---WLRRAE	781
Xenopus tropicalis	EDGTLQASI-GDSSATRALEINKMISFWRNAHKRISSQMVV---WMKKGE	787
Anolis carolinensis	EDGTLKAGI-GATDGTQALEINKKISFWRNAHKRISFQMVV---WLKKGE	750

Meleagris gallopavo	--	
Gallus gallus	EDGTLRATV-GRTHGTRALEINKMVSFWRNAHKRVSSQVVV---WLKKGE	541
Monodelphis domestica	EDGTLQAGP-GGASGPRALEINKMVSFWRNAHKRISSQMVV---WLQKGE	859
Mus musculus	EDGTLQAGP-GGASGPRALEINKMISFWRNAHKRISSQMVV---WLPRSA	798
Ailuropoda melanoleuca	EDGTLQAGP-GGASGPRALEINKMISFWRNAHKRISSQMVV---WLPRSA	813
Loxodonta africana	EDGTLQAGP-GGASGPRALEINKMISFWRNAHKRISSQMVV---WLPRSA	806
Callithrix jacchus	EDGTLQAGP-GGASGPRALEINKMISFWRNAHKRISSQMVV---WLPRSA	810
Pongo abelii	EDGTLQAGP-GGASGPRALEINKMISFWRNAHKRISSQMVV---WLPRSA	827
Homo sapiens	EDGTLQAGP-GGASGPRALEINKMISFWRNAHKRISSQMVV---WLPRSA	820

Saccharomyces cerevisiae	QSLSAKSSLNNEKTNDLAIIIPKIVPMGTITRRAVENAWLTASNAKAN-R	672
Amphimedon queenslandica	ASD--------HKRKAQGVIIPKVQVSGTVTRRAVEPTWLTASNAKIN-K	455
Trichoplax adhaerens	IADSIRNHSQFKPLQKYGAILPPVAPAGTVTRRAVEATWLTASNAKAN-R	799
Nematostella vectensis	LPDQV-KSAQFSSHNTYGAIVPRVISAGTVTRRAVEPTWLTASNPRED-R	847
Crassostrea gigas	LPSTVTSDPDYHNDGMYGSIVPRVVPAGTITRRAVEKTWMTASNAYVD-R	794
Caenorhabditis elegans	WRTAE------NLEMIDGAIAAAVVPAGTISRRSVHKLWVTLTNQSTGHV	669
Oscheius tipulae	-----------DAEKTRGAIAPSIRPAGTVTRRSSHKLWVTLSNDSDEVT	521
Loa loa	-----------DENGDEGAIAPSIIPAGTVTRRAVHKLWLTAINAKDDQM	656
Bursaphelenchus xylophilus	-----------DDDCNVGAIAPAIVPSGTITRRAVHKLWLTSANAKEG-M	652
Dirofilaria immitis	-----------DENGDEGAIAPSIIPAGTVTRRAVHKLWLTAINPKDDQM	449
Daphnia pulex	IPRNVTKSEHFDPLNTYGAILPQIVTSGTLTRRAVESTWLTASNAYKD-R	824
Pediculus humanus corporis	SN----YPDLLNEDTSVGIIIPMVVVAGTVTRRAVERTWLTASKVSEN-R	758
Drosophila melanogaster	LPNEFTGEK--CQPIAYGAICPQVVACGTLTRRAMEPTWMTASNSRPD-R	787
Drosophila erecta	LPNELT-----GQPIAYGAICPQVVACGTLTRRAMEPTWMTASNSRTD-R	784
Drosophila simulans	LPDELTVEK--SQPIAYGAICPQVVACGTLTRRAMEPTWMTASNSRPD-R	735
Drosophila virilis	LPSELRIH---AKRMAYGAIVPQIVVAGTLTRRAMESTWMTASNSRAN-R	788
Drosophila willistoni	LPSGLTSH---LQHTSYGAICPQVVACGTLTRRAMEPTWMTASNSRAD-R	804
Drosophila mojavensis	LPGDLQAH---AQRTSYGAILPQVVVAGTLTRRAMESTWMTASNSRAN-R	783
Drosophila grimshawi	LPADLRSH---AERTAYGAICPQVVVAGTLTRRAMESTWMTASNSRAN-R	785
Culex quinquefasciatus	LPRELR-----DEEMEFGAIIPQVVVCGTLTRRAMEPTWMTASNAQRE-R	774
Anopheles gambiae	-----------GEPDPIGAIIPQVVVCGTLTRRAMEPTWMTASNAQRE-R	767
Tribolium castaneum	RGAAL------DDGAQRGAIIPQVVVCGTLTRRAVEPTWMTASNARPD-R	739
Apis mellifera	LSCSIKSS---KKSMCYGAILPLVAVCGTLTRRAVEPTWMTASNSDTE-R	739
Bombus impatiens	LPSSVRNS---RKSMRYGAILPIVVVCGTLTRRAVEPTWMTASNVHPE-R	888
Bombus terrestris	LPSTVRNS---KKSMRYGAILPIVAVCGTLTRRAVEPTWMTASNAHPE-R	868
Nasonia vitripennis	NVNSKNS----TNYINFGAIIPQVIVSGTLTRRAVEPTWMTASNAHCE-R	662
Danaus plexippus	--	
Strongylocentrotus purpuratus	LPLSVRRNPDYDEDGFYGAIIPRVITAGTVTRRAVEPTWLTASNARTD-R	835
Saccoglossus kowalevskii	LPKTVTRHVEYDEDNNYGAILPKVVTAGTVTRRMVEPTWLTASNARPD-R	930
Branchiostoma floridae	LPRIVSRNPDYEEEGSYGAIIPRVVTAGTVTRRAVEPTWLTASNARPD-R	833

Ciona intestinalis	R----------RDYMAYSAIVPQAIVCGAVSRRAVERVWLTASNPQLD-R	696
Oreochromis niloticus	LPRSVSRHKDFDEEGQYGAILPQVITAGTVTRRAVEPTWLTASNARRD-R	840
Danio rerio	LPRSVRLHQDYDEEGQYGAILPQVIPAGTVTRRAVEPTWLTASNARKD-R	830
Xenopus tropicalis	LPRTITRDPEYDEENKYGCILPLVVSAGTITRRAVEPTWLTASNARAD-R	836
Anolis carolinensis	LPRSVTRHPDYDEESRYGAILPQVITAGTVTRRAVEPTWLTASNARAD-R	799
Meleagris gallopavo	--AD-R	574
Gallus gallus	LPRAVTRHPAYSEEEDYGAILPQVVTAGTITRRAVEPTWLTASNARAD-R	590
Monodelphis domestica	LPRTVIRHKDYDEECYYGAILPQVVTAGTITRRAVEPTWLTASNARVD-R	908
Mus musculus	LPRVVTRHPSFDEEGHYGAILPQVVTAGTITRRAVEPTWLTASNARPD-R	847
Ailuropoda melanoleuca	LPRAVTRHPDYDEEGRYGAILPQVVTAGTITRRAVEPTWLTASNARPD-R	862
Loxodonta africana	LPRAVTRHPDYDEEGHYGAILPQVVTAGTITRRAVEPTWLTASNARPD-R	855
Callithrix jacchus	LPRAVIRHPDYDEEGLYGAILPQVVTAGTITRRAVEPTWLTASNARPD-R	859
Pongo abelii	LPRAVIRHPDYDEEGLYGAILPQVVTAGTITRRAVEPTWLTASNARPD-R	876
Homo sapiens	LPRAVIRHPDYDEEGLYGAILPQVVTAGTITRRAVEPTWLTASNARPD-R	869

Saccharomyces cerevisiae	IGSELKTQVKAPPGYCFVGADVDSEELWIASLVGDSIF--------NVHG	714
Amphimedon queenslandica	IGSEQKAFVQAPPGYCIVGADVDSQEVWIASLLGDNHFT-------GLQG	498
Trichoplax adhaerens	VGSELKAMIRAPPGYHFVGADVDSQELWIAALYADARFG-------RTHG	842
Nematostella vectensis	VGSELKSLVRAPPGYSLVGADVDSQELWIAAVLGDANFA-------GIH-	889
Crassostrea gigas	VGSEMKSMIQSPPGYHFVGADVDSQELWIAAVIGDSKH--------RIHG	836
Caenorhabditis elegans	IGTGIKAMVQAPAGYRLVGADVDSQEQWLAALYGDASAEKRLPLEKRVAG	719
Oscheius tipulae	IGKGIKNMIQAPRGYKIVGADVDSQEQWLAAVFGDSSVAKIFPEEERYTG	571
Loa loa	IGTNLKSMVECPQDWHIVGADVDSQEQWIAAMLGDCCVGR------GIAG	700
Bursaphelenchus xylophilus	LGSDLKSMVQCSPGWNLVGADVDSQEQWLAALFGDFLEGK------QKAG	696
Dirofilaria immitis	IGTNLKSMVECPQDWHIVGADVDSQEQWIAALLGDCCVGR------GVAG	493
Daphnia pulex	IGSELKAMIQCPPGFNFVGADVDSQELWIASIIGDSFFA-------GQHG	867
Pediculus humanus corporis	IGSELRALVQAPSGYHIVGADVDSQELWIAALLGDSTYA-------KVHG	801
Drosophila melanogaster	LGSELRSMVQAPPGYRLVGADVDSQELWIASVLGDAYAC-------GEHG	830
Drosophila erecta	LGSELRSMVQAPPGYRLVGADVDSQELWIASVLGDAYAC-------GEHG	827
Drosophila simulans	LGSELRSMVQAPPGYRLVGADVDSQELWIASVLGDAYAC-------GEHG	778
Drosophila virilis	IGSELRSMVQAPPGYKLVGADVDSQELWIASVLGDAYAH-------GEHG	831
Drosophila willistoni	LGSELRAMVQAPPGYRLVGADVDSQELWIASVLGDAYAW-------GEHG	847
Drosophila mojavensis	IGSELRSMVQAPPGYKLVGADVDSQELWIASVLGDAYAH-------GEHG	826
Drosophila grimshawi	IGSELRAMVQAPPGYKLVGADVDSQELWIASVLGDAYAH-------GEHG	828
Culex quinquefasciatus	IGSELRAMVQAPRGFKLVGADVDSQELWIASVLGDGHAA-------AIHG	817
Anopheles gambiae	VGSELRAMVQAPPGYRLVGADVDSQELWIASVLGDAGT--------GLHG	809
Tribolium castaneum	VGSELRAMVQAPPGYNIIGADVDSQELWIASIIGDADRA-------RLHG	782
Apis mellifera	VGSELRAMIQAPPGYNIVGADVDSQELWISSIIGDAYYK-------KIHG	782
Bombus impatiens	IGSELRAMVQAPPGYYIVGADVDSQELWISSIIGDAYYK-------KIHG	931
Bombus terrestris	VGSELRAMVQAPPGYCIVGADVDSQELWISSIIGDAYYK-------KIHG	911

Nasonia vitripennis	IGSELRGIINAPSGYSIIGADVDSQELWIASIIGDAFKKDI-----KIHG	707
Danaus plexippus	--	
Strongylocentrotus purpuratus	VGSELKSMIQCPPGYHFVGADVDSQELWIAALVGDKNFA-------GFHG	878
Saccoglossus kowalevskii	VGSELKAMVQAPPGYHFVGADVDSQELWIASLLGDARFA-------GFHG	973
Branchiostoma floridae	VGSELKAIVQAPPGYHFIGADVDSQELWIAAILGDANFA-------GFHG	876
Ciona intestinalis	IGSELKAMVQAPPGFCFVGADVDSQELWIASLLGDASSA-------KEHG	739
Oreochromis niloticus	VGSELKAMVQVPPGYHLIGADVDSQELWIAAVLGEAHFA-------GMHG	883
Danio rerio	VGSELKAMVQVPPAYHLVGADVDSQELWIAAMLGEAHFA-------GMHG	873
Xenopus tropicalis	VGSELKAMVQVPPGYHIIGADVDSQELWIAAILGEAHFA-------GIHG	879
Anolis carolinensis	VGSELKAMVQAPPGYHLVGADVDSQELWIAAVLGEAHFA-------GLHG	842
Meleagris gallopavo	VGSELKAMVQVPPGYSLVGADVDSQELWIAAVLGEAHFA-------GMHG	617
Gallus gallus	VGSELKAMVQVPPGYSLVGADVDSQELWIAAVLGEAHFA-------GMHG	633
Monodelphis domestica	VGSELKAMVQAPPGYVLVGADVDSQELWIAAVLGDAHFA-------RIHG	951
Mus musculus	VGSELKAMVQAPPGYVLVGADVDSQELWIAAVLGDAHFA-------GMHG	890
Ailuropoda melanoleuca	VGSELKAMVQAPPGYVLVGADVDSQELWIAAVLGDAHFA-------RMHG	905
Loxodonta africana	VGSELKAMVQAPPGYVLVGADVDSQELWIAAVLGDAHFA-------GMHG	898
Callithrix jacchus	VGSELKAMVQAPPGYTLVGADVDSQELWIAAVLGDAHFA-------GMHG	902
Pongo abelii	VGSELKAMVQAPPGYTLVGADVDSQELWIAAVLGDAHFA-------GMHG	919
Homo sapiens	VGSELKAMVQAPPGYTLVGADVDSQELWIAAVLGDAHFA-------GMHG	912

Saccharomyces cerevisiae	GTAIGWMC-LEGTKNEGTDLHTKTAQILGCSRNEAKIFNYGRIYGAGAKF	763
Amphimedon queenslandica	GTAFGWMS-LQGNKSEGTDIHSKTAQTIGITRDHAKVFNYSRIYGSGKQF	547
Trichoplax adhaerens	CTAIGWMT-LQGKKSQGTDLHSKTAETVGISRDQAKTFNYGRIYGAGLNY	891
Nematostella vectensis	CTAFGWMT-LQGQKSDGTDLHSKTADTIGISRDHAKCLTTGVFTARGRSS	938
Crassostrea gigas	STPFGWMT-LQGNKTDKTDLHSKTAETAGISRDHAKILNYGRIYGAGQKF	885
Caenorhabditis elegans	KTAFSNMM-LAGSKSDNTDLHSVVASQLKISRNHAKVLNYARLYGSGETH	768
Oscheius tipulae	LTPFSNMM-LAGSKSDGSDLHSVVARQLKINRHDAKILNYARLYGSGQAH	620
Loa loa	ATPFSNML-LAGRKADNSDLHSVVAKEVGISRDKAKVLNYARLYGSGVAH	749
Bursaphelenchus xylophilus	STAFSNMQ-LAGTKSTGTDLHTVVAESVGISRNNAKVLNYARLYGSGMNH	745
Dirofilaria immitis	ATPFSNML-LAGRKVDHSDLHSVIAKEVGISRDKAKILNYARLYGSGVIH	542
Daphnia pulex	STAFGWMT-LQGKKSDGTDMHSRTAAAVEITRDQAKILNYGRIYGAGERF	916
Pediculus humanus corporis	CTPLGWMT-LQGTKSNESDMHSRTAKAIGISRDHAKILNYARIYGAGVKF	850
Drosophila melanogaster	ATPLGWMT-LSGSKSNGSDMHSITAKAVGISRDHAKVINYARIYGAGQLF	879
Drosophila erecta	ATPLGWMT-LSGSKSNGSDMHSITAKAVGISRDHAKVINYARIYGAGQLF	876
Drosophila simulans	ATPLGWMT-LSGSKSNGSDMHSITAKAVGISRDHAKVINYARIYGAGQLF	827
Drosophila virilis	ATPLGWMT-LSGSKSNGSDMHSITAKVVGISRDHAKVLNYARIYGAGQQF	880
Drosophila willistoni	ATPLGWMT-LSGNKSNQSDMHSITAKAVGISRDHAKVINYARIYGAGQQF	896
Drosophila mojavensis	ATPLGWMT-LSGSKSNGSDMHSITAKVVGISRDHAKVLNYARIYGAGQQF	875
Drosophila grimshawi	ATPLGWMT-LSGSKSNGSDMHSITAKVVGISRDHAKVLNYARIYGAGQQF	877
Culex quinquefasciatus	ATPLGWMT-LSGTKAAKTDMHSVTAQAVGISRDHAKVINYARIYGAGQNF	866

Anopheles gambiae	GTPFGWMT-LSGTKATRTDMHSVTAQAVGISRDHAKVLNYARIYGAGQQF	858
Tribolium castaneum	ATPFGWMT-LSGTKADSTDMHSVTAKAVGISRDHAKVINYARIYGAGQNF	831
Apis mellifera	ATPFGWMT-LIGTKANETDMHSVTAKAIGISRNQAKIINYARIYGAGQKF	831
Bombus impatiens	ATPFGWMT-LIGTKSNETDMHSVTAKAVGISRNQAKIINYARIYGAGQKY	980
Bombus terrestris	ATPFGWMT-LIGTKSNESDMHSVTAKAIGISRNQAKIINYARIYGAGQKF	960
Nasonia vitripennis	STPFSWMT-LIGNKSDGTDMHSVTAKAIGINRDQAKVLNYARIYGAGQKF	756
Danaus plexippus	--	
Strongylocentrotus purpuratus	CTAFSWMM-LQGKKNEGTDMHSMTASTVGISRDDAKVINYGRIYGAGEKF	927
Saccoglossus kowalevskii	CTAFGWMN-LQGNKLERTDLHSKTADTIDISRDQAKVFNYGRIYGAGQPF	1022
Branchiostoma floridae	CTSFGWMT-LQGKKSSGTDLHSKTASTIGISRDHAKVFNYGRIYGAGQPF	925
Ciona intestinalis	CTALSWMT-LRGNKKEGTDLHSTTAASIGISRDQAKVFNYGRMYGAGKKS	788
Oreochromis niloticus	CTAFGWMT-LQGKKSQGTDLHSRTADAVGISREHAKVFNYGRIYGAGQPF	932
Danio rerio	CTAFGWMT-LQGKKSQGTDLHSRTADAVGISREHAKVFNYGRIYGAGQPF	922
Xenopus tropicalis	CTAFGWMT-LQGKKSTGTDLHSKTASTVGISREHAKVFNYGRIYGAGQAF	928
Anolis carolinensis	CTAFGWMT-LQGKKSSGTDLHSKTASTVGISREHAKVFNYGRIYGAGQPF	891
Meleagris gallopavo	CTAFGWMTPRKGRKSDGTDLHSKTAATVGISREHAKVFNYGRIYGAGQPF	667
Gallus gallus	CTAFGWMT-LQGKKSDGTDLHSKTAATVGISREHAKVFNYGRIYGAGQPF	682
Monodelphis domestica	STAFGWMT-LQGKKSSGTDLHSKTASTVGISREHAKIFNYGRIYGAGQPF	1000
Mus musculus	CTAFGWMT-LQGRKSRGTDLHSKTAATVGISREHAKIFNYGRIYGAGQSF	939
Ailuropoda melanoleuca	CTAFGWMT-LQGRKSRGTDLHSKTAATVGISREHAKIFNYGRIYGAGQPF	954
Loxodonta africana	CTAFGWMT-LQGRKSRGTDLHSKTAATVGISREHAKIFNYGRIYGAGQPF	947
Callithrix jacchus	CTAFGWMT-LQGRKSRGTDLHSKTATTVGISREHAKVFNYGRIYGAGQPF	951
Pongo abelii	CTAFGWMT-LQGRKSRGTDLHSKTATTVGISREHAKIFNYGRIYGAGQPF	968
Homo sapiens	CTAFGWMT-LQGRKSRGTDLHSKTATTVGISREHAKIFNYGRIYGAGQPF	961

Saccharomyces cerevisiae	ASQLLKRFNPSLTDEETKKIANKLYENTKGKTKR----------------	797
Amphimedon queenslandica	ASTLLKQFNPLLSDEEIDAKSNSLYESTKGIRRM-LLSKKAQA------I	590
Trichoplax adhaerens	AQRLMRQFNPSMTLEDASDKAKILYASTKGITIY-KLTRKGKK------I	934
Nematostella vectensis	RKGCCCSLIIACTDTEARQKAERLYSSTKGT-AY-VLSDYGHE------V	980
Crassostrea gigas	AETLLMNFNHNLLPTEASAKARKLYETTKGKRKR----------------	919
Caenorhabditis elegans	AGKHLMRVG-GLKQSEAESTASQLFKLTKGDVAK-YMKVDVRMNCVVDKY	816
Oscheius tipulae	AEKALLK---SLKPEEAKEVAANLFSLTKGVVIE-CRQLSSLSVPLFREF	666
Loa loa	AAEFLMQ--SGMNSERALSVSNKLFATTKGKRFN-FLKLNENYNDYFRWY	796
Bursaphelenchus xylophilus	AIDFLVQ--KGISREVAKETAMKLFDTTKGTSGR-YRELQPHLIPYFEHF	792
Dirofilaria immitis	AAEFLMQ--SGMNAKKALSTSNKLFATTKGKRFN-FLKLNEKYNDYFRWY	589
Daphnia pulex	ARTLLMQFNHRLTEKEASEKARKMYSLTKGNSLL-KLSHYGRQ------L	959
Pediculus humanus corporis	AQKLLQQFNPLLNESDALKKATKIYQYTKGVKRF-TVRPEISK------F	893
Drosophila melanogaster	AETLLRQFNPTFSASEAKAKAMKMFSITKGKRVY-RLREEFHD-------	921
Drosophila erecta	AETLLRQFNPTFSASEAKAKAMKMFSITKGKRVY-RLREEFHD-------	918
Drosophila simulans	AETLLRQFNPTFSASEAKAKAIKMFSITKGKQV-----------------	860

Drosophila virilis	AETLLQQFNPTLSATEAKAKAMKMFAATKGKRVY-RLREEFHD-------	922
Drosophila willistoni	AENLLQQFNPTFSASEAKAKAMKMFSITKGKRIY-RLRDEFHD-------	938
Drosophila mojavensis	AETLLQQFNPTLSATEAKAKAMKMFAATKGKRVY-RLREEFHD-------	917
Drosophila grimshawi	AETLLQQFNPSLSATEAKAKAMKMFGATKGKRIY-RLREEFHD-------	919
Culex quinquefasciatus	AERLLKQFNPTFSDAEARSKAIKMFALTKGKKFY-YLKEEFRD-------	908
Anopheles gambiae	AERLLKQFNPTLTGAEARSKATKMFTLTKGRRTY-RLREELRD-------	900
Tribolium castaneum	AERLLKQFNPSMSDGEAKTKAAKMFALTKGKRIY-RLKKEFVL-------	873
Apis mellifera	AETLLKQFNPSMTDSEATLKSRKMFTMTKGKKIY-KLKPEYIN-------	873
Bombus impatiens	AETLLKQFNPCMTDSEATSKSRKMFTMTKGKKMY-RLKPEYIA-------	1022
Bombus terrestris	AETLLKQFNPCMTDSEAISKSRKMFTMTKGKKVY-RLKPEYID-------	1002
Nasonia vitripennis	AERLLKQFNPSITDKEAALKSKHIYLMTKGKKYY-RLKKQYVS-------	798
Danaus plexippus	--	
Strongylocentrotus purpuratus	AMRLLIQFNHRLTQDEARQKARDIYIATKGRRLY-RLSATGQS------L	970
Saccoglossus kowalevskii	AVRLLMQFNHRMSVDEARRKASTMYSATKGIKKY-KLKEDVRE------I	1065
Branchiostoma floridae	AERLLLQFNHRMTQEEAGEKAKQMYAATKGVRENPDYEEEGSYGAIIPRV	975
Ciona intestinalis	SLRSLMQFNKDLSREECIAKVNKLYGITKGEVQY-KLSPMTSG-------	830
Oreochromis niloticus	AERLLMQFNHRLSQTEAASKARQMYALTKGIRRY-HLSEDGEW------L	975
Danio rerio	AERLLMQFNHRLSQQEAAGKARQMYALTKGLRRY-HLSEEGEW------L	965
Xenopus tropicalis	AERLLMQFNHRLTQEQAAEKAKQMYAVTKGIRRY-ILSKEGEW------L	971
Anolis carolinensis	AERLLMQFNHRLTEQQASEKARQMYAATKGIRRC-HLSEEGEW------L	934
Meleagris gallopavo	AERLLMQFNHRLTQQQAREKAQQMYAVTKGIRRF-HLSEEGEW------L	710
Gallus gallus	AERLLMQFNHRLTQQQAREKAQQMYAVTKGIRRF-HLSEEGEW------L	725
Monodelphis domestica	AERLLMQFNHRLTPQQAKEKAQQMYAVTKGLRRY-QLSKDGEW------L	1043
Mus musculus	AERLLMQFNHRLTRQEAAEKAQQMYAVTKGLRRY-RLSADGEW------L	982
Ailuropoda melanoleuca	AERLLMQFNHRLTRQEAAEKAQQMYAVTKGLRRY-RLSDEGEW------L	997
Loxodonta africana	AERLLMQFNHRLTQEAAAEKAQQMYAVTKGLRRY-RLTDEGEW------L	990
Callithrix jacchus	AERLLMQFNHRLTRQEAAEKAQQMYAVTKGLRRY-RLSEEGEW------L	994
Pongo abelii	AERLLMQFNHRLTQQEAAEKAQQMYAATKGLRWY-RLSDEGEW------L	1011
Homo sapiens	AERLLMQFNHRLTQQEAAEKAQQMYAATKGLRWY-RLSDEGEW------L	1004
Saccharomyces cerevisiae	--	
Amphimedon queenslandica	ASSAG----------------ITIH--SDGS--INISD------WVKEYK	614
Trichoplax adhaerens	LQKLD-----------------PDTILDDDW--IDTTG------HEILRK	959
Nematostella vectensis	AHEFG----------------IPVD--EDGC--VETRV------YWELVR	1004
Crassostrea gigas	--	
Caenorhabditis elegans	IEEMVENSDVCKILTIDGIYYMPAYTSQFASETVRFEDWLLSHYSSLLNG	866
Oscheius tipulae	EKEHGEQNS----VVVDGHLFLPTYLMNYSKPVVEFENWVLKRLGVH---	709
Loa loa	IDNLCTSKMKTYYLYANGSYFLPEYRIRQGKLTLNFEDWLYEGVWNKLRE	846
Bursaphelenchus xylophilus	IQQICPKDFANTHLVLGKRYFLTIGNPTDRIQSTIFEDWLRHNMSAELEV	842
Dirofilaria immitis	VDNLCISNIKAYYVYANGSYFLPEYRVRQGELTLNFEDWLYENVWNKLRE	639

```
Daphnia pulex                   ASASG---------------------NKDEL--VTLEQ------LSEMRK 980
Pediculus humanus corporis      FQER-----------------------------FGCLEVNDMATAYQITK 914
Drosophila melanogaster         ------------------------E-LEDRA--YSSYE------ASRLAI 938
Drosophila erecta               ------------------------E-LDDRA--YSSYE------ASRLAI 935
Drosophila simulans             ---------------------------------YSSYE------ASRLAI 871
Drosophila virilis              ------------------------E-QEDRP--YSSYE------AKRLAL 939
Drosophila willistoni           ------------------------E-LEDRP--YSGYE------ATRLAV 955
Drosophila mojavensis           ------------------------E-QEDKA--YSSYE------ARRFAL 934
Drosophila grimshawi            ------------------------E-LEDRT--YSGYE------AKRLAL 936
Culex quinquefasciatus          ------------------------E-LPEKP--YSAYE------ALKTAK 925
Anopheles gambiae               ------------------------Q-YPTQPARSTAYE------ALGLAR 919
Tribolium castaneum             ------------------------E-YYDQN--YSHWQ------AFEIAK 890
Apis mellifera                  ------------------------N-NNDYDKLYSSYE------AYKLSK 892
Bombus impatiens                ------------------------E-HEDKD--YSLYE------AYRISK 1039
Bombus terrestris               ------------------------E-FENKD--YSSYE------AYKVSK 1019
Nasonia vitripennis             ------------------------ENLSNKL--YTSYE------AFKISQ 816
Danaus plexippus                --------------------------------------------------
Strongylocentrotus purpuratus   AEQLD----------------LDAEES-DGW--VTLEE------LKILSK 995
Saccoglossus kowalevskii        VRKL------------------LSKKKQSRW--ITNEE------LRTLRR 1089
Branchiostoma floridae          VTAGT----------------VTRRAVEPTW--LTASN------ARPDRV 1001
Ciona intestinalis              --------------------------------YASFDD------VTLIQN 842
Oreochromis niloticus           VNELG----------------IDVEREEDGS--VSLQE------LRRITR 1001
Danio rerio                     LQKLN----------------VNVERAEDGS--ISLQD------LRKISK 991
Xenopus tropicalis              VEELG----------------IPVERGEENS--VSLQD------LRKIQK 997
Anolis carolinensis             VKELG----------------IPVDRAENGS--VSLQD------VRKIHR 960
Meleagris gallopavo             VKELE----------------LAVDRAEDGT--VSAQD------VQKIQR 736
Gallus gallus                   VKELE----------------LAVDKAEDGT--VSAQD------VQKIQR 751
Monodelphis domestica           VKELG----------------IQVDRKEDGW--VSLQD------LRKIQK 1069
Mus musculus                    VKQLN----------------LPVDRTEDGW--VSLQD------LRMIQR 1008
Ailuropoda melanoleuca          VRELH----------------LPVDRTEDGW--VSLQD------LRKIQR 1023
Loxodonta africana              VRELD----------------LPVDRTEDGW--VSLQD------LRKIQR 1016
Callithrix jacchus              VRELH----------------LPVDRIEGGW--ISLQD------LRKVQR 1020
Pongo abelii                    VRELH----------------LPVDRTEGGW--ISLQD------LRKVQR 1037
Homo sapiens                    VRELN----------------LPVDRTEGGW--ISLQD------LRKVQR 1030

Saccharomyces cerevisiae        ------------------------------------SKLFKKFWYG---- 807
Amphimedon queenslandica        SFPPKS--------------------------------RVGTYWYG---- 628
Trichoplax adhaerens            HLRGKKLD--------------------------SYIKSKSKQWIG---- 979
Nematostella vectensis          RTTR---------------------------------------------- 1008
Crassostrea gigas               ---------------------------------------GEKVWEG---- 926
```

Caenorhabditis elegans	SS------EPDSLIYSIY----------------ENPDEPRRLFVG----	890
Oscheius tipulae	-----NEDEQKTVLALLY----------------EDITQKKELYFN----	734
Loa loa	KGQDEVDFSKDWLIKQIY-----------------DECTEYQLYTG----	875
Bursaphelenchus xylophilus	QG---IELDGRHFLNTVY----------------VNYPTSYTLFKD----	869
Dirofilaria immitis	NGKDEIDFSKNWLIKQIY-----------------GGCNEYQLYTG----	668
Daphnia pulex	NARKEPSLKQIANLP-------------------TDELVERRMWAG----	1007
Pediculus humanus corporis	ILNKK-----------------------------FGEIFSGSHWTR----	931
Drosophila melanogaster	QRNRT-----------------------------LAEVFHRPNWQG----	955
Drosophila erecta	QRNRT-----------------------------LTEVFHRPNWQG----	952
Drosophila simulans	QRNRT-----------------------------VAEVFHRPNWQG----	888
Drosophila virilis	QRNRP-----------------------------VGEVFHRPSWHG----	956
Drosophila willistoni	QRNRP-----------------------------ISEVFHRSSWQG----	972
Drosophila mojavensis	QRNRS-----------------------------VSEIFHRPSWQG----	951
Drosophila grimshawi	QRNRS-----------------------------IGEVFHRPSWQG----	953
Culex quinquefasciatus	ICNKP-----------------------------VTEIFERARWEG----	942
Anopheles gambiae	ACSRP-----------------------------VGELFEGPHWCG----	936
Tribolium castaneum	TWNRR-----------------------------IDDMFERPKWVG----	907
Apis mellifera	LHGKI-----------------------------ISEMFEKNKWVG----	909
Bombus impatiens	LYGKS-----------------------------LLEVFEKSRWVG----	1056
Bombus terrestris	LYGKS-----------------------------LSKVFEKGKWVG----	1036
Nasonia vitripennis	IHKKL-----------------------------RDEVFEKGKWIG----	833
Danaus plexippus	--	
Strongylocentrotus purpuratus	KAQIKS--	1001
Saccoglossus kowalevskii	RLKYTL--------EYQYVEDIESNIPLTDLLDYSFDLTEKREWKV----	1127
Branchiostoma floridae	GSELKAIVQAPPGYHFI-----------------GADVDSQELWIAAILG	1034
Ciona intestinalis	VANEASNYTARIN---------------------RNDLVCGRRWHG----	867
Oreochromis niloticus	VASQSS--------RRK-----------------RWDIVGKRLWAG----	1022
Danio rerio	VATQRG--------SKK-----------------KWNLTAGRVWTD----	1012
Xenopus tropicalis	EASAKS---------RR-----------------KWNLVSRRIWTG----	1017
Anolis carolinensis	EVGKRS--------QKK-----------------KWDVVRQRVWSN----	981
Meleagris gallopavo	EATRKS-------RRKK-----------------KWDVVAHRMWAG----	758
Gallus gallus	EAMRKS-------RRKK-----------------KWDVVAHRMWAG----	773
Monodelphis domestica	AASRMS--------YRK-----------------KWNLVADRAWAG----	1090
Mus musculus	EASRKS--------RWK-----------------KWEVASERAWTG----	1029
Ailuropoda melanoleuca	EASRKS--------RWK-----------------KWEVVAERAWMG----	1044
Loxodonta africana	EASRKS--------RRK-----------------KWEVVAERAWTG----	1037
Callithrix jacchus	EASRKS--------RWK-----------------KWEVVAERAWMG----	1041
Pongo abelii	ETARKS--------QWR-----------------KWEVIAERAWKG----	1058
Homo sapiens	ETARKS--------QWK-----------------KWEVVAERAWKG----	1051

Saccharomyces cerevisiae	--------GSESILFNKLESIAEQETPKTPVLGCGITYSLMK--------	841
Amphimedon queenslandica	--------GTESHMFNKLESIAKSPQPRTPVLNCLISTALQK--------	662
Trichoplax adhaerens	--------GSESEMFNALEAIALSEKPKTPILECRISQALEP--------	1013
Nematostella vectensis	--------RRESEMFNKLEMIAQSDEPRTPVLGCRISRALEP--------	1042
Crassostrea gigas	--------GTESEMFNMLESIARSASPKTPVLKASISKVLEP--------	960
Caenorhabditis elegans	--------GYESSTFNFLETSAAAHDLRTPILGCQIADSLGKLPEGTPDS	932
Oscheius tipulae	--------GYETATFNFLEQETKKDILRTPILNCQITDALAKLPPGTPDE	776
Loa loa	--------GFESDTFNYLELTLNNPKPCTPVLNCQLGYCLTPLPKDVRDH	917
Bursaphelenchus xylophilus	--------GFESATFNFLEIQAQNPEPRTPILECRLTQALEPLPQSVMGH	911
Dirofilaria immitis	--------GFESDTFNYLELTLND--------------------------	684
Daphnia pulex	--------GTESHMFNKLEEIAQSESPQTPVLHCRISRALEP--------	1041
Pediculus humanus corporis	--------GTESHMFNKLEKIASALEPKTPFLSCRLSTALEP--------	965
Drosophila melanogaster	--------GTESAMFNRLEEIATGSQPRTPFLGGRLSRALEA--------	989
Drosophila erecta	--------GTESAMFNRLEEIATGSQPRTPFLGGRLSRALEA--------	986
Drosophila simulans	--------GTESAMFNRLEEIATGSQPRTPFLGGRLSRALEA--------	922
Drosophila virilis	--------GTESAMFNRLEEIATREQPETPFLNCRLSRALES--------	990
Drosophila willistoni	--------GTESAMFNRLEEIATEAQPKTPFLGCRLSRALEA--------	1006
Drosophila mojavensis	--------GTESAMFNRLEEIATREQPETPFLNCRLSRALDS--------	985
Drosophila grimshawi	--------GTESAMFNRLEEIATREQPETPFLGCRLSRALET--------	987
Culex quinquefasciatus	--------GTESAMFNRLEEIADSPTPVTPFLGGRLSRALEP--------	976
Anopheles gambiae	--------GTESAMFNRLEQIAGSAEPETPFLGGRLSRAVEP--------	970
Tribolium castaneum	--------GTESAMFNRLESIATSQEPRTPFLNGRLSRALEP--------	941
Apis mellifera	--------GSESAMFNRLEEIACNPHPVTPFLNSRLTVALES--------	943
Bombus impatiens	--------GSESAMFNSLEEIARNPHPATPFLNSRLTTALEN--------	1090
Bombus terrestris	--------GSESAMFNSLEEIAQNPHPVTPFLNSRLTTALES--------	1070
Nasonia vitripennis	--------GSESVMFNSLEEIAEQNEPSTPFLNCRLSRALEY--------	867
Danaus plexippus	--	
Strongylocentrotus purpuratus	----------ESAMFNGLEDIAQSENPRTPVLQCSISRALQP--------	1033
Saccoglossus kowalevskii	--------GSESEMFNQLESIAMSDEPRTPVLGCRISRALEP--------	1161
Branchiostoma floridae	DANFAGFHGCTSFGWMTLQGKKSSGTD----LHSKTASTIGI--------	1072
Ciona intestinalis	--------GIESHMFNKVEEISNSKTPKTPALDCKISRALEP--------	901
Oreochromis niloticus	--------GTESDMFNKLESIAHSKNPATPVLGCRISRALEP--------	1056
Danio rerio	--------GTESYMFNKLESIAQSDRPATPVLNCRISRALEP--------	1046
Xenopus tropicalis	--------GTESQMFNKLETIAMSPSPKTPVLGCHISRALEP--------	1051
Anolis carolinensis	--------GTESEMFNKLESIAMSDSPCTPVLGCRISRALEP--------	1015
Meleagris gallopavo	--------GTESEMFNKLESIALSASPQTPVLGCHISRALEP--------	792
Gallus gallus	--------GTESEMFNKLESIALSASPQTPVLGCHISRALEP--------	807
Monodelphis domestica	--------GTESEMFNKLESIAMSDAPRTPVLGCQISRALEP--------	1124
Mus musculus	--------GTESEMFNKLESIAMSDTPRTPVLGCCISRALEP--------	1063
Ailuropoda melanoleuca	--------GTESEMFNKLESIAMSDTPCTPVLGCRISRALEP--------	1078

```
Loxodonta africana             --------GTESEMFNKLESIATSDTPRTPVLGCRISRALEP-------- 1071
Callithrix jacchus             --------GTESEMFNKLESIATSDIPCTPVLGCRISRALEP-------- 1075
Pongo abelii                   --------GTESEMFNKLESIATSDIPRTPVLGCRISRALEP-------- 1092
Homo sapiens                   --------GTESEMFNKLESIATSDIPRTPVLGCCISRALEP-------- 1085

Saccharomyces cerevisiae       KNL--RANSF-LPSRINWA------------------------------I 858
Amphimedon queenslandica       EN---VKEKF-MTSRINWV------------------------------V 678
Trichoplax adhaerens           KV---VGNAY-LPSRINWV------------------------------V 1029
Nematostella vectensis         WV---VGGAF-ITSRVNWV------------------------------V 1058
Crassostrea gigas              EI---IGDEF-MTSRINWV------------------------------V 976
Caenorhabditis elegans         AY---FDRKY-KRSVMNWI------------------------------V 948
Oscheius tipulae               EA---YDKKY-RRSVMNWVGLILGSHVRESRESDGQASKKKRDSRTERIV 822
Loa loa                        EY---FLKKY-RRSIINWV------------------------------V 933
Bursaphelenchus xylophilus     DD---FQMKY-KRTSINWV------------------------------V 927
Dirofilaria immitis            --------------------------------------------------
Daphnia pulex                  RN---VGNEF-MTSRVNWV------------------------------V 1057
Pediculus humanus corporis     HY---LNDDYHLNTRINWV------------------------------V 982
Drosophila melanogaster        DTGPEQEQRF-LPTRINWV------------------------------V 1008
Drosophila erecta              DGGPEQEQRF-LPTRINWV------------------------------V 1005
Drosophila simulans            DTGPDQEQRF-LPTRINWV------------------------------V 941
Drosophila virilis             HGESDQEQRF-LPTRVNWV------------------------------V 1009
Drosophila willistoni          DTGADQEQRF-LPTRINWV------------------------------V 1025
Drosophila mojavensis          NGEADLEQRF-LPTRINWV------------------------------V 1004
Drosophila grimshawi           RGDADQEQRF-LPTRVNWV------------------------------V 1006
Culex quinquefasciatus         QEG--TEDRF-LPTRINWV------------------------------V 993
Anopheles gambiae              QPG--TEDRF-LPTRINWV------------------------------V 987
Tribolium castaneum            KLS--SDDRF-LPTRINWV------------------------------V 958
Apis mellifera                 SV---NDEKF-LPTKINWV------------------------------V 959
Bombus impatiens               ST---NDEKY-LPTKVNWV------------------------------V 1106
Bombus terrestris              ST---NNEKY-LPTKINWV------------------------------V 1086
Nasonia vitripennis            E----KNEKF-LPTKINWV------------------------------V 882
Danaus plexippus               --------------------------------------------------
Strongylocentrotus purpuratus  DT---VNNEF-LTSRVNWV------------------------------V 1049
Saccoglossus kowalevskii       MS---VNNEF-LTSRINWV------------------------------V 1177
Branchiostoma floridae         SR---DHAKF-VTSRVNWV------------------------------V 1088
Ciona intestinalis             GK---VNGNF-VTTRMNWV------------------------------V 917
Oreochromis niloticus          KA---VKDEF-ITSRVNWV------------------------------V 1072
Danio rerio                    SA---VQEEF-ITSRVNWV------------------------------V 1062
Xenopus tropicalis             AA---VKGEF-ITSRINWV------------------------------V 1067
Anolis carolinensis            AA---VKGEF-VTSRVNWV------------------------------V 1031
```

```
Meleagris gallopavo              SV---AKGEF-LTSRVNWV------------------------------V 808
Gallus gallus                    AV---AKGEF-LTSRVNWV------------------------------V 823
Monodelphis domestica            AV---VQGEF-VTSRVNWV------------------------------V 1140
Mus musculus                     SV---VQGEF-ITSRVNWV------------------------------V 1079
Ailuropoda melanoleuca           SA---VQGEF-MTSRVNWV------------------------------V 1094
Loxodonta africana               SA---VQGEF-MTSRVNWV------------------------------V 1087
Callithrix jacchus               SV---VQGEF-MTSRVNWV------------------------------V 1091
Pongo abelii                     SA---VREEF-MTSRVNWV------------------------------V 1108
Homo sapiens                     SA---VQEEF-MTSRVNWV------------------------------V 1101

Saccharomyces cerevisiae         QSSGVDYLHLLCCSMEYIIK---------KYN-----LEARLCISIHDEI 894
Amphimedon queenslandica         QSSAVDYLHLLLVAVKWLMA---------HYN----ITGGRLCISIHDEV 715
Trichoplax adhaerens             QSSAVDYLHLILVCMRWLFE---------KYN-----INGRFCISIHDEV 1065
Nematostella vectensis           QSSAVDYLHLMLVSMKWLFT---------EYG-----IDGRFCISIHDEV 1094
Crassostrea gigas                QSSAVDYLHLMLVCMNWLCN---------QYD-----IDARFSISIHDEV 1012
Caenorhabditis elegans           QSSAVDFLHLLLVSMQWLCD---------TYK-----IDARFVISIHDEV 984
Oscheius tipulae                 QSSAVDFLHLLLVAMNWLCH---------EYE-----IDARFVISIHDEV 858
Loa loa                          QSSAVDFLHLLIVCMKWLCE---------IYQ-----IEARFALSIHDEI 969
Bursaphelenchus xylophilus       QSSAVDFLHLLLVCMKWLCQ---------EYD-----IRARFVISIHDE- 962
Dirofilaria immitis              --------------------------------------------------
Daphnia pulex                    QSSAVDYLHLMIVAMRYLVD---------VYKLGVVLIEGRFSISIHDEV 1098
Pediculus humanus corporis       QSGAVDFLHLMLVCMRWLTN---------GHA--------RYCFSFHDEI 1015
Drosophila melanogaster          QSGAVDFLHLMLVSMRWLMG---------SHV--------RFCLSFHDEL 1041
Drosophila erecta                QSGAVDFLHLMLVSMRWLMG---------SHV--------RFCLSFHDEL 1038
Drosophila simulans              QSGAVDFLHLMLVSMRWLMG---------SHV--------RFCLSFHDEL 974
Drosophila virilis               QSGAVDFLHLMLVSMRWLLG---------THA--------RFCLSFHDEL 1042
Drosophila willistoni            QSGAVDFLHLMLVSMRWLMG---------SHA--------RFCLSFHDEL 1058
Drosophila mojavensis            QSGAVDFLHLMLVSMRWLLG---------PHA--------RFCLSFHDEL 1037
Drosophila grimshawi             QSGAVDFLHLMLVSMRWLLG---------PHA--------RFCLSFHDEL 1039
Culex quinquefasciatus           QSGAVDFLHLMLVAMRWLMG---------DRV--------RFCLSFHDEV 1026
Anopheles gambiae                QSGAVDFLHLMLVCMRWLMGGSDVAANSSRRL--------RFCLSFHDEV 1029
Tribolium castaneum              QSGAADFLHLMLVCMRWIMG---------ENA--------RFCLSFHDEV 991
Apis mellifera                   QSGAVDFLHLMLVSMKWLMK---------DHA--------RFCLSFHDEV 992
Bombus impatiens                 QSGAVDFLHLMLVSMKWLMK---------DNI--------RFCLSFHDEV 1139
Bombus terrestris                QSGAVDFLHLMLVSMKWLMK---------DNI--------RFCLSFHDEV 1119
Nasonia vitripennis              QSGAVDFLHLILVTMRWLMK---------DKA--------RLCLSFHDEV 915
Danaus plexippus                 --------------------------------------------------
Strongylocentrotus purpuratus    QSSAVDYLHLMLVCMRWLFE---------MYN-----IDGRFCISIHDEV 1085
Saccoglossus kowalevskii         QSSAVDYLHLMLVCMRWLLD---------KYD-----IDGRFSISIHDEV 1213
Branchiostoma floridae           QSSAVDYLHLMLVCMRWLFN---------KYN-----IDGRFCISIHDEV 1124
```

Ciona intestinalis	QSSAVDYLHLLLVSMRWLIE---------KYK-----LDCRFVLSIHDEV	953
Oreochromis niloticus	QSSAVDYLHLMLVVMKWLIE---------EYN-----IDGRFCISIHDEV	1108
Danio rerio	QSSAVDYLHLMLVSMRWLFE---------EHD-----IDGRFCISIHDEV	1098
Xenopus tropicalis	QSSAVDYLHLMLVAMKWLFE---------AYD-----IDGRFCISIHDEV	1103
Anolis carolinensis	QSSAVDYLHLMLVAMKWLFE---------EYS-----IDGRFCISIHDEV	1067
Meleagris gallopavo	QSSAVDYLHLMLVSMKWLFE---------EYD-----INGRFCISIHDEV	844
Gallus gallus	QSSAVDYLHLMLVSMKWLFE---------EYD-----INGRFCISIHDEV	859
Monodelphis domestica	QSSAVDYLHLMLVAMKWLFE---------EFD-----ISGRFCISIHDEV	1176
Mus musculus	QSSAVDYLHLMLVAMKWLFE---------EFA-----IDGRFCISIHDEV	1115
Ailuropoda melanoleuca	QSSAVDYLHLMLVAMKWLFE---------EFA-----IDGRFCISIHDEV	1130
Loxodonta africana	QSSAVDYLHLMLVAMKWLFE---------EFG-----IDGRFCISIHDEV	1123
Callithrix jacchus	QSSAVDYLHLMLVAMKWLFE---------EFA-----IDGRFCISIHDEV	1127
Pongo abelii	QSSAVDYLHLMLVAMKWLFE---------EFA-----IDGRFCISIHDEV	1144
Homo sapiens	QSSAVDYLHLMLVAMKWLFE---------EFA-----IDGRFCISIHDEV	1137

Saccharomyces cerevisiae	RFLVSEKDKYRAAMALQISNIWTRAMFCQQMGINELPQNCAFFSQVDIDS	944
Amphimedon queenslandica	RYIVREEDKYKMSLALQVANIWTRAMFAPSLGMNDLPL------------	753
Trichoplax adhaerens	RYLVKSEDRFRAALALQITNLLTRAMFAHKIKIYDLPLSVAFFSAVDVDT	1115
Nematostella vectensis	RYLVAEEDKYRAALALQITNLLTRAMFAYKLGMNDLPQSVAFFSAVDIDK	1144
Crassostrea gigas	RYLVSSKDRYRAVLALQITNLLTRSMFARALGMNDLPLSVAFFSKVDIDQ	1062
Caenorhabditis elegans	RYMCKEPDAPRLALALQLSNLLVRAYISQRVGICQLPNTVAFFSQVDCDT	1034
Oscheius tipulae	RYLCPDEEAPRVALALMLAHLYVRTFISHRVGFRQLPSSIAFFSQVDCDT	908
Loa loa	RYIVPAEDRYRCALALSLSNMYVRAMISQKLGIRELPMSVAFFSQVDIDR	1019
Bursaphelenchus xylophilus	--------------------------FTKKINIAVL--------------	972
Dirofilaria immitis	--	
Daphnia pulex	RYIIKEEDKFRAALALQVANLWTRCMFSYQMNLNDLPQAVAFFSSVDIDT	1148
Pediculus humanus corporis	RYLFPSEQKYSGALAVHLTNLFVRSFCCHRLGIYDLPQSVAFFSSVEVDT	1065
Drosophila melanogaster	RYLVKEELSPKAALAMHITNLMTRSFCVSRIGLQDLPMSVAFFSSVEVDT	1091
Drosophila erecta	RYLVKEEFSPKAALAMHITNLMTRSFCVSRIGLKDLPMSVAFFSSVEVDT	1088
Drosophila simulans	RYLVKEELSPKAALAMHITNLMTRSFCVSRIGLQDLPMSVAFFSSVEVDT	1024
Drosophila virilis	RYLVKDELAAKAALAMHVTNLLTRSFCASRIGLTDLPMSVAFFSSVEVDT	1092
Drosophila willistoni	RYLVREDMAPKAALAMHITNLLTRSFCVSRIGLKDLPMSVAFFSSVEVDT	1108
Drosophila mojavensis	RYLVKDEMAPKAALAMHVTNLLTRSFCASRIGLKDLPMSVAFFSSVEVDT	1087
Drosophila grimshawi	RYLVKDELAYKAALAMHVTNLLTRSFCASKIGLTDLPMSVAFFSSVEVDT	1089
Culex quinquefasciatus	RYLVEDQYAYRAALAMHVTNLLTRAFCVSRIGLADLPQSVAFFSTVEVDS	1076
Anopheles gambiae	RYLVPERYAHHAALALHLTNLLTRAFCVHRVGFRDLPQSVAFFSSVEVDR	1079
Tribolium castaneum	RYLVPERFKYQGALAMHVTNLLTRAFCSVRLGIYDLPQSVAFFSSVEVDT	1041
Apis mellifera	RYLVPSKYKYNAALAMHITNLLTRSFCASRLGMNDLPMSVAFFTSVEVDT	1042
Bombus impatiens	RYLVPSRYKYNAALAMHITNLLTRSFCASRLGMNDLPMSVAFFSSVEVDT	1189
Bombus terrestris	RYLVPSRYKYNAALAMHITNLLTRSFCASRLGMNDLPMSVAFFSSVEVDT	1169

Nasonia vitripennis	RYLVPSEYKYNAALAMHVTNLLVRCFFVSRLKMKDLPMSVAFFTSVEVDS	965
Danaus plexippus	--	
Strongylocentrotus purpuratus	RYLVKSEDRYRAALALQITNLLTRAVFAYNMGMNDLPQGMK---------	1126
Saccoglossus kowalevskii	RYLVNSNDKYRAAMALQISNLLTRCMFAYKLGMLDLPQSVAFFSAVDIDT	1263
Branchiostoma floridae	RYLVQSEDRYRAALALQITNLLTRSMFAYRLGMNDLPQSVAFFSAVDVDT	1174
Ciona intestinalis	RYMSSYEHRHKTALALQISNLLTRSMFAYKLGMDDLPLGVAFFSCIEVDS	1003
Oreochromis niloticus	RYLVRSEDRYRAALALQITNLLTRSMFAHALGMQDLPQSVAFFSAVDIDQ	1158
Danio rerio	RYLVTSEDRYRAALALQITNLLTRCMFAFKLGMMDLPQSVAFFSAVDIDK	1148
Xenopus tropicalis	RYLVQSKDRYRAALALQITNLLTRCMFASRLGMQDLPQSVAFFSAVDIDK	1153
Anolis carolinensis	RYLVQSEDRYRAALALQITNLLTRCMFAYKLGLQDLPQSVAFFSAVDIDQ	1117
Meleagris gallopavo	RYLVQEQDRYRAALALQITNLLTRCMFAYKLGLQDLPQSVAFFSAVDIDR	894
Gallus gallus	RYLVQEQDRYRAALALQITNLLTRCMFAYKLGLQDLPQSVAFFSAVDIDR	909
Monodelphis domestica	RYLVTEKDRYRAALALQITNLLTRCMFAYKLGLNDLPQSVAFFSAVDIDR	1226
Mus musculus	RYLVREEDRYRAALALQITNLLTRCMFAYKLGLNDLPQSVAFFSAVDIDQ	1165
Ailuropoda melanoleuca	RYLVREEDRYRAALALQITNLLTRCMFAYKLGLNDLPQSVAFFSTVDIDQ	1180
Loxodonta africana	RYLVREEDRYRAALALQVTNLLTRCMFAYKLGLNDLPQSVAFFSAVDIDQ	1173
Callithrix jacchus	RYLVREEDRYRAALALQITNLLTRCMFAYKLGLNDLPQSVAFFSAVDIDR	1177
Pongo abelii	RYLVREEDRYRAALALQITNLLTRCMFAYKLGLNDLPQSVAFFSAVDIDR	1194
Homo sapiens	RYLVREEDRYRAALALQITNLLTRCMFAYKLGLNDLPQSVAFFSAVDIDR	1187

Saccharomyces cerevisiae	VIRKEVNMDCITPSN------KTAIPHGEALDINQLLDKS-NS---KLGK	984
Amphimedon queenslandica	--	
Trichoplax adhaerens	VLRKEPDMDCKTPTNPLGLEKGYNVPPGIVH-------------------	1146
Nematostella vectensis	VLRKEVNLDCKTPSNPQGLQKGQGIMP-EALDIHQILQKT-NG---CLRK	1189
Crassostrea gigas	CLRKEVGLECKTPSNPSGMERAYNIPFGEGQDIYQTLQIT-NG---SLSA	1108
Caenorhabditis elegans	VLRKEVDTESINPD-------GSKIADGVAWTVDDLLKLT-GG---KLD-	1072
Oscheius tipulae	VLRKEVTTKCINPD-------GEPIPAGVNWNIEEIVAIT-GG---SLKK	947
Loa loa	VLRKEVNLVCTTPS-------GECIQPGEALDMSAILKKT-GG---TLKK	1058
Bursaphelenchus xylophilus	--	
Dirofilaria immitis	--	
Daphnia pulex	VLRKESNMDCQTPSNPYGLKQGYGIDFGCSYDIYQILEKA-GG---SLDS	1194
Pediculus humanus corporis	ILRKEAKDDCITPSNPHGMEKGYGIPPGESLNIYETIKKS-GG---SLSV	1111
Drosophila melanogaster	VLRKECTMDCKTPSNPHGLRIGYGIQPGQSLSVAEAIEKA-GG--NDVSQ	1138
Drosophila erecta	VLRKECTMDCKTPSNPHGLRIGYGIQPGQSLSVAEAIEKA-GG--NDVSQ	1135
Drosophila simulans	VLRKECTMDCKTPSNPHGLRIGYGIQPGQSLSVAEAIEEA-GG--NDVSQ	1071
Drosophila virilis	VLRKECTLDCQTPSNPHGLQIGYGISPGQSLSIEDAIAKA-GG--HDLSQ	1139
Drosophila willistoni	VLRKECSMDCQTPSNPHGLRIGYGIQPGESLTIDKAIEKA-GG--HDLSQ	1155
Drosophila mojavensis	VLRKECTMDCQTPSNPHGLQIGYGIPPGQSLGIQEAIVKA-GG--GDLTK	1134
Drosophila grimshawi	VLRKECTMDCQTPSNPHGLQIGYGIAPGQSLSIEQSIQKA-EG--HDLNQ	1136
Culex quinquefasciatus	VLRKESHLDCRTPSNPHGLAIGYGIPNGESLDIAQLLEKL-GPHESDMQT	1125

Anopheles gambiae	VLRKEAQHDCRTPSNPHGLRVGYCIPDGESLDIYQTLARL-TATERDVSR	1128
Tribolium castaneum	VLRKDAKDDCMTPSNPHGLEKGYNIPNGEGLDINQAVKKA-GG---KFSY	1087
Apis mellifera	VLRKEPENDCITPSNPHGLKNEYEIPPGESLDIWSAIDKS-KG---SLGP	1088
Bombus impatiens	VLRKEPENDCITPSNPYGLKNGYEVPSGESLDVWSAIDKS-KG---SLGS	1235
Bombus terrestris	VLRKEPDNDCITPSNPYGLKNGYEIPSGESLDVWSAIDKS-KG---SLGS	1215
Nasonia vitripennis	VLRKETSQDCKTPSNPHGLKKGYGIQPGENLDIWTAVKKS-KG---YVGM	1011
Danaus plexippus	--	
Strongylocentrotus purpuratus	-----------------------TINTGEALDIYEILNLTKNG---SLTR	1150
Saccoglossus kowalevskii	VLRKEVSMDCKTPSNPHGLEKGYGIPEGEALDIYDVLKLTNNG---SLEK	1310
Branchiostoma floridae	VMRKEVNMDCKTPSNPHGLTRGYGIPPGDALDIYQILEKT-NS---SLRK	1220
Ciona intestinalis	VVRKSADDDCVTPSNSEGLSARYGIPLGESYDVNEIL-------------	1040
Oreochromis niloticus	CLRKEVNMDCVTPSNPTGVERKYGLPPGEALDIYQIIDIT-KG---SLNK	1204
Danio rerio	CLRKEVTMDCKTPSSPAGVERRYGLPQGEALDIYQIIEIT-KG---SLTK	1194
Xenopus tropicalis	CLRKEVTMDCATPSNPTGMEKRYGIPQGEALDIYQILKVT-KG---VL--	1197
Anolis carolinensis	CLRKEVTMDCVTPSNPTGMERRYSIPQGEALDIYQLLKIT-KS---SLEK	1163
Meleagris gallopavo	CLRKEVTMNCVTPSNPTGMEKKYGIPRGEALDIYQIIEIT-KG---SLEK	940
Gallus gallus	CLRKEVTMNCATPSNPTGMEKKYGIPRGEALDIYQIIEIT-KG---SLEK	955
Monodelphis domestica	CLRKEVTMDCKTPSNPTGLERRYGIPQGEALDIYQIIKLT-KG---SLEK	1272
Mus musculus	CLRKEVTMDCKTPSNPTGMERRYGIPQGEALDIYQIIELT-KG---SLEK	1211
Ailuropoda melanoleuca	CLRKEVTMDCKTPSNPTGMERRYGIPQGEALDIYQIIELT-KG---SLEP	1226
Loxodonta africana	CLRKEVTMDCKTPSNPTGMERRYRIPQGEALDIYQIIELT-KG---SLEK	1219
Callithrix jacchus	CLRKEVTMDCKTPSNPTGMERRYGIPQGEALDIYQIIELT-KG---SLEK	1223
Pongo abelii	CLRKEVTMDCKTPSNPTGMERRYGIPQGEALDIYQIIELT-KG---SLEK	1240
Homo sapiens	CLRKEVTMDCKTPSNPTGMERRYGIPQGEALDIYQIIELT-KG---SLEK	1233

Saccharomyces cerevisiae	PNLDIDSKVSQYAYNYREPVFEEYNKSYTPEFLKYFLAMQVQSDKRDVNR	1034
Amphimedon queenslandica	--	
Trichoplax adhaerens	--	
Nematostella vectensis	--	
Crassostrea gigas	AE-------E--	1111
Caenorhabditis elegans	--	
Oscheius tipulae	NSQQRQLDL---	956
Loa loa	VAVAEV--	1064
Bursaphelenchus xylophilus	--	
Dirofilaria immitis	--	
Daphnia pulex	KT--	1196
Pediculus humanus corporis	FF-------K--	1114
Drosophila melanogaster	WD-------W--	1141
Drosophila erecta	WN-------W--	1138
Drosophila simulans	WD-------W--	1074

```
Drosophila virilis              WP-------W---------------------------------------- 1142
Drosophila willistoni           WN-------W---------------------------------------- 1158
Drosophila mojavensis           WP-------W---------------------------------------- 1137
Drosophila grimshawi            WP-------W---------------------------------------- 1139
Culex quinquefasciatus          WE-------W---------------------------------------- 1128
Anopheles gambiae               WQ-------W---------------------------------------- 1131
Tribolium castaneum             WY-------EQ--------------------------------------- 1091
Apis mellifera                  WT------------------------------------------------ 1090
Bombus impatiens                WH------------------------------------------------ 1237
Bombus terrestris               WH------------------------------------------------ 1217
Nasonia vitripennis             F------------------------------------------------- 1012
Danaus plexippus                --------------------------------------------------
Strongylocentrotus purpuratus   TA-------S---------------------------------------- 1153
Saccoglossus kowalevskii        PK-------IR--------------------------------------- 1314
Branchiostoma floridae          ED-------Q---------------------------------------- 1223
Ciona intestinalis              --------------------------------------------------
Oreochromis niloticus           AR------------------------------------------------ 1206
Danio rerio                     EK-------R---------------------------------------- 1197
Xenopus tropicalis              --------------------------------------------------
Anolis carolinensis             EK------------------------------------------------ 1165
Meleagris gallopavo             K------------------------------------------------- 941
Gallus gallus                   K------------------------------------------------- 956
Monodelphis domestica           RS------------------------------------------------ 1274
Mus musculus                    RS-------Q---------------------------------------- 1214
Ailuropoda melanoleuca          --------------------------------------------------
Loxodonta africana              RS-------Q---------------------------------------- 1222
Callithrix jacchus              RS-------Q---------------------------------------- 1226
Pongo abelii                    RS-------Q---------------------------------------- 1243
Homo sapiens                    RS-------Q---------------------------------------- 1236

Saccharomyces cerevisiae        LEDEYLRECTSKEYARDGNTAEYSLLDYIKDVEKGKRTKVRIMGSNFLDG 1084
Amphimedon queenslandica        --------------------------------------------------
Trichoplax adhaerens            --------------------------------------------------
Nematostella vectensis          --------------------------------------------------
Crassostrea gigas               --------------------------------------------------
Caenorhabditis elegans          --------------------------------------------------
Oscheius tipulae                --------------------------------------------------
Loa loa                         --------------------------------------------------
Bursaphelenchus xylophilus      --------------------------------------------------
Dirofilaria immitis             --------------------------------------------------
```

```
Daphnia pulex                    --------------------------------------------------
Pediculus humanus corporis       --------------------------------------------------
Drosophila melanogaster          --------------------------------------------------
Drosophila erecta                --------------------------------------------------
Drosophila simulans              --------------------------------------------------
Drosophila virilis               --------------------------------------------------
Drosophila willistoni            --------------------------------------------------
Drosophila mojavensis            --------------------------------------------------
Drosophila grimshawi             --------------------------------------------------
Culex quinquefasciatus           --------------------------------------------------
Anopheles gambiae                --------------------------------------------------
Tribolium castaneum              --------------------------------------------------
Apis mellifera                   --------------------------------------------------
Bombus impatiens                 --------------------------------------------------
Bombus terrestris                --------------------------------------------------
Nasonia vitripennis              --------------------------------------------------
Danaus plexippus                 --------------------------------------------------
Strongylocentrotus purpuratus    --------------------------------------------------
Saccoglossus kowalevskii         --------------------------------------------------
Branchiostoma floridae           --------------------------------------------------
Ciona intestinalis               --------------------------------------------------
Oreochromis niloticus            --------------------------------------------------
Danio rerio                      --------------------------------------------------
Xenopus tropicalis               --------------------------------------------------
Anolis carolinensis              --------------------------------------------------
Meleagris gallopavo              --------------------------------------------------
Gallus gallus                    --------------------------------------------------
Monodelphis domestica            --------------------------------------------------
Mus musculus                     --------------------------------------------------
Ailuropoda melanoleuca           --------------------------------------------------
Loxodonta africana               --------------------------------------------------
Callithrix jacchus               --------------------------------------------------
Pongo abelii                     --------------------------------------------------
Homo sapiens                     --------------------------------------------------

Saccharomyces cerevisiae         TKNAKADQRIRLPVNMPDYPTLHKIANDSAIPEKQLLENRRKKENRIDDE 1134
Amphimedon queenslandica         --------------------------------------------------
Trichoplax adhaerens             --------------------------------------------------
Nematostella vectensis           --------------------------------------------------
Crassostrea gigas                --------------------------------------------------
```

```
Caenorhabditis elegans          --------------------------------------------------
Oscheius tipulae                --------------------------------------------------
Loa loa                         --------------------------------------------------
Bursaphelenchus xylophilus      --------------------------------------------------
Dirofilaria immitis             --------------------------------------------------
Daphnia pulex                   --------------------------------------------------
Pediculus humanus corporis      --------------------------------------------------
Drosophila melanogaster         --------------------------------------------------
Drosophila erecta               --------------------------------------------------
Drosophila simulans             --------------------------------------------------
Drosophila virilis              --------------------------------------------------
Drosophila willistoni           --------------------------------------------------
Drosophila mojavensis           --------------------------------------------------
Drosophila grimshawi            --------------------------------------------------
Culex quinquefasciatus          --------------------------------------------------
Anopheles gambiae               --------------------------------------------------
Tribolium castaneum             --------------------------------------------------
Apis mellifera                  --------------------------------------------------
Bombus impatiens                --------------------------------------------------
Bombus terrestris               --------------------------------------------------
Nasonia vitripennis             --------------------------------------------------
Danaus plexippus                --------------------------------------------------
Strongylocentrotus purpuratus   --------------------------------------------------
Saccoglossus kowalevskii        --------------------------------------------------
Branchiostoma floridae          --------------------------------------------------
Ciona intestinalis              --------------------------------------------------
Oreochromis niloticus           --------------------------------------------------
Danio rerio                     --------------------------------------------------
Xenopus tropicalis              --------------------------------------------------
Anolis carolinensis             --------------------------------------------------
Meleagris gallopavo             --------------------------------------------------
Gallus gallus                   --------------------------------------------------
Monodelphis domestica           --------------------------------------------------
Mus musculus                    --------------------------------------------------
Ailuropoda melanoleuca          --------------------------------------------------
Loxodonta africana              --------------------------------------------------
Callithrix jacchus              --------------------------------------------------
Pongo abelii                    --------------------------------------------------
Homo sapiens                    --------------------------------------------------
```

Saccharomyces cerevisiae	NKKKLTRKKNTTPMERKYKRVYGGRKAFEAFYECANKPLDYTLETEKQFF	1184
Amphimedon queenslandica	--	
Trichoplax adhaerens	--	
Nematostella vectensis	--	
Crassostrea gigas	--	
Caenorhabditis elegans	--	
Oscheius tipulae	--	
Loa loa	--	
Bursaphelenchus xylophilus	--	
Dirofilaria immitis	--	
Daphnia pulex	--	
Pediculus humanus corporis	--	
Drosophila melanogaster	--	
Drosophila erecta	--	
Drosophila simulans	--	
Drosophila virilis	--	
Drosophila willistoni	--	
Drosophila mojavensis	--	
Drosophila grimshawi	--	
Culex quinquefasciatus	--	
Anopheles gambiae	--	
Tribolium castaneum	--	
Apis mellifera	--	
Bombus impatiens	--	
Bombus terrestris	--	
Nasonia vitripennis	--	
Danaus plexippus	--	
Strongylocentrotus purpuratus	--	
Saccoglossus kowalevskii	--	
Branchiostoma floridae	--	
Ciona intestinalis	--	
Oreochromis niloticus	--	
Danio rerio	--	
Xenopus tropicalis	--	
Anolis carolinensis	--	
Meleagris gallopavo	--	
Gallus gallus	--	
Monodelphis domestica	--	
Mus musculus	--	
Ailuropoda melanoleuca	--	

```
Loxodonta africana             --------------------------------------------------
Callithrix jacchus             --------------------------------------------------
Pongo abelii                   --------------------------------------------------
Homo sapiens                   --------------------------------------------------

Saccharomyces cerevisiae       NIPIDGVIDDVLNDKSNYKKKPSQARTASSSPIRKTAKAVHSKKLPARKS 1234
Amphimedon queenslandica       --------------------------------------------------
Trichoplax adhaerens           --------------------------------------------------
Nematostella vectensis         --------------------------------------------------
Crassostrea gigas              ----------------------------------------LQERKTEFA- 1120
Caenorhabditis elegans         --------------------------------------------------
Oscheius tipulae               --------------------------------------------------
Loa loa                        --------------------------------------------------
Bursaphelenchus xylophilus     --------------------------------------------------
Dirofilaria immitis            --------------------------------------------------
Daphnia pulex                  ---------------------------------------KNCSKESPVN- 1206
Pediculus humanus corporis     ----------------------------------------KSGRVEPFKC 1124
Drosophila melanogaster        ----------------------------------------IKKS------ 1145
Drosophila erecta              ----------------------------------------IKKS------ 1142
Drosophila simulans            ----------------------------------------IKKS------ 1078
Drosophila virilis             ----------------------------------------LKRPKELS-- 1150
Drosophila willistoni          ----------------------------------------ISKS------ 1162
Drosophila mojavensis          ----------------------------------------LKPSQN---- 1143
Drosophila grimshawi           ----------------------------------------LKRHEQLS-- 1147
Culex quinquefasciatus         ----------------------------------------HQPATQPAKR 1138
Anopheles gambiae              ----------------------------------------HRAKPPKPNR 1141
Tribolium castaneum            -------------------------------------LK----------- 1093
Apis mellifera                 --------------------------------------------------
Bombus impatiens               ---------------------------------------------NVKQR 1242
Bombus terrestris              ---------------------------------------------NVKQ- 1221
Nasonia vitripennis            --------------------------------------------------
Danaus plexippus               --------------------------------------------------
Strongylocentrotus purpuratus  ----------------------------------------PSNKNQESRS 1163
Saccoglossus kowalevskii       -------------------------------------K------------ 1315
Branchiostoma floridae         ----------------------------------------HKEKKPNKR- 1232
Ciona intestinalis             --------------------------------------------------
Oreochromis niloticus          --------------------------------------------------
Danio rerio                    ----------------------------------------QAPRSK---- 1203
Xenopus tropicalis             --------------------------------------------------
Anolis carolinensis            --------------------------------------------------
```

Meleagris gallopavo --
Gallus gallus --
Monodelphis domestica --
Mus musculus --PGP------- 1217
Ailuropoda melanoleuca --
Loxodonta africana --PGP------- 1225
Callithrix jacchus --PGP------- 1229
Pongo abelii --PGP------- 1246
Homo sapiens --PGP------- 1239

Saccharomyces cerevisiae STTNRNLVELERDITISRE 1253
Amphimedon queenslandica -------------------
Trichoplax adhaerens -------------------
Nematostella vectensis -------------------
Crassostrea gigas -------------------
Caenorhabditis elegans -------------------
Oscheius tipulae -------------------
Loa loa -------------------
Bursaphelenchus xylophilus -------------------
Dirofilaria immitis -------------------
Daphnia pulex -------------------
Pediculus humanus corporis -------------------
Drosophila melanogaster -------------------
Drosophila erecta -------------------
Drosophila simulans -------------------
Drosophila virilis -------------------
Drosophila willistoni -------------------
Drosophila mojavensis -------------------
Drosophila grimshawi -------------------
Culex quinquefasciatus PKKHLK--------LIKSA 1149
Anopheles gambiae KSKQQKDDLHQAGRAGERT 1160
Tribolium castaneum -------------------
Apis mellifera -----------------AK 1092
Bombus impatiens IITPNKKYKLKEIKPKTDE 1261
Bombus terrestris -------------------
Nasonia vitripennis -------------------
Danaus plexippus -------------------
Strongylocentrotus purpuratus NGRPQAVEAVG-------- 1174
Saccoglossus kowalevskii -------------------
Branchiostoma floridae ------VSVGL-------- 1237

```
Ciona intestinalis             -------------------
Oreochromis niloticus          -------------------
Danio rerio                    -------------------
Xenopus tropicalis             -------------------
Anolis carolinensis            -------------------
Meleagris gallopavo            -------------------
Gallus gallus                  -------------------
Monodelphis domestica          -------------------
Mus musculus                   -------------------
Ailuropoda melanoleuca         -------------------
Loxodonta africana             -------------------
Callithrix jacchus             -------------------
Pongo abelii                   -------------------
Homo sapiens                   -------------------
```

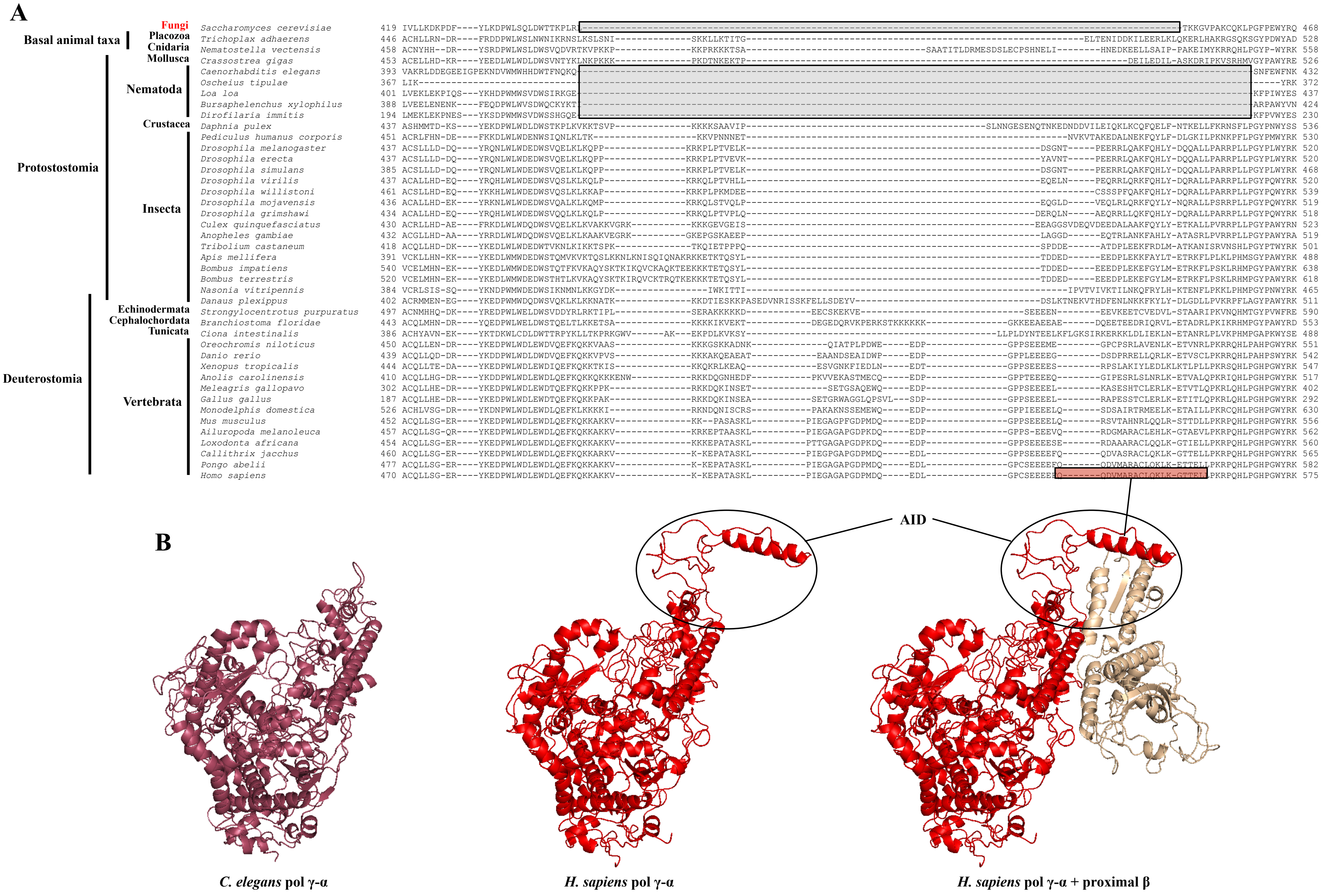

A
Fungi
Basal animal taxa
Placozoa
Cnidaria
Mollusca
Nematoda
Crustacea
Protostostomia
Insecta
Echinodermata
Cephalochordata
Tunicata
Deuterostomia
Vertebrata
Saccharomyces cerevisiae
Trichoplax adhaerens
Nematostella vectensis
Crassostrea gigas
Caenorhabditis elegans
Oscheius tipulae
Loa loa
Bursaphelenchus xylophilus
Dirofilaria immitis
Daphnia pulex
Pediculus humanus corporis
Drosophila melanogaster
Drosophila erecta
Drosophila simulans
Drosophila virilis
Drosophila willistoni
Drosophila mojavensis
Drosophila grimshawi
Culex quinquefasciatus
Anopheles gambiae
Tribolium castaneum
Apis mellifera
Bombus impatiens
Bombus terrestris
Nasonia vitripennis
Danaus plexippus
Strongylocentrotus purpuratus
Branchiostoma floridae
Ciona intestinalis
Oreochromis niloticus
Danio rerio
Xenopus tropicalis
Anolis carolinensis
Meleagris gallopavo
Gallus gallus
Monodelphis domestica
Mus musculus
Ailuropoda melanoleuca
Loxodonta africana
Callithrix jacchus
Pongo abelii
Homo sapiens
B
AID
C. elegans pol γ-α
H. sapiens pol γ-α
H. sapiens pol γ-α + proximal β

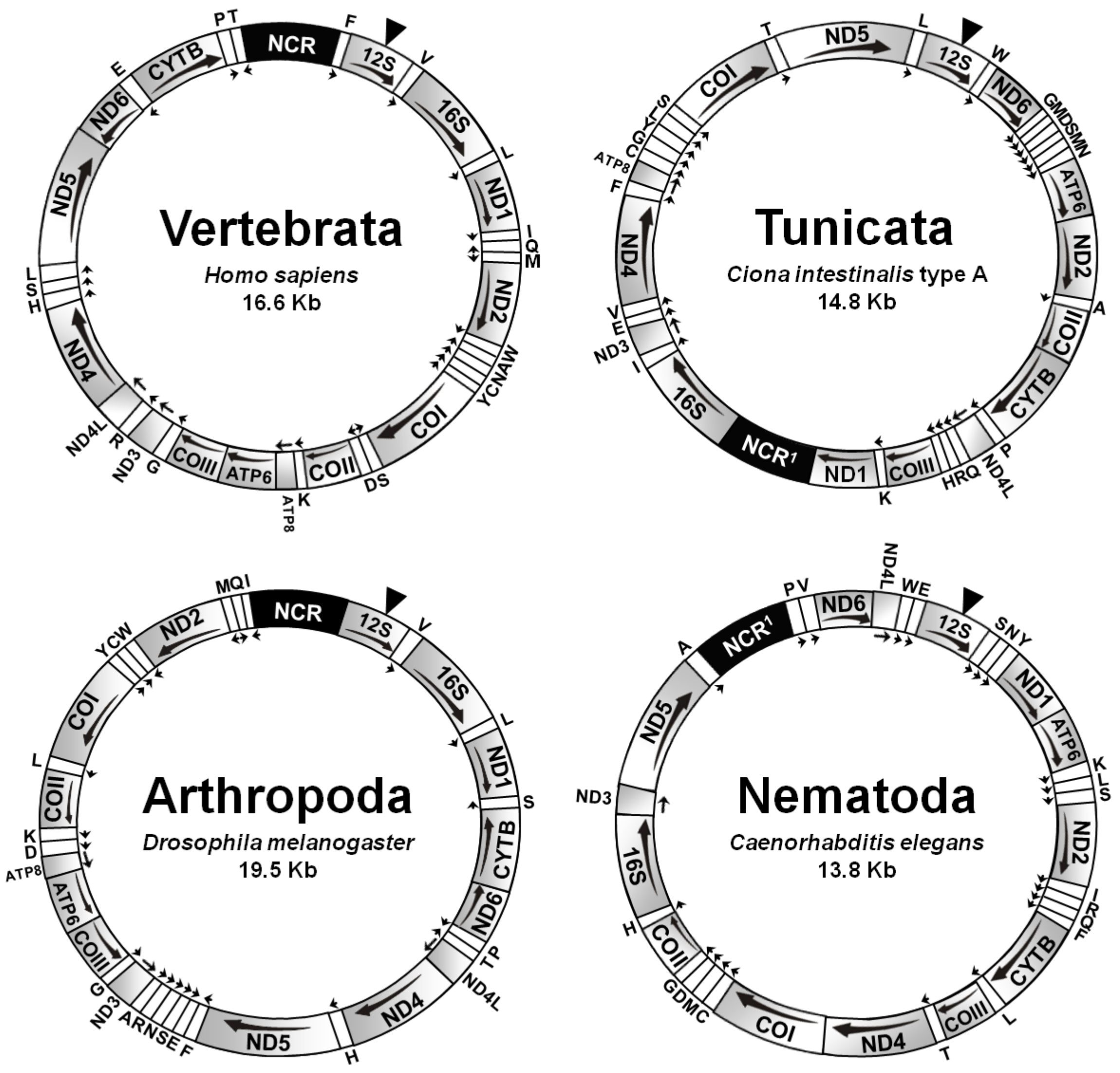

Vertebrata
Homo sapiens
16.6 Kb
NCR
12S
16S
ND1
ND2
COI
COII
ATP6
COIII
ND4
ND5
ND6
CYTB
PT
F
V
L
I
Q
M
YCNAW
DS
K
ATP8
G
ND3
R
ND4L
L
S
H
E
Tunicata
Ciona intestinalis type A
14.8 Kb
ND5
12S
ND6
ATP6
ND2
COII
CYTB
NCR[1]
ND1
COIII
16S
ND4
COI
T
L
W
GMDSMN
A
P
ND4L
HRQ
K
I
ND3
E
V
F
ATP8
C
G
Y
S
Arthropoda
Drosophila melanogaster
19.5 Kb
NCR
12S
16S
ND1
CYTB
ND6
ND4
ND5
COIII
ATP6
COII
COI
ND2
MQI
V
L
S
TP
ND4L
H
F
ARNSE
ND3
G
ATP8
D
K
L
YCW
Nematoda
Caenorhabditis elegans
13.8 Kb
NCR[1]
ND6
12S
ND1
ATP6
ND2
CYTB
COIII
ND4
COI
COII
16S
ND5
PV
ND4L
WE
SNY
K
L
S
I
R
Q
F
L
T
GDMC
H
ND3
A

Supplemental Figure Legends

Figure S1. Multiple amino acid sequence alignment using selected animal pol γ-α from our dataset retrieved from public databases (see Table S1). The outgroup sequence used here was the pol γ-α from the yeast *Saccharomyces cerevisiae*. The conserved Exo I-III and Pol A-C motifs are highlighted in yellow, red, magenta, light green, cyan and dark green, respectively.

Figure S2. Multiple amino acid sequence alignment using selected animal pol γ-β from our dataset retrieved from public databases (see Table S1). The outgroup sequence used here was the glycyl-tRNA synthetase from the bacterial species *Thermus thermophilus*.

Figure S3. Schematic representations of the mitochondrial genome architecture in vertebrates, tunicates, arthropods and nematodes. The mtDNA of humans (Anderson et al. 1981), *Ciona intestinalis* type A (Gissi et al. 2004), *Drosophila melanogaster* (Lewis et al. 1994 and 1995) and *Caenorhabditis elegans* (Okimoto et al. 1992) represent typical gene organizations found in their animal groups, respectively, and are oriented relative to the position of the 12S gene (black inverted triangles). The arrows below each gene indicate the direction of transcription. tRNA genes are indicated by one-letter symbols, and the 12S and 16S rRNA genes appear as 12S and 16S, respectively. The protein-coding genes are: ATP6 and 8, encoding ATPase subunits 6 and 8; CYTB, cytochrome oxidase b; COI-III, cytochrome *c* oxidase subunits I-III; ND1-6 and 4L, NADH dehydrogenase subunits 1-6 and 4L. The major non-coding region (NCR) of mtDNA, also known as the "D-loop" in humans (1122 bp) and "A+T-rich region" in *D. melanogaster* (4601 bp), are believed to contain most of the *cis*-elements that control initiation and/ or termination of mtDNA transcription and replication (Falkenberg, Larsson, Gustafsson 2007, Kasiviswanathan, Collins, Copeland 2012, Saito et al. 2005, Joers and Jacobs 2013). It has not yet been determined if the NCR[1] in the *C. intestinalis* (100 bp) and *C. elegans* (466 bp) mtDNAs are functional equivalents of the vertebrate and arthropod NCRs. Although there are other non-coding regions of similar sizes (85 bp between COIII and K) in the *C. intestinalis* mtDNA, the NCR[1] represents the largest one. The mtDNA molecules, tRNA genes, and NCRs are not represented to scale; protein-coding and rRNA genes are depicted to scale.
